# Estimating adjuvant treatment effects in Stage II colon cancer: Comparing the synthesis of randomized clinical trial data to real‐world data

**DOI:** 10.1002/ijc.32629

**Published:** 2019-08-31

**Authors:** Gabrielle Jongeneel, Thomas Klausch, Felice N. van Erning, Geraldine R. Vink, Miriam Koopman, Cornelis J.A. Punt, Marjolein J.E. Greuter, Veerle M.H. Coupé

**Affiliations:** ^1^ Department of Epidemiology and Biostatistics Amsterdam UMC, VU University Amsterdam The Netherlands; ^2^ Department of Research Netherlands Comprehensive Cancer Organisation (IKNL) Utrecht The Netherlands; ^3^ University Medical Center Utrecht Utrecht University Utrecht The Netherlands; ^4^ Department of medical oncology Amsterdam UMC, University of Amsterdam Amsterdam The Netherlands

**Keywords:** colon cancer, treatment effect, randomized clinical trial, real‐world data, adjuvant chemotherapy

## Abstract

There is an ongoing discussion regarding the impact of adjuvant chemotherapy in Stage II colon cancer. We therefore estimated adjuvant treatment effect in Stage II colon cancer using pooled disease‐free survival (DFS) data from randomized clinical trials (RCT approach) and compared this to real‐world data (RWD approach) estimates. First, we estimated the treatment effect in RCTs by (*i*) searching relevant trials reporting DFS data, (*ii*) generating patient‐level data from reported DFS data and (*iii*) estimating treatment effect in the patient‐level data. Second, the treatment effect was estimated in an observational cohort of 1,947 patients provided by the Netherlands Cancer Registry using three propensity score methods; matching, weighting and stratification. In the RCT approach, patient‐level data of 4,489 patients (events: 853) were generated from seven trials which compared two of the following treatment arms: control, 5FU/LV or FOLFOX. A Cox model was used to estimate a hazard ratio (HR) of 0.77 (0.43;1.10) for 5FU/LV *vs*. control and 0.93 (0.72;1.15) for FOLFOX *vs*. 5FU/LV. In the RWD approach, HRs for any adjuvant treatment *vs*. control were 0.95 (0.50;1.80), 0.88 (0.24;3.21) and 1.05 (0.04;2.06) using matching, weighting and stratification, respectively. There was no significant difference with the estimates from the RCT approach (interaction test, *p* > 0.10). The RCT data suggest a clinically relevant benefit of adjuvant chemotherapy in terms of DFS, but the estimate did not reach statistical significance. Stratified analyses are required to evaluate whether treatment effect differs in specific subgroups.

## Abstract 2

**What's new?**

There is an ongoing discussion regarding the impact of adjuvant chemotherapy in stage II colon cancer. This study presents the most recent pooled estimate based on available RCT data since 1999, resulting in a pooled hazard ratio of 0.77 (95% CI 0.43;1.10) for fluoropyrimidine compared to no treatment. Even though no significant treatment effect was found, neither in the RCT approach nor in the real‐world data approach, the RCT data suggest a clinically‐relevant benefit of adjuvant chemotherapy. To improve guidance in treatment decisions, larger sample sizes, pooling of true patient‐level data with covariate information, and subgroup specific analyses are required.

**Abbreviations**
DFS: disease‐free survival
GRADE: Grading of Recommendations Assessment, Development and Evaluation
HR: hazard ratio
NCR: Netherlands Cancer Registry
OS: overall survival
RCT: randomized clinical trial
RWD: real‐world data

## Introduction

There is an ongoing clinical dilemma of whether or not to provide adjuvant treatment to Stage II colon cancer patients after surgery. High‐risk patients who are eligible for adjuvant treatment are identified according to the European Society for Medical Oncology guidelines based on clinical and pathological factors. The most commonly used high‐risk factors include pT4 stage, less than 10–12 lymph nodes evaluated, the presence of perforation and/or obstruction, extramural vascular invasion, perineural invasion, poorly or undifferentiated tumor and mismatch repair status.^1^

Such guideline recommendations are developed using the Grading of Recommendations Assessment, Development and Evaluation (GRADE) approach. GRADE assigns most value to randomized clinical trials (RCTs) and meta‐analyses, because of the methodologically strong character.^2^ An essential advantage of RCTs is the unbiased estimation of the treatment effect due to randomized allocation which can balance both observed and unobserved confounders. This randomized allocation is often combined with strict inclusion and exclusion criteria to minimize bias in the evaluation of treatment response.^3^, ^4^ Because of this strict patient selection in RCTs, the patient population may differ in daily clinical practice. Thus, treatment effect in clinical practice may be different from an RCT‐based treatment effect.^3^, ^5^, ^6^

Observational studies without patient selection, although lower on the GRADE scale, are closer to clinical practice. In particular, after the introduction of electronic medical records, more and more scientists and decision makers are arguing for the use of observational studies in addition to RCTs.^4^, ^7^ Nationwide registries are an example of such observational studies and often contain many patients. However, comparing treatment groups in observational studies is challenging because selection bias arises as a result of the nonrandom treatment allocation.^4^, ^8^ For that reason, appropriate statistical methodology to correct for confounding by indication should be applied.^9^ Even then, cautious interpretation of the results is necessary. Despite these limitations, observational studies could give important insights into real‐world effectiveness of treatment regimens.^4^, ^10^, ^11^

In the field of colon cancer, the literature is contradictory regarding treatment effects based on RCTs compared to those based on real‐world oncology registry data. For example, Iwashyna *et al*.^12^ concluded that a comparable adjuvant treatment effect is found in real‐world data (RWD) and RCTs in Stage III colon cancer patients. On the other hand, Meyerhardt *et al*.^13^ concluded that in metastatic colorectal cancer patients, treatment effect based on RCTs seems much stronger than the effect estimated using registry data. The authors speculate that the main explanation for this difference in effect is uncorrectable heterogeneity between the populations at baseline.

In patients diagnosed with Stage II colon cancer, results from RCTs were not supportive of prescribing adjuvant chemotherapy to all patients.^14^, ^15^, ^16^, ^17^, ^18^, ^19^ For example, the IMPACT meta‐analysis showed in a pooled analysis a HR of 0.83 (90% CI 0.72;1.07) for disease‐free survival (DFS) and a HR of 0.86 (90% CI 0.68;1.07) for overall survival (OS) for fluoropyrimidine monotherapy compared to no treatment.^19^ Combination regimens in which oxaliplatin is given in addition to fluoropyrimidine were not included in IMPACT, although these combination regimens are nowadays recommended in Dutch and international guidelines.^1^, ^20^ Furthermore, the trials included in IMPACT were not optimally designed to determine the treatment effect in Stage II colon cancer as patients with rectal cancer or Stage III disease were included as well, leading to relatively few Stage II colon cancer patients.^21^

Therefore, the aim of our study was to estimate adjuvant treatment effects in Stage II colon cancer patients using pooled DFS data from RCTs. Given the dilemma regarding applicability of outcomes from RCTs to the real‐world population, our secondary aim was to compare the RCT estimates to estimates based on a national oncology registry.

## Methods

To estimate treatment effect in Stage II colon cancer patients, two approaches were used which we refer to as the “RCT approach” and “RWD approach.” In the RCT approach, we estimated the treatment effect in RCT data using the following three steps: (*i*) systematically searching relevant trials for which aggregated data on DFS was reported for Stage II colon cancer patients, (*ii*) generating patient‐level data from reported aggregated data and (*iii*) estimating a hazard ratio (HR) for treatment effect in the obtained patient‐level data. In the RWD approach, treatment effects for DFS were estimated in an observational cohort of 1,947 patients provided by the Netherlands Cancer Registry (NCR). Three methods were used to estimate the treatment effects: (*i*) propensity score matching (matching), (*ii*) inverse propensity score weighting (weighting) and (*iii*) propensity score stratification (stratification). All treatment effects were estimated using both parametric and semiparametric survival analyses. The semiparametric estimates were considered as main analysis. Supporting Information Table S1 describes the rationale, methods and results for the parametric analyses.

### RCT approach

#### *Systematical search for relevant trials*

Studies that used a RCT design were included when they compared an adjuvant treatment arm to another adjuvant treatment arm or to a control group, and when Stage II colon cancer patients were at least a subgroup of the included patients. In line with the Dutch and international guidelines applicable during the literature search, the included adjuvant treatment regimens had to have a duration of at least 6 months.^1^, ^20^ Only studies published after 1987 in Western countries were taken into account. Finally, a Kaplan–Meier curve stratified for Stage II colon cancer patients with DFS as outcome had to be reported, as well as the associated numbers at risk.

**Figure 1:**
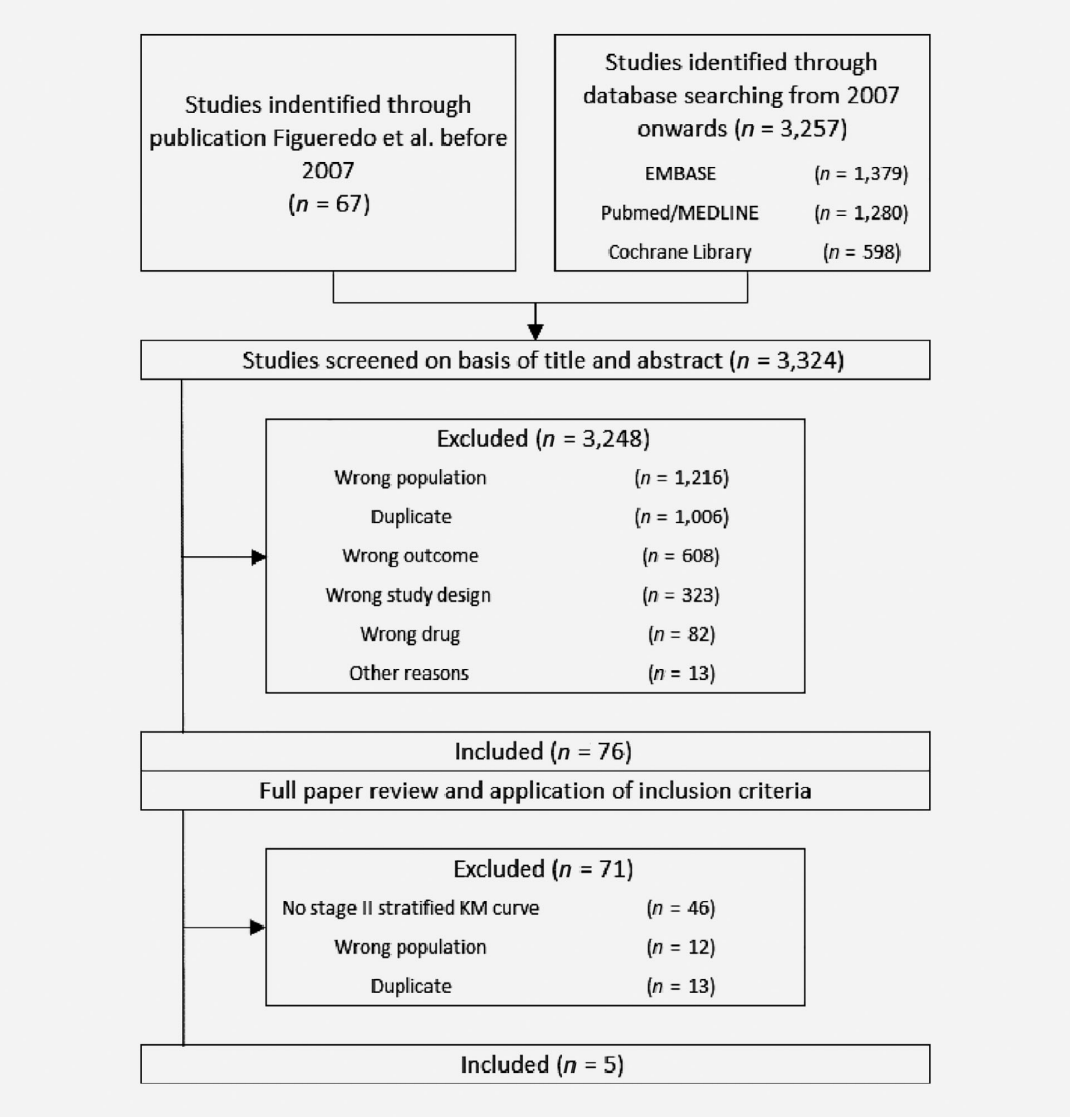
Flowchart of study selection.

For the identification of studies, we conducted a systematic search in MEDLINE, EMBASE and the Cochrane library similar to the search described in the Cochrane review by Figueredo *et al*.^22^ Reference lists of relevant studies were also searched and there were no language restrictions. A more detailed description of our search strategy is provided in Supporting Information. Inclusion criteria were applied by one researcher (GJ) to titles and abstracts. Full texts were obtained for hits that were considered relevant by one researcher (GJ). When in doubt, the eligibility was established by discussion (GJ, MG and VC). All authors agreed on the included studies before data extraction. Reasons for exclusion were documented and are presented in the PRISMA flow diagram (Fig. 1).^23^, ^24^ The main reasons for excluding studies were a population that did not include Stage II colon cancer patients or not reporting a stratified Kaplan–Meier curve for Stage II patients. Of each included trial, the following characteristics were extracted: time period, country, Stage II sample size, treatment regimens and 5‐year DFS (Tables 1 and 2). DFS was defined as the length of time after surgery during which no recurrence was detected.

**Table 1:** Trial and patient characteristics

	Recruitment			Eligibility criteria				Treatment				
	Date of first randomization	Total randomized	Stage II randomized	Tumor site	Age limit (years)	Tumor stage	Start therapy after surgery (days)	Comparator	Treatment	Number of cycles	Duration (months)	Median follow‐up (years)
IMPACT (1999)												
GIVIO	January 1989	888	449	Colon	None	II and III^1^	35	Control	FU + LV	6	6	5.3
NCIC‐CTG	May 1987	370	221	Colon	None	II and III^1^	56	Control	FU + LV	6	6	5.9
FFCD	October 1982	268	168	Colon	<75	II and III^1^	35	Control	FU + LV	6	6	5.2
NNCTG	February 1988	317	57	Colon	None	II and III^1^	35	Control	FU + LV	6	6	6.4
Sienna	January 1985	239	121	Colon	None	II and III^1^	21	Control	FU + LV	12	12	8.5
QUASAR (2007)	May 1994	3,239	2,963	Colon/Rectum	None	I, II and III	42	Control	FU + LV	6	6	5.5
Schippinger et al. (2007)	November 1993	500	500	Colon	<80	II	42	Control	5‐FU + LV	7	13	8.0
MOSAIC (2009)	October 1998	2,246	899	Colon	<75	II, III	42	LV5FU2	FOLFOX4	12	6	6.8
NSABP C07 (2011)	February 2000	2,492	695	Colon	None	II,III	NR	FULV	FLOX	3	6	8.0

1 Dukes classification was used in these studies. Stage II: Dukes B2, tumor has grown through the gut, but not yet in lymph nodes. Stage III: Dukes C, tumor has grown into the regional lymph nodes.

Abbreviations: FOLFOX, regimen that includes the drugs leucovorin, fluoropyrimidine and oxaliplatin; FU, fluoropyrimidine; LV, leucovorin; NA, not applicable; NR, not reported.

**Table 2:** Baseline characteristics of the patients included in the RCTs

	IMPACT		QUASAR		Schippinger *et al*.		MOSAIC		NSABP C07	
	FU + LV	Control	FU + LV	Control	5‐FU/LV	Control	FOLFOX4	LV5FU2	FOLFOX	FU + LV
5‐year DFS Stage II	0.77	0.74	0.80	0.77	0.85	0.80	0.83	0.80	0.83	0.80
Stage										
I	0 (0)	0 (0)	8 (1)	8 (1)	0 (0)	0 (0)	0 (0)	0 (0)	0 (0)	0 (0)
II	507 (100)	509 (100)	1,483 (91)	1,480 (91)	252 (100)	248 (100)	451 (40)	448 (40)	360 (29)	359 (29)
III	0 (0)	0 (0)	131 (8)	129 (8)	0 (0)	0 (0)	672 (60)	675 (60)	884 (71)	878 (71)
Site										
Colon	507 (100)	509 (100)	1,148 (71)	1,143 (71)	252 (100)	248 (100)	NR	NR	1,247 (100)	1,245 (100)
Left	256 (50)	280 (55)	NR	NR	122 (48)	124 (50)	NR	NR	247 (20)	263 (21)
Right	236 (47)	220 (43)	NR	NR	130 (52)	124 (50)	NR	NR	576 (46)	507 (41)
Recto sigmoid	NR	NR	NR	NR	NR	NR	NR	NR	412 (33)	459 (37)
Multiple	6 (1)	3 (1)	NR	NR	NR	NR	NR	NR	10 (1)	15 (1)
Unknown	9 (2)	6 (1)	NR	NR	NR	NR	NR	NR	0 (0)	0 (0)
Rectum	0 (0)	0 (0)	474 (29)	474 (29)	0 (0)	0 (0)	NR	NR	0 (0)	0 (0)
Gender										
Male	272 (54)	287(56)	1,006 (62)	973 (60)	137 (54.4)	134 (54.0)	630 (56)	588 (52)	690 (55.3)	(58)
Age	61 (22–79)	62 (26–86)	63 (23–84)	63 (23–86)	65 (29–79)	65 (30–80)	61 (NR)	60 (NR)	59 (NR)	59 (NR)
pT stage										
T2	0 (0)	0 (0)	NR	NR	0 (0)	0 (0)	51 (4)	54 (5)	NR	NR
T3	429 (85)	437 (86)	NR	NR	217 (86)	214 (86)	853 (76)	852 (76)	NR	NR
T4	9 (1)	6 (1)	NR	NR	35 (14)	34 (14)	213 (20)	208 (19)	NR	NR
Unknown	69 (14)	6 (13)	NR	NR	0 (0)	0 (0)	0 (0)	0 (0)	NR	NR
Tumor differentiation										
Moderate/well	432 (85)	421 (83)	NR	NR	NR	NR	934 (83)	914 (81)	NR	NR
Poor	51 (10)	63 (12)	NR	NR	NR	NR	141 (13)	148 (13)	NR	NR
Other	8 (2)	10 (2)	NR	NR	NR	NR	0 (0)	0 (0)	NR	NR
Unknown	16 (3)	15 (3)	NR	NR	NR	NR	47 (4)	61 (5)	NR	NR
Perforation present	NR	NR	NR	NR	NR	NR	77 (7)	77 (7)	NR	NR
Bowel obstruction	NR	NR	NR	NR	NR	NR	201 (18)	217 (19)	NR	NR

Baseline characteristics of Stage II patients were not reported separately in most of the included trials. For these studies, the table shows the baseline characteristics of all patients included in the trial. Data are presented as numbers (%) except for 5‐year DFS and age. For DFS, data were presented as proportion disease‐free. For age, data were presented as mean (range).

Abbreviations: DFS, disease‐free survival; FOLFOX, regimen that includes the drugs leucovorin, fluoropyrimidine and oxaliplatin; FU, fluoropyrimidine; LV, leucovorin; m, months; NR, not reported.

#### *Generating patient‐level data*

DFS for Stage II patients was extracted from the included publications. First, all data points from the Kaplan–Meier curves for DFS that were required to reproduce the figure were read using GetData Graph Digitizer 2.26.^25^ Then, a curve fitting approach developed by Hoyle and Henley^26^ was used to generate the patient‐level data including data on treatment (yes/no), recurrence (yes/no) and time to recurrence. To maintain the randomization of the original trials, the extracted patient‐level data for all included studies were pooled in two separate analyses: (*i*) an analysis of the trials that compared fluoropyrimidine monotherapy to a control arm and (*ii*) an analysis of the trials that compared fluoropyrimidine in combination with oxaliplatin to fluoropyrimidine monotherapy. The first analysis was considered as the main outcome. To assess whether the study populations in the pooled RCTs were homogeneous, we compared 5‐year DFS using an interaction test.^27^

#### *Estimating a HR for treatment effect*

Then, HRs for DFS were estimated by adding treatment as a covariate in a Cox model. To account for the potential heterogeneity between trials a multilevel Cox regression was conducted. Literature suggests that a HR of 0.80 or less may be considered clinically meaningful.^28^ Therefore, this threshold value was used to judge clinical relevance. All survival models were estimated using the coxme package in Rstudio version 3.4.2.^29^

### RWD approach

#### *Real‐world observational cohort*

The treatment effect was also estimated in a real‐world observational cohort from the NCR. The dataset consisted of 1,947 patients diagnosed with Stage II colon cancer between 2002 and 2008.^30^ The majority of patients had a pT3 stage (90.0%), less than 10 evaluated lymph nodes (53.9%) and a well/moderate tumor differentiation (83.3%). About 114 patients received adjuvant treatment (5.9%) and 1,833 did not (94.1%). Treatment regimens were fluoropyrimidine monotherapy (33.3%) or fluoropyrimidine with oxaliplatin (66.7%). Data were available on patient and tumor characteristics, time to recurrence and death. Follow‐up duration of the patients was at least 36 months, with a maximum of 179 months. The median follow‐up duration was 53 months for DFS. Recurrences could either be diagnosed due to symptoms or during regular follow‐up visits. This follow‐up consists of consultations every half‐year during the first 2–3 years after surgery and yearly thereafter until 5 years after surgery. In these consultations, either an ultrasound scan of the liver or CT scan of the abdomen is made. Also, the CEA values are determined at each visit.^20^ The baseline characteristics of the cohort are shown in columns 2–4 of Table 3.

**Table 3:** Patient characteristics of the observational NCR cohort

Variable	Whole population (*n* = 1,947)	Untreated (*n* = 1,833)	Treated (*n* = 114)	*t*‐test *p*‐value treated/untreated	Untreated match 1^1^ (*n* = 76)	Treated match 1^1^ (*n* = 76)	Untreated match 2^2^ (*n* = 113)	Treated match 2^2^ (*n* = 113)
Age (years)	70.9 (11.0)	71.5 (10.8)	61.7 (10.6)	<0.01	64.0 (8.2)	64.0 (8.2)	59.5 (11.6)	61.9 (10.0)
pT stage								
T3	1,753 (90.0)	1,678 (91.5)	75 (65.8)	<0.01	68 (89.5)	68 (89.5)	85 (75.2)	75 (66.4)
T4	194 (10.0)	155 (8.5)	39 (34.2)		8 (10.5)	8 (10.5)	28 (24.8)	38 (33.6)
Number of evaluated lymph nodes								
<10	1,050 (53.9)	975 (53.2)	75 (65.8)	0.01	57 (75.0)	57 (75.0)	82 (72.6)	75 (66.4)
≥10	897 (46.1)	858 (46.8)	39 (34.2)		19 (25.0)	19 (25.0)	31 (27.4)	38 (33.6)
Tumor site								
Right	1,173 (60.2)	1,106 (60.3)	67 (58.8)	0.82	43 (56.6)	43 (56.6)	65 (57.5)	67 (59.3)
Left	774 (39.8)	727 (39.7)	47 (41.2)		33 (43.4)	33 (43.4)	48 (42.5)	46 (40.7)
Tumor differentiation								
Well/moderate	1,623 (83.3)	1,543 (84.2)	80 (70.2)	<0.01	58 (76.3)	58 (76.3)	89 (78.8)	79 (69.9)
Poor/not	324 (16.7)	290 (15.8)	34 (29.8)		18 (23.7)	18 (23.7)	24 (21.2)	34 (30.1)

Data are presented as means (±SD) or numbers (%).

1 Match 1 is the matching sample with caliper score of 0.

2 Match 2 is the matching sample with caliper score of 0.2 multiplying by standard deviation of the logit propensity score.

#### *Propensity score risk adjustments*

Due to the nonrandomized nature of the cohort, comparisons between patients who received adjuvant chemotherapy with nonrecipients are potentially biased due to differences between both groups at baseline, that is, confounding by indication. The use of specialized methods is necessary to correct for confounding by indication. Although there are several methods available, there is no consensus on a gold standard.^8^ Therefore, three methods were used to estimate the treatment effects: (*i*) propensity score matching (matching), (*ii*) inverse propensity score weighting (weighting) and (*iii*) propensity score stratification (stratification). The choice for these methods is in line with Austin *et al*. (2013) and Gayat *et al*. (2012) who showed that these methods have a good performance for time‐to‐event data.^31^, ^32^ Propensity score estimation using observed confounders that are determined prior to treatment administration allows for unbiased estimation of treatment effects under the assumption of no unobserved confounding.^33^ This assumption cannot be formally tested. However, the most relevant clinical and pathological correlates of treatment assignment and survival, as reported in the literature, were available in our dataset (i.e., gender, age, pT stage, differentiation grade, lymph nodes evaluated and tumor site).^34^

The propensity score represents the probability that a patient would receive adjuvant treatment. Propensity scores were determined on the basis of a logistic regression model in which the dependent variable was administration of adjuvant treatment and the independent variables were all available factors potentially associated with administration of adjuvant treatment (gender, age, pT stage, number of evaluated lymph nodes, grade of differentiation and tumor site). Interactions between treatment and any of these factors were included as well. A backward variable selection based on Akaike Information Criterium (AIC) was done to select the most relevant covariates in the propensity score model.

#### *Survival models*

First, the naïve treatment effect, that is, without correcting for confounding by indication, was estimated in the observational cohort. Second, HRs for DFS were estimated including a correction for confounding by indication by matching, weighting and stratification. In the matching method, patients who did receive adjuvant treatment were 1:1 matched based on the propensity score to patients who did not receive adjuvant treatment. A caliper score was used to determine the maximum deviation in propensity score for matched pairs. In the inverse propensity score weighting method treatment effects were estimated by weighting the individuals based on the propensity score. In the stratification method, the sample was stratified into five mutually exclusive subclasses based on the propensity score. A detailed description of the confounding by indication methods is provided in Supporting Information.

#### *Model selection*

For each Cox model, a forward covariate selection was performed. All multivariate survival models included the covariates that were significant in one or more survival models to ensure comparability of the results: age, pT stage, evaluated lymph nodes, tumor site and differentiation grade.

### Comparison RCT approach and RWD approach

An interaction test was used for significance testing of the differences between the estimates based on the RCT and RWD approach.^27^ A significance threshold of *p* < 0.10 was used to avoid type II error rate.

### Data availability

The generated patient‐level data used for the RCT approach is available as Supporting Information. The registry data that support the findings of the RWD approach in this study are upon request available from the NCR.

## Results

### RCT approach

#### *Eligible studies*

We identified 3,324 potentially eligible studies that provided survival data on DFS for Stage II colon cancer patients. Of these, five publications met the inclusion criteria. The five publications reported nine trials which were included in the current study.^16^, ^17^, ^19^, ^35^, ^36^ Four of the five included publications were RCTs and one was a meta‐analysis of five RCTs.

#### *Study characteristics*

Characteristics of the nine included studies are presented in Table 1. The studies were published between 1999 and 2011. Five studies originated from European centers, two from North America and two included patients from multiple Western countries. Comparison of baseline characteristics was hampered, because three of the five publications did not report baseline characteristics for Stage II colon cancer separately. The total sample size of Stage II patients in the nine included studies was 6,076 patients.

#### *Pooled treatment groups*

Included trials either compared fluoropyrimidine monotherapy to a control arm, that is, IMPACT, QUASAR and Schippinger *et al*. or fluoropyrimidine in combination with oxaliplatin to fluoropyrimidine monotherapy, that is, NSABP C07 and MOSAIC. To maintain the randomization of the trials, HRs were estimated in two separate pooled analyses. In the first analysis, we pooled trials that compared fluoropyrimidine monotherapy to a control arm whereas, in the second analysis, we pooled trials that compared fluoropyrimidine in combination with oxaliplatin to fluoropyrimidine monotherapy. The 5‐year DFS of the pooled studies were in the same range (Table 2). Based on the interaction test, no significant differences were found in 5‐year DFS between the pooled study arms (Supporting Information Table S2).

#### *Survival analyses*

**Figure 2:**
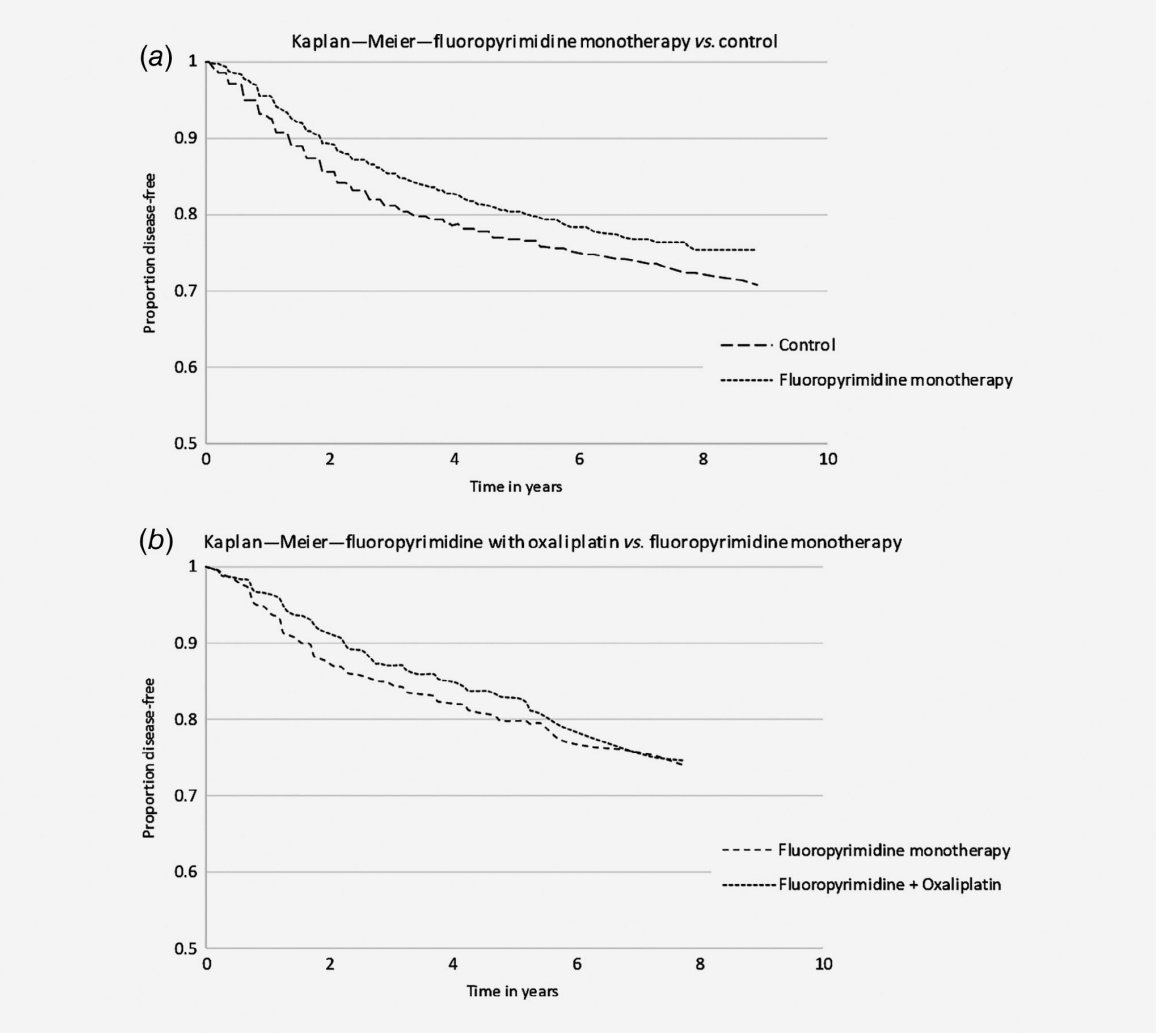
Kaplan–Meier curves for the treatment and control groups for pooled population 1 (*a*) and the curves for fluoropyrimidine combined with oxaliplatin compared to fluoropyrimidine monotherapy in pooled population 2 (*b*).

The Kaplan–Meier curves for DFS are shown by trial arm in Supporting Information Figure S1 *a* for the fluoropyrimidine monotherapy group which was compared to the control group, in Supporting Information Figure S1 *b* for the control group, in Supporting Information Figure S1 *c* for the combination therapy group and in Supporting Information Figure S1 *d* for the fluoropyrimidine monotherapy group which was compared to the combination therapy group. In the population for the first pooled analysis, there were 454 recurrences among the 2,244 patients in the control group after 5 years of follow‐up. For the treatment group with fluoropyrimidine monotherapy, the number of recurrences was 399 in a population of 2,245 patients. A pooled Kaplan–Meier curve is shown in Figure 2 *a*. In the population for the second pooled analysis, there were 175 recurrences among 788 patients in the fluoropyrimidine monotherapy group. In the group which received fluoropyrimidine in combination with oxaliplatin the number of recurrences was 166 in 799 patients. A pooled Kaplan–Meier curve is shown in Figure 2 *b*. The HR for treatment effect in the first pooled analysis, fluoropyrimidine compared to no treatment, was 0.77 (95% CI 0.43;1.10) for DFS. In the second pooled analysis, in which fluoropyrimidine combined with oxaliplatin was compared to fluoropyrimidine monotherapy, a HR of 0.93 (95% CI 0.72;1.15) was found for DFS. Both treatment effects were estimated with a multilevel Cox model. HRs for the multilevel and non‐multilevel Cox survival models are shown in Table 4.

**Table 4:** Estimated Treatment effects

	Cox proportional hazard		
	Hazard ratio (95% CI)	*p*‐value	*p*‐value of comparison RWD to RCT
RCT approach—trial data			
Survival model 1	0.78 (0.68;0.89)	<0.01	NA
Multilevel survival model 1	**0.77 (0.43;1.10)**	0.13	Comparator
Survival model 2	0.93 (0.76;1.16)	0.55	NA
Multilevel survival model 2	**0.93 (0.72;1.15)**	0.55	NA
RWD approach—observational data unadjusted			
Naive survival model	1.65 (1.13;2.42)	0.01	0.12
RWD approach—observational data adjusted based on propensity scores			
*PS matching—caliper 0*			
Multivariate survival model	0.95 (0.50;1.80)	0.88	0.49
*PS matching—caliper 0*.2*SD logit propensity			
Multivariate survival model	1.00 (0.58;1.70)	0.98	0.41
*PS inverse weighting*			
Multivariate survival model	0.88 (0.24;3.21)	0.99	0.71
*PS stratification*			
Multivariate survival model	1.05 (0.04;2.06)	0.99	0.47

Survival model 1 refers to the analysis in which a treatment effect was estimated for a fluoropyrimidine regimen compared to control. IMPACT, QUASAR and Schippinger *et al*. were included in this analysis. Survival model 2 refers to the analysis in which a treatment effect was estimated for fluoropyrimidine in combination with oxaliplatin compared to fluoropyrimidine monotherapy. MOSAIC and NSABP C07 were included in this analysis. Bold results are considered as main results.

Abbreviations: NA, not applicable; PS, propensity score; RCT, randomized clinical trial; RWD, real‐world data; SD, standard deviation.

### RWD approach

Table 4 shows the treatment effect estimates based on the NCR cohort. The unadjusted naive Cox model estimated an HR of 1.65 (95% CI 1.13;2.42, *p* = 0.01) for DFS.

#### *Propensity score risk adjustments*

Adjuvant treatment propensity scores ranged from <0.01 to 0.84 in the control group and from 0.01 to 0.89 in the treated group. Thus, the treatment group showed sufficient overlap with the control group. Means of the distribution of the confounders age, pT stage, evaluated lymph nodes, tumor site and differentiation grade were equal in the matched sample, after weighting, and in all propensity score strata.

For matching, weighting and stratification, both univariate and multivariate survival models were fitted. Below, only HRs for DFS of the multivariate survival models are described as these are considered as most reliable. Results of the univariate models are shown in Supporting information Table S3. For the matching method, two samples were defined: (*i*) a sample of 76 matched pairs based on a caliper score of 0 and (*ii*) a sample of 112 matched pairs based on a caliper score of 0.2 of the standard deviation of the logit propensity score (Table 3). Estimated multivariate HRs were 0.95 (95% CI 0.50;1.80) and 1.00 (95% CI 0.58;1.70), respectively. For the weighting method, a HR of 0.88 (95% CI 0.24;3.21) was estimated in a multivariate survival model. A pooled HR of 1.05 (95% CI 0.04;2.06) was found for the stratification method (Table 4). It should be noted that all confidence intervals are wide and do not reach clinical relevance or statistical significance.

### Comparison RCT and RDW approach

In Table 4, the *p* values of the interaction tests are shown. No significant differences were found between the estimates based on the RCT and RDW approach, which is potentially due to the small sample size of treated patients in the RWD approach. Furthermore, the estimate derived from the RCT approach suggests a clinically relevant treatment effect, while the treatment effect found in the RWD approach was not clinically relevant.

## Discussion

The primary aim of this study was to estimate adjuvant treatment effects for DFS in Stage II colon cancer patients using pooled summary survival data from RCTs (RCT approach). Given the dilemma regarding the applicability of outcomes from RCTs to the real‐world population, our secondary aim was to compare the RCT estimate to estimates based on a national oncology registry (RWD approach). The RCT approach resulted in a HR of 0.77 (95% CI 0.43;1.10) for fluoropyrimidine monotherapy compared to no treatment. In addition, a HR of 0.93 (95% CI 0.72;1.15) was found for fluoropyrimidine in combination with oxaliplatin compared to fluoropyrimidine monotherapy. Although point estimates from the separate studies as well as our pooled estimate suggests a clinically relevant benefit (HR < 0.80) in terms of DFS from adjuvant chemotherapy in Stage II colon cancer, no statistical significance was reached. For the RWD approach, in which we compared patients treated with adjuvant chemotherapy (33.3% fluoropyrimidine monotherapy and 66.6% fluoropyrimidine therapy with oxaliplatin) to patients who did not receive treatment, none of the applied propensity score methods resulted in a clinically relevant or significant treatment effect. Finally, no significant differences were found between estimates based on the RCT and RWD approach. It should be noted that the sample size of the cohort used for the RWD estimates was small, resulting in large confidence intervals. This is also the likely explanation for insignificance of the interaction test on the difference between estimates based on the RCT and RWD approach. Overall, no significant treatment effect was found, neither in the RCT approach nor in the RWD approach. Nevertheless, the point estimate in the RCT approach suggests a clinically relevant benefit of adjuvant chemotherapy. To improve guidance in adjuvant treatment decisions in Stage II colon cancer, larger sample sizes, the pooling of true patient‐level data with covariate information and/or subgroup‐specific analyses are required.

The nonsignificant result for the comparison of fluoropyrimidine to control in the RCT approach is probably related to the following two aspects. First, we used a multilevel Cox proportional hazard model to estimate treatment effect. The variance in a multilevel model consists of two components; the variance around the treatment effect and the variance around the added random effect.^37^ As a result, the variance is increased compared to a non‐multilevel approach. This is underlined by our results in which the multilevel estimate of 0.77 has a much wider confidence interval (95% CI 0.43;1.10, *p* = 0.13) than the non‐multilevel estimate of 0.78 (0.68;0.89, *p* < 0.01). Second, we did not have the original patient‐level data to our disposition. Therefore, we opted for using a curve fitting approach developed by Hoyle and Henley. This method precludes the inclusion of relevant covariates in the survival models, which may narrow down confidence intervals around the estimate for treatment effect. Moreover, it should be noted that statistical inference is increasingly questioned in the literature.^38^, ^39^ That is, a *p*‐value does not measure the size of an effect nor the importance of a result. Results should always be interpreted within their context; taking into account the sample size, the methods used to estimate the effect as precisely as possible, and relevance of a result in daily clinical practice.^28^ Taking these arguments into account, we would consider the treatment effect found in the RCT approach as clinically relevant.

In the current study, DFS was used as outcome measure to estimate treatment effect. Although OS is acknowledged as the gold standard outcome in cancer trials,^40^ DFS can be considered as a surrogate endpoint for OS. This is underlined by Sargent *et al*.^41^ who showed that DFS and OS are highly correlated in colon cancer trials evaluating fluorouracil‐based regimens in Stage III colon cancer patients. Also, the majority of trials included in the current study reported similar results for DFS and OS in terms of clinical relevance and statistical significance. Only in Schippinger *et al*.,^35^ the results for DFS and OS were not consistent in terms of clinical relevance, which may be explained by the small sample size.

Previously, Sargent *et al*.^42^ reported a relationship between treatment effect and microsatellite instability (MSI). In addition to improved prognosis, it was shown that patients with an MSI tumor have a certain resistance to fluoropyrimidine‐based chemotherapy. It is possible that, in addition to MSI, more molecular characteristics can be identified that may influence the impact of treatment on DFS. Therefore, a stratified analysis would presumably have increased the ability to detect a stronger treatment effect in specific subgroups, such as patients with a MSS. Besides, data from other retrospective analyses strongly suggest pT4 as the strongest prognostic factor in Stage II colon cancer.^43^, ^44^ However, stratified analysis for both predictive and prognostic factors was hampered due to the absence of covariate information in the patient‐level data for our RCT‐based analysis. In the RWD approach, a stratified analysis was hampered due to the small sample size.

The presented treatment effect estimate for fluoropyrimidine monotherapy compared to no treatment, is based on updating the IMPACT analysis with the trials of QUASAR and Schippinger *et al*. It should be noted that in the Sienna trial (included in the IMPACT meta‐analysis) and the trial of Schippinger *et al*. a deviant treatment regimen of 12 or 13 months was prescribed instead of 6 months as in the other trials included in this study.^19^, ^35^ We believe that this did not influence our results as the treatment effect for the Sienna trial and the trial of Schippinger *et al*. were in the same range as the other trials included in the current study (range 0.69–0.83). Furthermore, we estimated a treatment effect for fluoropyrimidine combined with oxaliplatin compared to fluoropyrimidine monotherapy. No significant differences were found in DFS for the addition of oxaliplatin. This finding is in contrast with the effect of adding oxaliplatin to adjuvant treatment in Stage III colon cancer patients; both NSABP C07 and MOSAIC found a significant improvement in DFS for the addition of oxaliplatin in Stage III colon cancer.^17^, ^36^ Yothers *et al*. (2011) suggests that this difference found in effect between Stage II and Stage III patients can be explained by (*i*) the smaller sample size for Stage II patients compared to Stage III patients and (*ii*) the higher absolute survival probability for Stage II patients.

In the RWD approach, there was an imbalance between treated (*n* = 114) and untreated patients (1,833). The explanation for the imbalance is two‐sided; first, the majority of the population is not high‐risk and therefore not eligible for adjuvant chemotherapy according to guidelines. Second, only 5% of all high‐risk patients received chemotherapy in the dataset. From literature, we know that most guideline deviations are well‐substantiated, for example, due to the poor clinical condition of the patient or a patients’ preference. Other possible explanations mentioned in the literature are unfamiliarity with the guideline and differences in expert opinions.^45^ Furthermore, data for DFS is often not collected by default in registry data. Therefore, only a small subset (*n* = 1,947) out of approximately 10,000 Stage II cancer patients, was available to estimate the effect of treatment on recurrence. The small number of treated patients was a serious limitation in this analysis, causing large variance around the treatment effect estimates from the RWD approach compared to the RCT approach. These wide confidence intervals limit the power of the interaction test to detect a significant difference between the RCT and RWD approach, even though we used a lenient significance threshold of *p* < 0.10.

In observational data such as national registry data, there is the potential for bias due to confounding by indication. In this study, we used appropriate, though complex methods to correct for this bias. These methods assume that there are no unmeasured confounders. In the NCR dataset, the most important clinical and pathological factors that determine treatment allocation were included (i.e. gender, age, pT stage, differentiation grade, lymph nodes evaluated and tumor site). Nevertheless, the patient‐related factor performance status was not measured, while this variable is reported to affect treatment allocation.^46^ Furthermore, results of the RWD analysis were not entirely representative of the original sample. For example, in the matching analysis, many of the nontreated patients were excluded due to the differences in sample size between the treated and nontreated group. Moreover, the estimates of the three methods used in the RWD analysis were not consistent in effect direction, which complicates the interpretation. In summary, results of our RWD analysis should be interpreted carefully, taking the limitations of the study design and the statistical methods into account.

To summarize, the RCT data suggest a clinically relevant benefit of adjuvant chemotherapy in terms of DFS, although this benefit was not significant in our pooled analyses. To improve guidance in adjuvant treatment decisions in Stage II colon cancer, future studies should focus on the pooling of true patient‐level data with covariate information and/or subgroup‐specific analyses.

## Supporting information


**Appendix S1**: Supplementary Information

**Appendix S2**: Supplementary Information
