# Supplementary material for: Estimating adjuvant treatment effects in Stage II colon cancer: Comparing the synthesis of randomized clinical trial data to real‐world data
Source: Int J Cancer. 2019 Aug 31;146(11):2968–78. doi: 10.1002/ijc.32629 (PMC7187209; doi:10.1002/ijc.32629)
Supplement: Supplementary file 1 — Appendix S1: Supplementary Information [file IJC-146-2968-s001.doc]

**Appendix 1. Parametric estimates**

*Rationale*

In our study all analyses were conducted both parametrically and semi-parametrically. Although non-parametric or semi-parametric methods are more commonly used, parametric estimates have the advantage that they can serve as an input for cost-effectiveness analyses (CEAs). In a parametric survival model, it is assumed that the survival curve corresponds to a certain mathematical distribution. This is important because many CEAs analyze both effects and costs using a lifelong time horizon. Non-parametric or semi-parametric approaches generate output that is difficult to use for extrapolation, whereas this is straightforward when a fully parametrized mathematical function is obtained.

*Parametric analyses*

We estimated hazard ratios (HRs) by adding treatment as a covariate to the parametric survival model. Various parametric survival distributions i.e. Weibull, log-logistic, log-normal and Gompertz were fitted. Final choice of distribution for the parametric survival model was based on AIC and visual fit.^1, 2^ All survival models were estimated using the survival and flexsurvreg package in Rstudio version 3.4.2 ^3, 4^. Note that it was not possible to estimate e a mixed effect Gompertz model due to limitations in R. We therefore used a non-mixed effect Gompertz model.

Appendix table 1 shows the results of the parametric analyses. The treatment effect estimates using a fully parametric approach were in line with the Cox estimates.

*Input for PATTERN model*

The parametric estimates derived from this study will be used as input for the Personalized Adjuvant TreaTment in EaRly stage coloN cancer (PATTERN) decision model. This model is developed to improve the selection of stage II colon cancer patients who benefit from adjuvant chemotherapy. PATTERN model predictions will provide more insight in the most cost-effective manner to allocate adjuvant chemotherapy in stage II colon cancer patients.

**Appendix Table 1.** Shape and rate parameters for the estimated Gompertz distributions and corresponding hazard ratios.

|  | Shape  (95% CI) | Rate  (95% CI) | Hazard ratio  (95% CI) |
| --- | --- | --- | --- |
| RCT approach – trial data |  |  |  |
| Survival model 1  Survival model 2 | -0.223 (-0.260;0.187)  -0.097 (-0.155; -0.040) | 0.090 (0.079;0.100)  0.057 (0.046;0.070) | 0.779 (0.682;0.889)  0.939 (0.759;1.161) |
| RWD approach – observational data unadjusted | |  |  |
| Naive survival model | -0.018 (-0.024;-0.013) | 0.005 (0.004;0.006) | 1.684 (1.148;2.469) |
| RWD approach – observational data adjusted based on propensity scores | |  |  |
| *PS matching - caliper 0* |  |  |  |
| Survival model | -0.025 (-0.042;-0.008) | 0.010 (0.005;0.003) | 0.932 (0.493;1.763) |
| Multivariate survival model | -0.024 (-0.041;-0.007) | 0.000 (0.000;0.011) | 0.946 (0.499;1.794) |
| *PS matching - caliper 0.2 * ds logit PS* | |  |  |
| Survival model | -0.029 (-0.044;-0.014) | 0.010 (0.006;0.016) | 1.127 (0.663;1.913) |
| Multivariate survival model | -0.027 (-0.043;-0.012) | 0.001 (0.000;0.004) | 1.006 (0.588;1.723) |
| *PS inverse weighting* |  |  |  |
| Survival model | -0.032 (-0.032;-0.031) | 0.008 (-0.064;0.080) | 0.852 (0.220;3.298) |
| Multivariate survival model | -0.031 (-0.031;-0.030) | 0.468 (-1.072;2.011) | 0.839 (0.241;2.917) |
|  |  |  |  |
| *PS stratification* |  |  |  |
| Survival model | -0.031 (-0.061;-0.001) | -0.008 (-0.111;-0.128) | 1.356 (0.345;2.366) |
| Multivariate survival model | -0.028 (-0.057;0.002) | 0.002 (-0.129;0.133) | 1.098 (0.059;2.137) |

Survival model 1 refers to the analysis in which a treatment effect was estimated for fluorouracil regimen compared to control. IMPACT, QUASAR and Schippinger et al. were included in this analysis. Survival model 2 refers to the analysis in which a treatment effect was estimated for fluorouracil in combination with oxaliplatin compared to fluorouracil monotherapy. MOSAIC and NSABP C07 were included in this analysis. Abbreviations: RCT = randomized clinical trial; RWD = real world data; PS = propensity score; sd= standard deviation**.**

**Appendix 2.** **Search method**

*Search methods for identification of studies*

In 2007, a Cochrane review was conducted on adjuvant therapy in completely resected stage II colon cancer patients.^5^ The aim of this review was to determine the effect of treatment on disease-free survival (DFS) in patients with stage II colon cancer. This Cochrane review used the same inclusion criteria as used for the current study, with the exception of the requirement to present a Kaplan Meier curve stratified for stage II CC patients and duration of the chemotherapy. Therefore, we screened the included studies in this Cochrane review by Figueredo et al. (2008)^5^ for identification of relevant studies published before 2007. To complement this selection with more recent publications, the search strategy published in Figueredo et al. was used to identify studies published between January 2007 and July 2018. We searched in accordance with Figueredo et al. in MEDLINE, EMBASE and the Cochrane library. Reference lists of relevant studies were also searched. There were no language restrictions.

*Search strategy Figueredo et al. (2008)*

1. Colonic Neoplasms/ [MeSH]

2. Colorectal Neoplasms/ [MeSH]

3. (colon OR colorectal OR colonic).ti.[title]

4. (malignan$ OR Neoplas$ OR cancer OR carcinoma OR adenocarcinoma).

5. 3 AND 4

6. 1 OR 2 OR 5
7. Chemotherapy, Adjuvant/ [MeSH]
8. adjuv$.mp.
9. 7 OR 8
10. 6 AND 9
11. (clinical AND trial).ab.ti. [abstract or title]
12. Clinical Trials/ [MeSH]
13. clinical trial.pt [publication type]
14.random$.mp.
15.Random Allocation/ [MeSH]
16.therapeutic use$.mp.
17. 11 OR 12 OR 13 OR 14 OR 15 OR 16
18. 10 and 17

**Appendix 3.** **Detailed description of confounding by indication methods**

*Average treatment effect of the treated*

The secondary aim of this study was to compare the treatment effect obtained with the RCT approach to estimates based on real world data. The RWD treatment effect was estimated as the treatment effect for the treated; that is, both treated and untreated patients were included in the analysis, but the treatment effect was defined across patients having been assigned to adjuvant treatment in the observational data.

This choice was mainly underpinned by the fact that there was a difference in age between the treated and non-treated population in the NCR cohort (Table 3). The age of the treated population in the NCR cohort was in line with the average age of the participants in the included trials, whereas the untreated population in the NCR cohort was considerably older (Table 2 and 3). It is known from the literature that younger patients are more likely to be eligible for adjuvant treatment in clinical practice.^6^ Therefore, patients treated with adjuvant therapy in the observational cohort were expected to be more comparable to patients included in the RCTs than those patients that did not receive adjuvant treatment.

*Double robustness property*

For the methods used, i.e. matching, weighting and stratification, we additionally used covariate-based regression-adjustment. Weighted regression with covariate adjustment possesses the double-robustness property which offers protection against model-misspecification.^7^ Performing covariate adjustment in matched or stratified samples can also lead to more precise estimates and is advised by some scholars.^8, 9^ Therefore, the multivariate models were considered as main analysis in the current study.

*Matching*

Propensity score matching allows for matching of treated and untreated patients who have similar propensity scores.^10^ Patients who did not receive adjuvant treatment were 1:1 matched based on the logit of the propensity score to patients who did receive adjuvant treatment. In line with Austin et al. (2011), we used a caliper width equal to 0.2 of the standard deviation of the logit of the propensity score ^11^ as well as a caliper width equal to 0. Once a propensity score matched sample was defined, a Cox model was fitted on the matched cohort to compare recurrence rates between those patients who did and those who did not receive adjuvant treatment. All variance estimations were done as described in the coxme packages in Rstudio.^3, 4, 12^

*Weighting*

Inverse propensity score weighting is a method in which treatment effects are estimated by weighting the individuals based on the propensity score. This corrects for the systematic differences between treated and untreated patients. To estimate the treatment effect for the treated, the propensity score weights were defined as w(x) = 1 for the treatment group and as w(x) = p(x)/(1-p(x)) for the control group, where w(x) is the weight for each individual and p(x) is the propensity score.^13, 14^ A weighted Cox model was fitted to compare recurrence rates between those patients who did and those who did not receive adjuvant treatment. In the weighted method, in addition to uncertainty surrounding the estimated HRs, there is uncertainty around the weights assigned to each individual as well.^15^ To account for this additional uncertainty, the analysis was bootstrapped using 1000 resamples, where the weights were re-estimated upon each bootstrap draw.

*Stratification*

In the stratification method, the sample was stratified into five mutually exclusive subclasses based on the propensity score, which is reported to eliminate 90% of the bias due to measured confounding variables when estimating a linear treatment effect.^9, 16^ Propensity score bins were defined such that the number of treated patients in each stratum was equal to assure stability of variance estimates. Cox models were used to compare recurrence rates between those patients who did and those who did not receive adjuvant treatment in each stratum.^17^ The pooled HR was obtained by taking the sum of the weighted estimates in each stratum, where weights denoted the ratio of the treated patients in that stratum divided by the total treated patients.

| **Appendix table 2**. Interaction test to compare 5-year DFS in pooled study arms   |  | IMPACT | QUASAR | Schippinger et al. | | --- | --- | --- | --- | | Survival model 1 - Control |  |  |  | | IMPACT | NA |  |  | | QUASAR | 0.39 | NA |  | | Schippinger et al. | 0.40 | 0.29 | NA | |  |  |  |  | | Survival model 1 - FU | IMPACT | QUASAR | Schippinger et al. | | IMPACT | NA |  |  | | QUASAR | 0.36 | NA |  | | Schippinger et al. | 0.30 | 0.18 | NA | |  |  |  |  | | Survival model 2 - FU | MOSAIC | NSABP C07 |  | | MOSAIC | NA |  |  | | NSABP C07 | 0.56 | NA |  | |  |  |  |  | | Survival model 2 FU + Oxaliplatin | MOSAIC | NSABP C07 |  | | MOSAIC | NA |  |  | | NSABP C07 | 0.66 | NA |  | | Abbreviations: NA=not applicable, FU=Fluorouracil. Survival model 1 refers to the analysis in which a treatment effect was estimated for a capecitabine or fluorouracil regimen versus no treatment. IMPACT, QUASAR and Schippinger et al. were included in this analysis. Survival model 2 refers to the analysis in which a treatment effect was estimated for capecitabine or fluorouracil regimen with oxilaplitin compared to capecitabine or fluorouracil monotherapy. P-value of the Altman interaction test is given for all combinations of pooled treatment arms. | | | |   **Appendix Table 3.** Estimated treatment effects without adding covariates to the Cox survival models in the RWD approach | | | |  |
| --- | --- | --- | --- | --- | --- | --- | --- | --- | --- | --- | --- | --- | --- | --- | --- | --- | --- | --- | --- | --- | --- | --- | --- | --- | --- | --- | --- | --- | --- | --- | --- | --- | --- | --- | --- | --- | --- | --- | --- | --- | --- | --- | --- | --- | --- | --- | --- | --- | --- | --- | --- | --- | --- | --- | --- | --- | --- | --- | --- | --- | --- | --- | --- | --- | --- | --- | --- | --- | --- | --- | --- | --- | --- | --- | --- | --- | --- | --- | --- | --- |
|  | Hazard ratio  (95% CI) | p-value | p-value of comparison RWD to RCT | |
| RWD approach – observational data adjusted based on propensity scores | | |  | |
| *PS matching – caliper 0* |  |  |  | |
| Univariate survival model | 0.93 (0.49;1.75) | 0.82 | 0.57 | |
| *PS matching – caliper 0.2*sd logit propensity* | |  |  | |
| Univariate survival model | 1.11 (0.65;1.88) | 0.71 | 0.35 | |
| *PS inverse weighting* | |  |  | |
| Univariate survival model | 0.83 (0.23;2.96) | 0.93 | 0.91 | |
|  |  |  |  | |
| *PS stratification* |  |  |  | |
| Univariate survival model | 1.45 (0.55;2.34) | 0.72 | 0.24 | |
| Abbreviations:, , NA = not applicable; RCT = randomized clinical trial; RWD = real world data; PS = propensity score; sd = standard deviation. | | | |  |

**Appendix Figure 1.** Kaplan Meier curves for the studies included in the comparison fluoropyrimidine monotherapy (a) versus control (b) and for the comparison fluoropyrimidine combined with oxaliplatin (c) versus fluoropyrimidine monotherapy (d).

1. (b)

(c) (d)

**References**

1. Ishak KJ, Kreif N, Benedict A, Muszbek N. Overview of parametric survival analysis for health-economic applications. *Pharmacoeconomics* 2013;**31**: 663-75.

2. Latimer NR. Survival analysis for economic evaluations alongside clinical trials—extrapolation with patient-level data: inconsistencies, limitations, and a practical guide. *Medical Decision Making* 2013;**33**: 743-54.

3. Jackson CH. flexsurv: a platform for parametric survival modeling in R. *Journal of Statistical Software* 2016;**70**: 1-33.

4. Therneau TM, Lumley T. Package ‘survival’. *R Top Doc* 2015;**128**.

5. Figueredo A, Coombes ME, Mukherjee S. Adjuvant therapy for completely resected stage II colon cancer, 2008.

6. van Erning FN, Janssen-Heijnen ML, Creemers GJ, Pruijt HF, Maas HA, Lemmens VE. Deciding on adjuvant chemotherapy for elderly patients with stage III colon cancer: a qualitative insight into the perspectives of surgeons and medical oncologists. *Journal of geriatric oncology* 2015;**6**: 219-24.

7. Bang H, Robins JM. Doubly robust estimation in missing data and causal inference models. *Biometrics* 2005;**61**: 962-73.

8. Funk MJ, Westreich D, Wiesen C, Stürmer T, Brookhart MA, Davidian M. Doubly robust estimation of causal effects. *American journal of epidemiology* 2011;**173**: 761-7.

9. Rosenbaum PR, Rubin DB. Reducing bias in observational studies using subclassification on the propensity score. *Journal of the American statistical Association* 1984;**79**: 516-24.

10. Rosenbaum PR, Rubin DB. Constructing a control group using multivariate matched sampling methods that incorporate the propensity score. *The American Statistician* 1985;**39**: 33-8.

11. Austin PC. Optimal caliper widths for propensity-score matching when estimating differences in means and differences in proportions in observational studies. *Pharmaceutical statistics* 2011;**10**: 150-61.

12. Therneau TM, Therneau MTM. Package ‘coxme’. *Mixed Effects Cox Models R package version* 2018;**2**.

13. Lunceford JK, Davidian M. Stratification and weighting via the propensity score in estimation of causal treatment effects: a comparative study. *Statistics in medicine* 2004;**23**: 2937-60.

14. Schafer JL, Kang J. Average causal effects from nonrandomized studies: a practical guide and simulated example. *Psychological methods* 2008;**13**: 279.

15. Austin PC, Stuart EA. Moving towards best practice when using inverse probability of treatment weighting (IPTW) using the propensity score to estimate causal treatment effects in observational studies. *Statistics in medicine* 2015;**34**: 3661-79.

16. Rosenbaum PR, Rubin DB. The central role of the propensity score in observational studies for causal effects. *Biometrika* 1983;**70**: 41-55.

17. Austin PC. The performance of different propensity score methods for estimating marginal hazard ratios. *Statistics in medicine* 2013;**32**: 2837-49.
