# Supplementary material for: Estimating adjuvant treatment effects in Stage II colon cancer: Comparing the synthesis of randomized clinical trial data to real‐world data
Source: Int J Cancer. 2019 Aug 31;146(11):2968–78. doi: 10.1002/ijc.32629 (PMC7187209; doi:10.1002/ijc.32629)
Supplement: Supplementary file 2 — Appendix S2: Supplementary Information [file IJC-146-2968-s002.pdf]

| Subpopulation 1; comparison between fluoropyrimidine monotherapy and control | | | | |
|---|---|---|---|---|
| | Control group | | | |
| | N | | Number of events | |
| | reported data | generated data* | reported data | generated data* |
| IMPACT | 509 | 513 | *NR* | 144 |
| QUASAR | 1480 | 1481 | 289 | 300 |
| Schippinger et al. | 248 | 253 | *NR* | 49 |
| | Fluoropyrimidine group | | | |
| IMPACT | 507 | 507 | *NR* | 122 |
| QUASAR | 1483 | 1478 | 234 | 241 |
| Schippinger et al. | 252 | 257 | *NR* | 36 |
| Subpopulation 2: comparison between FOLFOX and fluoropyrimidine monotherapy | | | | |
| | Fluoropyrimidine group | | | |
| | N | | Number of events | |
| | reported data | generated data* | reported data | generated data* |
| MOSAIC | 448 | 441 | *NR* | 87 |
| NSABP C07 | 349 | 347 | 94 | 88 |
| | Fluoropyrimidine combined with oxaliplatin | | | |
| MOSAIC | 451 | 450 | *NR* | 82 |
| NSABP C07 | 346 | 349 | 87 | 84 |

Abbreviations; NR = not reported

* Based on the method described by Hoyle and Henley (2011)

| | Reported HR (95%CI) | HR in generated data** (95%CI) |
|---|---|---|
| IMPACT | 0.83 (0.72;1.07)* | 0.83 (0.65;1.05) |
| QUASAR | *NR* | 0.77 (0.65;0.92) |
| Schippinger et al. | 0.69 (0.45;1.06) | 0.68 (0.44;1.05) |
| MOSAIC | 0.84 (0.62;1.14) | 0.90 (0.66;1.20) |
| NSABP C07 | 0.94 (0.70;1.26) | 0.95 (0.80;1.45) |

Abbreviations; NR = not reported, CI = confidence interval

*For IMPACT, a 90% CI was reported

** Based on the method described by Hoyle and Henley (2011)

Generated patient level data* based on Figure 1 from the IMPACT trial (1999)

* Based on the method described by Hoyle and Henley (2011)

| Control | | |
|---|---|---|
| ID | Follow_up | Event |
| 1 | 0.125 | 1 |
| 2 | 0.125 | 1 |
| 3 | 0.125 | 1 |
| 4 | 0.125 | 1 |
| 5 | 0.125 | 1 |
| 6 | 0.125 | 1 |
| 7 | 0.125 | 1 |
| 8 | 0.125 | 1 |
| 9 | 0.125 | 1 |
| 10 | 0.375 | 1 |
| 11 | 0.375 | 1 |
| 12 | 0.375 | 1 |
| 13 | 0.375 | 1 |
| 14 | 0.375 | 1 |
| 15 | 0.375 | 1 |
| 16 | 0.375 | 1 |
| 17 | 0.375 | 1 |
| 18 | 0.375 | 1 |
| 19 | 0.375 | 1 |
| 20 | 0.375 | 1 |
| 21 | 0.375 | 1 |
| 22 | 0.375 | 1 |
| 23 | 0.375 | 1 |
| 24 | 0.375 | 1 |
| 25 | 0.625 | 1 |
| 26 | 0.625 | 1 |
| 27 | 0.625 | 1 |
| 28 | 0.625 | 1 |
| 29 | 0.625 | 1 |
| 30 | 0.625 | 1 |
| 31 | 0.625 | 1 |
| 32 | 0.625 | 1 |
| 33 | 0.625 | 1 |
| 34 | 0.625 | 1 |
| 35 | 0.625 | 1 |
| 36 | 0.625 | 1 |
| 37 | 0.625 | 1 |
| 38 | 0.625 | 1 |
| 39 | 0.625 | 1 |
| 40 | 0.875 | 1 |
| 41 | 0.875 | 1 |
| 42 | 0.875 | 1 |
| 43 | 0.875 | 1 |
| 44 | 0.875 | 1 |
| 45 | 0.875 | 1 |
| 46 | 0.875 | 1 |
| 47 | 0.875 | 1 |
| 48 | 0.875 | 1 |
| 49 | 0.875 | 1 |
| 50 | 0.875 | 1 |
| 51 | 0.875 | 1 |
| 52 | 0.875 | 1 |
| 53 | 1.125 | 1 |
| 54 | 1.125 | 1 |
| 55 | 1.125 | 1 |
| 56 | 1.125 | 1 |
| 57 | 1.125 | 1 |
| 58 | 1.125 | 1 |
| 59 | 1.125 | 1 |
| 60 | 1.125 | 1 |
| 61 | 1.125 | 1 |
| 62 | 1.125 | 1 |
| 63 | 1.375 | 1 |
| 64 | 1.375 | 1 |
| 65 | 1.375 | 1 |
| 66 | 1.375 | 1 |
| 67 | 1.375 | 1 |
| 68 | 1.375 | 1 |
| 69 | 1.375 | 1 |
| 70 | 1.375 | 1 |
| 71 | 1.625 | 1 |
| 72 | 1.625 | 1 |
| 73 | 1.625 | 1 |
| 74 | 1.625 | 1 |
| 75 | 1.625 | 1 |
| 76 | 1.625 | 1 |
| 77 | 1.625 | 1 |
| 78 | 1.625 | 1 |
| 79 | 1.625 | 1 |
| 80 | 1.875 | 1 |
| 81 | 1.875 | 1 |
| 82 | 1.875 | 1 |
| 83 | 1.875 | 1 |
| 84 | 1.875 | 1 |
| 85 | 1.875 | 1 |
| 86 | 1.875 | 1 |
| 87 | 1.875 | 1 |
| 88 | 2.125 | 1 |

| Fluoropyrimidine | | |
|---|---|---|
| ID | Follow_up | Event |
| 1 | 0.125 | 1 |
| 2 | 0.125 | 1 |
| 3 | 0.125 | 1 |
| 4 | 0.125 | 1 |
| 5 | 0.125 | 1 |
| 6 | 0.125 | 1 |
| 7 | 0.375 | 1 |
| 8 | 0.375 | 1 |
| 9 | 0.375 | 1 |
| 10 | 0.375 | 1 |
| 11 | 0.625 | 1 |
| 12 | 0.625 | 1 |
| 13 | 0.625 | 1 |
| 14 | 0.625 | 1 |
| 15 | 0.625 | 1 |
| 16 | 0.625 | 1 |
| 17 | 0.625 | 1 |
| 18 | 0.625 | 1 |
| 19 | 0.625 | 1 |
| 20 | 0.875 | 1 |
| 21 | 0.875 | 1 |
| 22 | 0.875 | 1 |
| 23 | 0.875 | 1 |
| 24 | 0.875 | 1 |
| 25 | 0.875 | 1 |
| 26 | 0.875 | 1 |
| 27 | 0.875 | 1 |
| 28 | 0.875 | 1 |
| 29 | 0.875 | 1 |
| 30 | 0.875 | 1 |
| 31 | 0.875 | 1 |
| 32 | 0.875 | 1 |
| 33 | 0.875 | 1 |
| 34 | 0.875 | 1 |
| 35 | 0.875 | 1 |
| 36 | 0.875 | 1 |
| 37 | 1.125 | 1 |
| 38 | 1.125 | 1 |
| 39 | 1.125 | 1 |
| 40 | 1.125 | 1 |
| 41 | 1.125 | 1 |
| 42 | 1.125 | 1 |
| 43 | 1.125 | 1 |
| 44 | 1.125 | 1 |
| 45 | 1.125 | 1 |
| 46 | 1.125 | 1 |
| 47 | 1.125 | 1 |
| 48 | 1.125 | 1 |
| 49 | 1.125 | 1 |
| 50 | 1.375 | 1 |
| 51 | 1.375 | 1 |
| 52 | 1.375 | 1 |
| 53 | 1.375 | 1 |
| 54 | 1.375 | 1 |
| 55 | 1.375 | 1 |
| 56 | 1.375 | 1 |
| 57 | 1.375 | 1 |
| 58 | 1.625 | 1 |
| 59 | 1.625 | 1 |
| 60 | 1.625 | 1 |
| 61 | 1.625 | 1 |
| 62 | 1.625 | 1 |
| 63 | 1.625 | 1 |
| 64 | 1.625 | 1 |
| 65 | 1.625 | 1 |
| 66 | 1.625 | 1 |
| 67 | 1.875 | 1 |
| 68 | 1.875 | 1 |
| 69 | 1.875 | 1 |
| 70 | 1.875 | 1 |
| 71 | 1.875 | 1 |
| 72 | 1.875 | 1 |
| 73 | 2.125 | 1 |
| 74 | 2.125 | 1 |
| 75 | 2.125 | 1 |
| 76 | 2.125 | 1 |
| 77 | 2.125 | 1 |
| 78 | 2.125 | 1 |
| 79 | 2.125 | 1 |
| 80 | 2.125 | 1 |
| 81 | 2.125 | 1 |
| 82 | 2.125 | 1 |
| 83 | 2.125 | 1 |
| 84 | 2.375 | 1 |
| 85 | 2.375 | 1 |
| 86 | 2.625 | 1 |
| 87 | 2.625 | 1 |
| 88 | 2.625 | 1 |

| | |
|---|---|
| ID | Patient ID |
| Follow_up | Follow up time in years |
| Event | 0 = no recurrence |
| | 1 = recurrence |

| | | |
|---|---|---|
| 89 | 2.125 | 1 |
| 90 | 2.125 | 1 |
| 91 | 2.125 | 1 |
| 92 | 2.125 | 1 |
| 93 | 2.125 | 1 |
| 94 | 2.125 | 1 |
| 95 | 2.125 | 1 |
| 96 | 2.125 | 1 |
| 97 | 2.125 | 1 |
| 98 | 2.125 | 1 |
| 99 | 2.375 | 1 |
| 100 | 2.375 | 1 |
| 101 | 2.375 | 1 |
| 102 | 2.375 | 1 |
| 103 | 2.625 | 1 |
| 104 | 2.625 | 1 |
| 105 | 2.625 | 1 |
| 106 | 2.625 | 1 |
| 107 | 2.875 | 1 |
| 108 | 3.125 | 1 |
| 109 | 3.125 | 1 |
| 110 | 3.125 | 1 |
| 111 | 3.125 | 1 |
| 112 | 3.375 | 1 |
| 113 | 3.375 | 1 |
| 114 | 3.625 | 1 |
| 115 | 3.875 | 1 |
| 116 | 3.875 | 1 |
| 117 | 3.875 | 1 |
| 118 | 4.125 | 1 |
| 119 | 4.125 | 1 |
| 120 | 4.125 | 1 |
| 121 | 4.375 | 1 |
| 122 | 4.625 | 1 |
| 123 | 4.625 | 1 |
| 124 | 4.625 | 1 |
| 125 | 4.625 | 1 |
| 126 | 4.875 | 1 |
| 127 | 4.875 | 1 |
| 128 | 4.875 | 1 |
| 129 | 5.125 | 1 |
| 130 | 5.375 | 1 |
| 131 | 5.375 | 1 |
| 132 | 5.375 | 1 |
| 133 | 5.375 | 1 |
| 134 | 5.375 | 1 |
| 135 | 5.875 | 1 |
| 136 | 5.875 | 1 |
| 137 | 6.125 | 1 |
| 138 | 6.125 | 1 |
| 139 | 6.625 | 1 |
| 140 | 6.875 | 1 |
| 141 | 7.125 | 1 |
| 142 | 7.375 | 1 |
| 143 | 7.625 | 1 |
| 144 | 7.625 | 1 |
| 145 | 0.125 | 0 |
| 146 | 0.375 | 0 |
| 147 | 0.625 | 0 |
| 148 | 0.875 | 0 |
| 149 | 1.125 | 0 |
| 150 | 1.375 | 0 |
| 151 | 1.625 | 0 |
| 152 | 1.875 | 0 |
| 153 | 2.125 | 0 |
| 154 | 2.125 | 0 |
| 155 | 2.125 | 0 |
| 156 | 2.375 | 0 |
| 157 | 2.375 | 0 |
| 158 | 2.375 | 0 |
| 159 | 2.625 | 0 |
| 160 | 2.625 | 0 |
| 161 | 2.625 | 0 |
| 162 | 2.875 | 0 |
| 163 | 2.875 | 0 |
| 164 | 2.875 | 0 |
| 165 | 3.125 | 0 |
| 166 | 3.125 | 0 |
| 167 | 3.125 | 0 |
| 168 | 3.125 | 0 |
| 169 | 3.125 | 0 |
| 170 | 3.125 | 0 |
| 171 | 3.375 | 0 |
| 172 | 3.375 | 0 |
| 173 | 3.375 | 0 |
| 174 | 3.375 | 0 |
| 175 | 3.375 | 0 |
| 176 | 3.375 | 0 |
| 177 | 3.625 | 0 |
| 178 | 3.625 | 0 |
| 179 | 3.625 | 0 |
| 180 | 3.625 | 0 |
| 181 | 3.625 | 0 |

| | | |
|---|---|---|
| 89 | 2.625 | 1 |
| 90 | 2.625 | 1 |
| 91 | 2.875 | 1 |
| 92 | 2.875 | 1 |
| 93 | 2.875 | 1 |
| 94 | 2.875 | 1 |
| 95 | 3.125 | 1 |
| 96 | 3.125 | 1 |
| 97 | 3.125 | 1 |
| 98 | 3.375 | 1 |
| 99 | 3.375 | 1 |
| 100 | 3.625 | 1 |
| 101 | 3.625 | 1 |
| 102 | 3.625 | 1 |
| 103 | 3.875 | 1 |
| 104 | 3.875 | 1 |
| 105 | 3.875 | 1 |
| 106 | 4.125 | 1 |
| 107 | 4.125 | 1 |
| 108 | 4.375 | 1 |
| 109 | 4.375 | 1 |
| 110 | 4.375 | 1 |
| 111 | 4.625 | 1 |
| 112 | 4.625 | 1 |
| 113 | 4.625 | 1 |
| 114 | 4.875 | 1 |
| 115 | 5.125 | 1 |
| 116 | 5.125 | 1 |
| 117 | 5.125 | 1 |
| 118 | 5.375 | 1 |
| 119 | 5.375 | 1 |
| 120 | 5.875 | 1 |
| 121 | 6.875 | 1 |
| 122 | 7.875 | 1 |
| 123 | 0.125 | 0 |
| 124 | 0.375 | 0 |
| 125 | 0.625 | 0 |
| 126 | 0.875 | 0 |
| 127 | 1.125 | 0 |
| 128 | 1.125 | 0 |
| 129 | 1.375 | 0 |
| 130 | 1.375 | 0 |
| 131 | 1.625 | 0 |
| 132 | 1.625 | 0 |
| 133 | 1.875 | 0 |
| 134 | 1.875 | 0 |
| 135 | 2.125 | 0 |
| 136 | 2.125 | 0 |
| 137 | 2.375 | 0 |
| 138 | 2.375 | 0 |
| 139 | 2.625 | 0 |
| 140 | 2.625 | 0 |
| 141 | 2.875 | 0 |
| 142 | 2.875 | 0 |
| 143 | 3.125 | 0 |
| 144 | 3.125 | 0 |
| 145 | 3.125 | 0 |
| 146 | 3.125 | 0 |
| 147 | 3.125 | 0 |
| 148 | 3.125 | 0 |
| 149 | 3.375 | 0 |
| 150 | 3.375 | 0 |
| 151 | 3.375 | 0 |
| 152 | 3.375 | 0 |
| 153 | 3.375 | 0 |
| 154 | 3.375 | 0 |
| 155 | 3.625 | 0 |
| 156 | 3.625 | 0 |
| 157 | 3.625 | 0 |
| 158 | 3.625 | 0 |
| 159 | 3.625 | 0 |
| 160 | 3.625 | 0 |
| 161 | 3.875 | 0 |
| 162 | 3.875 | 0 |
| 163 | 3.875 | 0 |
| 164 | 3.875 | 0 |
| 165 | 3.875 | 0 |
| 166 | 3.875 | 0 |
| 167 | 4.125 | 0 |
| 168 | 4.125 | 0 |
| 169 | 4.125 | 0 |
| 170 | 4.125 | 0 |
| 171 | 4.125 | 0 |
| 172 | 4.125 | 0 |
| 173 | 4.125 | 0 |
| 174 | 4.125 | 0 |
| 175 | 4.125 | 0 |
| 176 | 4.125 | 0 |
| 177 | 4.125 | 0 |
| 178 | 4.125 | 0 |
| 179 | 4.125 | 0 |
| 180 | 4.125 | 0 |
| 181 | 4.125 | 0 |

| | | |
|---|---|---|
| 182 | 3.625 | 0 |
| 183 | 3.875 | 0 |
| 184 | 3.875 | 0 |
| 185 | 3.875 | 0 |
| 186 | 3.875 | 0 |
| 187 | 3.875 | 0 |
| 188 | 3.875 | 0 |
| 189 | 4.125 | 0 |
| 190 | 4.125 | 0 |
| 191 | 4.125 | 0 |
| 192 | 4.125 | 0 |
| 193 | 4.125 | 0 |
| 194 | 4.125 | 0 |
| 195 | 4.125 | 0 |
| 196 | 4.125 | 0 |
| 197 | 4.125 | 0 |
| 198 | 4.125 | 0 |
| 199 | 4.125 | 0 |
| 200 | 4.125 | 0 |
| 201 | 4.125 | 0 |
| 202 | 4.125 | 0 |
| 203 | 4.125 | 0 |
| 204 | 4.125 | 0 |
| 205 | 4.125 | 0 |
| 206 | 4.125 | 0 |
| 207 | 4.125 | 0 |
| 208 | 4.125 | 0 |
| 209 | 4.125 | 0 |
| 210 | 4.125 | 0 |
| 211 | 4.125 | 0 |
| 212 | 4.125 | 0 |
| 213 | 4.125 | 0 |
| 214 | 4.375 | 0 |
| 215 | 4.375 | 0 |
| 216 | 4.375 | 0 |
| 217 | 4.375 | 0 |
| 218 | 4.375 | 0 |
| 219 | 4.375 | 0 |
| 220 | 4.375 | 0 |
| 221 | 4.375 | 0 |
| 222 | 4.375 | 0 |
| 223 | 4.375 | 0 |
| 224 | 4.375 | 0 |
| 225 | 4.375 | 0 |
| 226 | 4.375 | 0 |
| 227 | 4.375 | 0 |
| 228 | 4.375 | 0 |
| 229 | 4.375 | 0 |
| 230 | 4.375 | 0 |
| 231 | 4.375 | 0 |
| 232 | 4.375 | 0 |
| 233 | 4.375 | 0 |
| 234 | 4.375 | 0 |
| 235 | 4.375 | 0 |
| 236 | 4.375 | 0 |
| 237 | 4.375 | 0 |
| 238 | 4.375 | 0 |
| 239 | 4.625 | 0 |
| 240 | 4.625 | 0 |
| 241 | 4.625 | 0 |
| 242 | 4.625 | 0 |
| 243 | 4.625 | 0 |
| 244 | 4.625 | 0 |
| 245 | 4.625 | 0 |
| 246 | 4.625 | 0 |
| 247 | 4.625 | 0 |
| 248 | 4.625 | 0 |
| 249 | 4.625 | 0 |
| 250 | 4.625 | 0 |
| 251 | 4.625 | 0 |
| 252 | 4.625 | 0 |
| 253 | 4.625 | 0 |
| 254 | 4.625 | 0 |
| 255 | 4.625 | 0 |
| 256 | 4.625 | 0 |
| 257 | 4.625 | 0 |
| 258 | 4.625 | 0 |
| 259 | 4.625 | 0 |
| 260 | 4.625 | 0 |
| 261 | 4.625 | 0 |
| 262 | 4.625 | 0 |
| 263 | 4.625 | 0 |
| 264 | 4.875 | 0 |
| 265 | 4.875 | 0 |
| 266 | 4.875 | 0 |
| 267 | 4.875 | 0 |
| 268 | 4.875 | 0 |
| 269 | 4.875 | 0 |
| 270 | 4.875 | 0 |
| 271 | 4.875 | 0 |
| 272 | 4.875 | 0 |
| 273 | 4.875 | 0 |
| 274 | 4.875 | 0 |

| | | |
|---|---|---|
| 182 | 4.125 | 0 |
| 183 | 4.125 | 0 |
| 184 | 4.125 | 0 |
| 185 | 4.125 | 0 |
| 186 | 4.125 | 0 |
| 187 | 4.125 | 0 |
| 188 | 4.375 | 0 |
| 189 | 4.375 | 0 |
| 190 | 4.375 | 0 |
| 191 | 4.375 | 0 |
| 192 | 4.375 | 0 |
| 193 | 4.375 | 0 |
| 194 | 4.375 | 0 |
| 195 | 4.375 | 0 |
| 196 | 4.375 | 0 |
| 197 | 4.375 | 0 |
| 198 | 4.375 | 0 |
| 199 | 4.375 | 0 |
| 200 | 4.375 | 0 |
| 201 | 4.375 | 0 |
| 202 | 4.375 | 0 |
| 203 | 4.375 | 0 |
| 204 | 4.375 | 0 |
| 205 | 4.375 | 0 |
| 206 | 4.375 | 0 |
| 207 | 4.375 | 0 |
| 208 | 4.375 | 0 |
| 209 | 4.625 | 0 |
| 210 | 4.625 | 0 |
| 211 | 4.625 | 0 |
| 212 | 4.625 | 0 |
| 213 | 4.625 | 0 |
| 214 | 4.625 | 0 |
| 215 | 4.625 | 0 |
| 216 | 4.625 | 0 |
| 217 | 4.625 | 0 |
| 218 | 4.625 | 0 |
| 219 | 4.625 | 0 |
| 220 | 4.625 | 0 |
| 221 | 4.625 | 0 |
| 222 | 4.625 | 0 |
| 223 | 4.625 | 0 |
| 224 | 4.625 | 0 |
| 225 | 4.625 | 0 |
| 226 | 4.625 | 0 |
| 227 | 4.625 | 0 |
| 228 | 4.625 | 0 |
| 229 | 4.625 | 0 |
| 230 | 4.875 | 0 |
| 231 | 4.875 | 0 |
| 232 | 4.875 | 0 |
| 233 | 4.875 | 0 |
| 234 | 4.875 | 0 |
| 235 | 4.875 | 0 |
| 236 | 4.875 | 0 |
| 237 | 4.875 | 0 |
| 238 | 4.875 | 0 |
| 239 | 4.875 | 0 |
| 240 | 4.875 | 0 |
| 241 | 4.875 | 0 |
| 242 | 4.875 | 0 |
| 243 | 4.875 | 0 |
| 244 | 4.875 | 0 |
| 245 | 4.875 | 0 |
| 246 | 4.875 | 0 |
| 247 | 4.875 | 0 |
| 248 | 4.875 | 0 |
| 249 | 4.875 | 0 |
| 250 | 4.875 | 0 |
| 251 | 5.125 | 0 |
| 252 | 5.125 | 0 |
| 253 | 5.125 | 0 |
| 254 | 5.125 | 0 |
| 255 | 5.125 | 0 |
| 256 | 5.125 | 0 |
| 257 | 5.125 | 0 |
| 258 | 5.125 | 0 |
| 259 | 5.125 | 0 |
| 260 | 5.125 | 0 |
| 261 | 5.125 | 0 |
| 262 | 5.125 | 0 |
| 263 | 5.125 | 0 |
| 264 | 5.125 | 0 |
| 265 | 5.125 | 0 |
| 266 | 5.125 | 0 |
| 267 | 5.125 | 0 |
| 268 | 5.125 | 0 |
| 269 | 5.125 | 0 |
| 270 | 5.125 | 0 |
| 271 | 5.125 | 0 |
| 272 | 5.125 | 0 |
| 273 | 5.375 | 0 |
| 274 | 5.375 | 0 |

| | | |
|---|---|---|
| 275 | 4.875 | 0 |
| 276 | 4.875 | 0 |
| 277 | 4.875 | 0 |
| 278 | 4.875 | 0 |
| 279 | 4.875 | 0 |
| 280 | 4.875 | 0 |
| 281 | 4.875 | 0 |
| 282 | 4.875 | 0 |
| 283 | 4.875 | 0 |
| 284 | 4.875 | 0 |
| 285 | 4.875 | 0 |
| 286 | 4.875 | 0 |
| 287 | 4.875 | 0 |
| 288 | 4.875 | 0 |
| 289 | 5.125 | 0 |
| 290 | 5.125 | 0 |
| 291 | 5.125 | 0 |
| 292 | 5.125 | 0 |
| 293 | 5.125 | 0 |
| 294 | 5.125 | 0 |
| 295 | 5.125 | 0 |
| 296 | 5.125 | 0 |
| 297 | 5.125 | 0 |
| 298 | 5.125 | 0 |
| 299 | 5.125 | 0 |
| 300 | 5.125 | 0 |
| 301 | 5.125 | 0 |
| 302 | 5.125 | 0 |
| 303 | 5.125 | 0 |
| 304 | 5.125 | 0 |
| 305 | 5.125 | 0 |
| 306 | 5.125 | 0 |
| 307 | 5.125 | 0 |
| 308 | 5.375 | 0 |
| 309 | 5.375 | 0 |
| 310 | 5.375 | 0 |
| 311 | 5.375 | 0 |
| 312 | 5.375 | 0 |
| 313 | 5.375 | 0 |
| 314 | 5.375 | 0 |
| 315 | 5.375 | 0 |
| 316 | 5.375 | 0 |
| 317 | 5.375 | 0 |
| 318 | 5.375 | 0 |
| 319 | 5.375 | 0 |
| 320 | 5.375 | 0 |
| 321 | 5.375 | 0 |
| 322 | 5.375 | 0 |
| 323 | 5.375 | 0 |
| 324 | 5.375 | 0 |
| 325 | 5.375 | 0 |
| 326 | 5.375 | 0 |
| 327 | 5.625 | 0 |
| 328 | 5.625 | 0 |
| 329 | 5.625 | 0 |
| 330 | 5.625 | 0 |
| 331 | 5.625 | 0 |
| 332 | 5.625 | 0 |
| 333 | 5.625 | 0 |
| 334 | 5.625 | 0 |
| 335 | 5.625 | 0 |
| 336 | 5.625 | 0 |
| 337 | 5.625 | 0 |
| 338 | 5.625 | 0 |
| 339 | 5.625 | 0 |
| 340 | 5.625 | 0 |
| 341 | 5.625 | 0 |
| 342 | 5.625 | 0 |
| 343 | 5.625 | 0 |
| 344 | 5.625 | 0 |
| 345 | 5.625 | 0 |
| 346 | 5.875 | 0 |
| 347 | 5.875 | 0 |
| 348 | 5.875 | 0 |
| 349 | 5.875 | 0 |
| 350 | 5.875 | 0 |
| 351 | 5.875 | 0 |
| 352 | 5.875 | 0 |
| 353 | 5.875 | 0 |
| 354 | 5.875 | 0 |
| 355 | 5.875 | 0 |
| 356 | 5.875 | 0 |
| 357 | 5.875 | 0 |
| 358 | 5.875 | 0 |
| 359 | 5.875 | 0 |
| 360 | 5.875 | 0 |
| 361 | 5.875 | 0 |
| 362 | 5.875 | 0 |
| 363 | 5.875 | 0 |
| 364 | 5.875 | 0 |
| 365 | 6.125 | 0 |
| 366 | 6.125 | 0 |
| 367 | 6.125 | 0 |

| | | |
|---|---|---|
| 275 | 5.375 | 0 |
| 276 | 5.375 | 0 |
| 277 | 5.375 | 0 |
| 278 | 5.375 | 0 |
| 279 | 5.375 | 0 |
| 280 | 5.375 | 0 |
| 281 | 5.375 | 0 |
| 282 | 5.375 | 0 |
| 283 | 5.375 | 0 |
| 284 | 5.375 | 0 |
| 285 | 5.375 | 0 |
| 286 | 5.375 | 0 |
| 287 | 5.375 | 0 |
| 288 | 5.375 | 0 |
| 289 | 5.375 | 0 |
| 290 | 5.375 | 0 |
| 291 | 5.375 | 0 |
| 292 | 5.375 | 0 |
| 293 | 5.375 | 0 |
| 294 | 5.375 | 0 |
| 295 | 5.625 | 0 |
| 296 | 5.625 | 0 |
| 297 | 5.625 | 0 |
| 298 | 5.625 | 0 |
| 299 | 5.625 | 0 |
| 300 | 5.625 | 0 |
| 301 | 5.625 | 0 |
| 302 | 5.625 | 0 |
| 303 | 5.625 | 0 |
| 304 | 5.625 | 0 |
| 305 | 5.625 | 0 |
| 306 | 5.625 | 0 |
| 307 | 5.625 | 0 |
| 308 | 5.625 | 0 |
| 309 | 5.625 | 0 |
| 310 | 5.625 | 0 |
| 311 | 5.625 | 0 |
| 312 | 5.625 | 0 |
| 313 | 5.625 | 0 |
| 314 | 5.625 | 0 |
| 315 | 5.625 | 0 |
| 316 | 5.625 | 0 |
| 317 | 5.875 | 0 |
| 318 | 5.875 | 0 |
| 319 | 5.875 | 0 |
| 320 | 5.875 | 0 |
| 321 | 5.875 | 0 |
| 322 | 5.875 | 0 |
| 323 | 5.875 | 0 |
| 324 | 5.875 | 0 |
| 325 | 5.875 | 0 |
| 326 | 5.875 | 0 |
| 327 | 5.875 | 0 |
| 328 | 5.875 | 0 |
| 329 | 5.875 | 0 |
| 330 | 5.875 | 0 |
| 331 | 5.875 | 0 |
| 332 | 5.875 | 0 |
| 333 | 5.875 | 0 |
| 334 | 5.875 | 0 |
| 335 | 5.875 | 0 |
| 336 | 5.875 | 0 |
| 337 | 5.875 | 0 |
| 338 | 5.875 | 0 |
| 339 | 6.125 | 0 |
| 340 | 6.125 | 0 |
| 341 | 6.125 | 0 |
| 342 | 6.125 | 0 |
| 343 | 6.125 | 0 |
| 344 | 6.125 | 0 |
| 345 | 6.125 | 0 |
| 346 | 6.125 | 0 |
| 347 | 6.125 | 0 |
| 348 | 6.125 | 0 |
| 349 | 6.125 | 0 |
| 350 | 6.125 | 0 |
| 351 | 6.125 | 0 |
| 352 | 6.125 | 0 |
| 353 | 6.125 | 0 |
| 354 | 6.125 | 0 |
| 355 | 6.125 | 0 |
| 356 | 6.125 | 0 |
| 357 | 6.125 | 0 |
| 358 | 6.125 | 0 |
| 359 | 6.125 | 0 |
| 360 | 6.125 | 0 |
| 361 | 6.125 | 0 |
| 362 | 6.375 | 0 |
| 363 | 6.375 | 0 |
| 364 | 6.375 | 0 |
| 365 | 6.375 | 0 |
| 366 | 6.375 | 0 |
| 367 | 6.375 | 0 |

| | | |
|---|---|---|
| 368 | 6.125 | 0 |
| 369 | 6.125 | 0 |
| 370 | 6.125 | 0 |
| 371 | 6.125 | 0 |
| 372 | 6.125 | 0 |
| 373 | 6.125 | 0 |
| 374 | 6.125 | 0 |
| 375 | 6.125 | 0 |
| 376 | 6.125 | 0 |
| 377 | 6.125 | 0 |
| 378 | 6.125 | 0 |
| 379 | 6.125 | 0 |
| 380 | 6.125 | 0 |
| 381 | 6.125 | 0 |
| 382 | 6.125 | 0 |
| 383 | 6.375 | 0 |
| 384 | 6.375 | 0 |
| 385 | 6.375 | 0 |
| 386 | 6.375 | 0 |
| 387 | 6.375 | 0 |
| 388 | 6.375 | 0 |
| 389 | 6.375 | 0 |
| 390 | 6.375 | 0 |
| 391 | 6.375 | 0 |
| 392 | 6.375 | 0 |
| 393 | 6.375 | 0 |
| 394 | 6.375 | 0 |
| 395 | 6.375 | 0 |
| 396 | 6.375 | 0 |
| 397 | 6.375 | 0 |
| 398 | 6.375 | 0 |
| 399 | 6.375 | 0 |
| 400 | 6.375 | 0 |
| 401 | 6.625 | 0 |
| 402 | 6.625 | 0 |
| 403 | 6.625 | 0 |
| 404 | 6.625 | 0 |
| 405 | 6.625 | 0 |
| 406 | 6.625 | 0 |
| 407 | 6.625 | 0 |
| 408 | 6.625 | 0 |
| 409 | 6.625 | 0 |
| 410 | 6.625 | 0 |
| 411 | 6.625 | 0 |
| 412 | 6.625 | 0 |
| 413 | 6.625 | 0 |
| 414 | 6.625 | 0 |
| 415 | 6.625 | 0 |
| 416 | 6.625 | 0 |
| 417 | 6.625 | 0 |
| 418 | 6.625 | 0 |
| 419 | 6.875 | 0 |
| 420 | 6.875 | 0 |
| 421 | 6.875 | 0 |
| 422 | 6.875 | 0 |
| 423 | 6.875 | 0 |
| 424 | 6.875 | 0 |
| 425 | 6.875 | 0 |
| 426 | 6.875 | 0 |
| 427 | 6.875 | 0 |
| 428 | 6.875 | 0 |
| 429 | 6.875 | 0 |
| 430 | 6.875 | 0 |
| 431 | 6.875 | 0 |
| 432 | 6.875 | 0 |
| 433 | 6.875 | 0 |
| 434 | 6.875 | 0 |
| 435 | 6.875 | 0 |
| 436 | 6.875 | 0 |
| 437 | 7.125 | 0 |
| 438 | 7.125 | 0 |
| 439 | 7.125 | 0 |
| 440 | 7.125 | 0 |
| 441 | 7.125 | 0 |
| 442 | 7.125 | 0 |
| 443 | 7.125 | 0 |
| 444 | 7.125 | 0 |
| 445 | 7.375 | 0 |
| 446 | 7.375 | 0 |
| 447 | 7.375 | 0 |
| 448 | 7.375 | 0 |
| 449 | 7.375 | 0 |
| 450 | 7.375 | 0 |
| 451 | 7.375 | 0 |
| 452 | 7.375 | 0 |
| 453 | 7.625 | 0 |
| 454 | 7.625 | 0 |
| 455 | 7.625 | 0 |
| 456 | 7.625 | 0 |
| 457 | 7.625 | 0 |
| 458 | 7.625 | 0 |
| 459 | 7.625 | 0 |
| 460 | 7.625 | 0 |

| | | |
|---|---|---|
| 368 | 6.375 | 0 |
| 369 | 6.375 | 0 |
| 370 | 6.375 | 0 |
| 371 | 6.375 | 0 |
| 372 | 6.375 | 0 |
| 373 | 6.375 | 0 |
| 374 | 6.375 | 0 |
| 375 | 6.375 | 0 |
| 376 | 6.375 | 0 |
| 377 | 6.375 | 0 |
| 378 | 6.375 | 0 |
| 379 | 6.375 | 0 |
| 380 | 6.375 | 0 |
| 381 | 6.375 | 0 |
| 382 | 6.375 | 0 |
| 383 | 6.375 | 0 |
| 384 | 6.375 | 0 |
| 385 | 6.625 | 0 |
| 386 | 6.625 | 0 |
| 387 | 6.625 | 0 |
| 388 | 6.625 | 0 |
| 389 | 6.625 | 0 |
| 390 | 6.625 | 0 |
| 391 | 6.625 | 0 |
| 392 | 6.625 | 0 |
| 393 | 6.625 | 0 |
| 394 | 6.625 | 0 |
| 395 | 6.625 | 0 |
| 396 | 6.625 | 0 |
| 397 | 6.625 | 0 |
| 398 | 6.625 | 0 |
| 399 | 6.625 | 0 |
| 400 | 6.625 | 0 |
| 401 | 6.625 | 0 |
| 402 | 6.625 | 0 |
| 403 | 6.625 | 0 |
| 404 | 6.625 | 0 |
| 405 | 6.625 | 0 |
| 406 | 6.625 | 0 |
| 407 | 6.625 | 0 |
| 408 | 6.875 | 0 |
| 409 | 6.875 | 0 |
| 410 | 6.875 | 0 |
| 411 | 6.875 | 0 |
| 412 | 6.875 | 0 |
| 413 | 6.875 | 0 |
| 414 | 6.875 | 0 |
| 415 | 6.875 | 0 |
| 416 | 6.875 | 0 |
| 417 | 6.875 | 0 |
| 418 | 6.875 | 0 |
| 419 | 6.875 | 0 |
| 420 | 6.875 | 0 |
| 421 | 6.875 | 0 |
| 422 | 6.875 | 0 |
| 423 | 6.875 | 0 |
| 424 | 6.875 | 0 |
| 425 | 6.875 | 0 |
| 426 | 6.875 | 0 |
| 427 | 6.875 | 0 |
| 428 | 6.875 | 0 |
| 429 | 6.875 | 0 |
| 430 | 6.875 | 0 |
| 431 | 7.125 | 0 |
| 432 | 7.125 | 0 |
| 433 | 7.125 | 0 |
| 434 | 7.125 | 0 |
| 435 | 7.125 | 0 |
| 436 | 7.125 | 0 |
| 437 | 7.125 | 0 |
| 438 | 7.125 | 0 |
| 439 | 7.375 | 0 |
| 440 | 7.375 | 0 |
| 441 | 7.375 | 0 |
| 442 | 7.375 | 0 |
| 443 | 7.375 | 0 |
| 444 | 7.375 | 0 |
| 445 | 7.375 | 0 |
| 446 | 7.375 | 0 |
| 447 | 7.625 | 0 |
| 448 | 7.625 | 0 |
| 449 | 7.625 | 0 |
| 450 | 7.625 | 0 |
| 451 | 7.625 | 0 |
| 452 | 7.625 | 0 |
| 453 | 7.625 | 0 |
| 454 | 7.625 | 0 |
| 455 | 7.875 | 0 |
| 456 | 7.875 | 0 |
| 457 | 7.875 | 0 |
| 458 | 7.875 | 0 |
| 459 | 7.875 | 0 |
| 460 | 7.875 | 0 |

| | | |
|---|---|---|
| 461 | 7.875 | 0 |
| 462 | 7.875 | 0 |
| 463 | 7.875 | 0 |
| 464 | 7.875 | 0 |
| 465 | 7.875 | 0 |
| 466 | 7.875 | 0 |
| 467 | 7.875 | 0 |
| 468 | 7.875 | 0 |
| 469 | 7.875 | 0 |
| 470 | 7.875 | 0 |
| 471 | 7.875 | 0 |
| 472 | 7.875 | 0 |
| 473 | 7.875 | 0 |
| 474 | 7.875 | 0 |
| 475 | 7.875 | 0 |
| 476 | 7.875 | 0 |
| 477 | 7.875 | 0 |
| 478 | 7.875 | 0 |
| 479 | 7.875 | 0 |
| 480 | 7.875 | 0 |
| 481 | 7.875 | 0 |
| 482 | 7.875 | 0 |
| 483 | 7.875 | 0 |
| 484 | 7.875 | 0 |
| 485 | 7.875 | 0 |
| 486 | 7.875 | 0 |
| 487 | 7.875 | 0 |
| 488 | 7.875 | 0 |
| 489 | 7.875 | 0 |
| 490 | 7.875 | 0 |
| 491 | 7.875 | 0 |
| 492 | 7.875 | 0 |
| 493 | 7.875 | 0 |
| 494 | 7.875 | 0 |
| 495 | 7.875 | 0 |
| 496 | 7.875 | 0 |
| 497 | 7.875 | 0 |
| 498 | 7.875 | 0 |
| 499 | 7.875 | 0 |
| 500 | 7.875 | 0 |
| 501 | 7.875 | 0 |
| 502 | 7.875 | 0 |
| 503 | 7.875 | 0 |
| 504 | 7.875 | 0 |
| 505 | 7.875 | 0 |
| 506 | 7.875 | 0 |
| 507 | 7.875 | 0 |
| 508 | 7.875 | 0 |
| 509 | 7.875 | 0 |
| 510 | 7.875 | 0 |
| 511 | 7.875 | 0 |
| 512 | 7.875 | 0 |
| 513 | 7.875 | 0 |

| | | |
|---|---|---|
| 461 | 7.875 | 0 |
| 462 | 7.875 | 0 |
| 463 | 7.875 | 0 |
| 464 | 7.875 | 0 |
| 465 | 7.875 | 0 |
| 466 | 7.875 | 0 |
| 467 | 7.875 | 0 |
| 468 | 7.875 | 0 |
| 469 | 7.875 | 0 |
| 470 | 7.875 | 0 |
| 471 | 7.875 | 0 |
| 472 | 7.875 | 0 |
| 473 | 7.875 | 0 |
| 474 | 7.875 | 0 |
| 475 | 7.875 | 0 |
| 476 | 7.875 | 0 |
| 477 | 7.875 | 0 |
| 478 | 7.875 | 0 |
| 479 | 7.875 | 0 |
| 480 | 7.875 | 0 |
| 481 | 7.875 | 0 |
| 482 | 7.875 | 0 |
| 483 | 7.875 | 0 |
| 484 | 7.875 | 0 |
| 485 | 7.875 | 0 |
| 486 | 7.875 | 0 |
| 487 | 7.875 | 0 |
| 488 | 7.875 | 0 |
| 489 | 7.875 | 0 |
| 490 | 7.875 | 0 |
| 491 | 7.875 | 0 |
| 492 | 7.875 | 0 |
| 493 | 7.875 | 0 |
| 494 | 7.875 | 0 |
| 495 | 7.875 | 0 |
| 496 | 7.875 | 0 |
| 497 | 7.875 | 0 |
| 498 | 7.875 | 0 |
| 499 | 7.875 | 0 |
| 500 | 7.875 | 0 |
| 501 | 7.875 | 0 |
| 502 | 7.875 | 0 |
| 503 | 7.875 | 0 |
| 504 | 7.875 | 0 |
| 505 | 7.875 | 0 |
| 506 | 7.875 | 0 |
| 507 | 7.875 | 0 |

Generated patient level data* based on Figure 4a from the QUASAR trial (2007)

* Based on the method described by Hoyle and Henley (2011)

| Control | | | Fluoropyrimidine | | |
|---|---|---|---|---|---|
| ID | Follow_up | Event | ID | Follow_up | Event |
| 1 | 0.125 | 1 | 1 | 0.375 | 1 |
| 2 | 0.125 | 1 | 2 | 0.375 | 1 |
| 3 | 0.125 | 1 | 3 | 0.375 | 1 |
| 4 | 0.125 | 1 | 4 | 0.375 | 1 |
| 5 | 0.125 | 1 | 5 | 0.375 | 1 |
| 6 | 0.125 | 1 | 6 | 0.375 | 1 |
| 7 | 0.125 | 1 | 7 | 0.375 | 1 |
| 8 | 0.125 | 1 | 8 | 0.375 | 1 |
| 9 | 0.125 | 1 | 9 | 0.375 | 1 |
| 10 | 0.125 | 1 | 10 | 0.375 | 1 |
| 11 | 0.125 | 1 | 11 | 0.375 | 1 |
| 12 | 0.375 | 1 | 12 | 0.375 | 1 |
| 13 | 0.375 | 1 | 13 | 0.375 | 1 |
| 14 | 0.375 | 1 | 14 | 0.375 | 1 |
| 15 | 0.375 | 1 | 15 | 0.375 | 1 |
| 16 | 0.375 | 1 | 16 | 0.375 | 1 |
| 17 | 0.375 | 1 | 17 | 0.375 | 1 |
| 18 | 0.375 | 1 | 18 | 0.375 | 1 |
| 19 | 0.375 | 1 | 19 | 0.375 | 1 |
| 20 | 0.375 | 1 | 20 | 0.375 | 1 |
| 21 | 0.375 | 1 | 21 | 0.375 | 1 |
| 22 | 0.375 | 1 | 22 | 0.375 | 1 |
| 23 | 0.375 | 1 | 23 | 0.625 | 1 |
| 24 | 0.375 | 1 | 24 | 0.625 | 1 |
| 25 | 0.375 | 1 | 25 | 0.625 | 1 |
| 26 | 0.375 | 1 | 26 | 0.625 | 1 |
| 27 | 0.375 | 1 | 27 | 0.625 | 1 |
| 28 | 0.625 | 1 | 28 | 0.625 | 1 |
| 29 | 0.625 | 1 | 29 | 0.625 | 1 |
| 30 | 0.625 | 1 | 30 | 0.625 | 1 |
| 31 | 0.625 | 1 | 31 | 0.625 | 1 |
| 32 | 0.625 | 1 | 32 | 0.625 | 1 |
| 33 | 0.625 | 1 | 33 | 0.625 | 1 |
| 34 | 0.625 | 1 | 34 | 0.875 | 1 |
| 35 | 0.625 | 1 | 35 | 0.875 | 1 |
| 36 | 0.625 | 1 | 36 | 0.875 | 1 |
| 37 | 0.625 | 1 | 37 | 0.875 | 1 |
| 38 | 0.625 | 1 | 38 | 0.875 | 1 |
| 39 | 0.625 | 1 | 39 | 0.875 | 1 |
| 40 | 0.625 | 1 | 40 | 0.875 | 1 |
| 41 | 0.625 | 1 | 41 | 0.875 | 1 |
| 42 | 0.625 | 1 | 42 | 0.875 | 1 |
| 43 | 0.625 | 1 | 43 | 0.875 | 1 |
| 44 | 0.625 | 1 | 44 | 0.875 | 1 |
| 45 | 0.625 | 1 | 45 | 0.875 | 1 |
| 46 | 0.625 | 1 | 46 | 0.875 | 1 |
| 47 | 0.625 | 1 | 47 | 0.875 | 1 |
| 48 | 0.625 | 1 | 48 | 0.875 | 1 |
| 49 | 0.625 | 1 | 49 | 0.875 | 1 |
| 50 | 0.625 | 1 | 50 | 0.875 | 1 |
| 51 | 0.625 | 1 | 51 | 0.875 | 1 |
| 52 | 0.625 | 1 | 52 | 0.875 | 1 |
| 53 | 0.625 | 1 | 53 | 0.875 | 1 |
| 54 | 0.875 | 1 | 54 | 0.875 | 1 |
| 55 | 0.875 | 1 | 55 | 0.875 | 1 |
| 56 | 0.875 | 1 | 56 | 0.875 | 1 |
| 57 | 0.875 | 1 | 57 | 0.875 | 1 |
| 58 | 0.875 | 1 | 58 | 0.875 | 1 |
| 59 | 0.875 | 1 | 59 | 0.875 | 1 |
| 60 | 0.875 | 1 | 60 | 1.125 | 1 |
| 61 | 0.875 | 1 | 61 | 1.125 | 1 |
| 62 | 0.875 | 1 | 62 | 1.125 | 1 |
| 63 | 0.875 | 1 | 63 | 1.125 | 1 |
| 64 | 0.875 | 1 | 64 | 1.125 | 1 |
| 65 | 0.875 | 1 | 65 | 1.125 | 1 |
| 66 | 0.875 | 1 | 66 | 1.125 | 1 |
| 67 | 0.875 | 1 | 67 | 1.125 | 1 |
| 68 | 0.875 | 1 | 68 | 1.125 | 1 |
| 69 | 0.875 | 1 | 69 | 1.125 | 1 |
| 70 | 0.875 | 1 | 70 | 1.125 | 1 |
| 71 | 0.875 | 1 | 71 | 1.125 | 1 |
| 72 | 0.875 | 1 | 72 | 1.125 | 1 |
| 73 | 0.875 | 1 | 73 | 1.125 | 1 |
| 74 | 0.875 | 1 | 74 | 1.125 | 1 |
| 75 | 0.875 | 1 | 75 | 1.375 | 1 |
| 76 | 0.875 | 1 | 76 | 1.375 | 1 |
| 77 | 0.875 | 1 | 77 | 1.375 | 1 |
| 78 | 0.875 | 1 | 78 | 1.375 | 1 |
| 79 | 0.875 | 1 | 79 | 1.375 | 1 |
| 80 | 1.125 | 1 | 80 | 1.375 | 1 |
| 81 | 1.125 | 1 | 81 | 1.375 | 1 |
| 82 | 1.125 | 1 | 82 | 1.375 | 1 |
| 83 | 1.125 | 1 | 83 | 1.375 | 1 |
| 84 | 1.125 | 1 | 84 | 1.375 | 1 |
| 85 | 1.125 | 1 | 85 | 1.375 | 1 |
| 86 | 1.125 | 1 | 86 | 1.375 | 1 |
| 87 | 1.125 | 1 | 87 | 1.375 | 1 |
| 88 | 1.125 | 1 | 88 | 1.375 | 1 |

| | |
|---|---|
| ID | Patient ID |
| Follow_up | Follow up time in years |
| Event | 0 = no recurrence |
| | 1 = recurrence |

| | | |
|---|---|---|
| 89 | 1.125 | 1 |
| 90 | 1.125 | 1 |
| 91 | 1.125 | 1 |
| 92 | 1.125 | 1 |
| 93 | 1.125 | 1 |
| 94 | 1.125 | 1 |
| 95 | 1.125 | 1 |
| 96 | 1.125 | 1 |
| 97 | 1.125 | 1 |
| 98 | 1.125 | 1 |
| 99 | 1.125 | 1 |
| 100 | 1.125 | 1 |
| 101 | 1.125 | 1 |
| 102 | 1.125 | 1 |
| 103 | 1.125 | 1 |
| 104 | 1.125 | 1 |
| 105 | 1.125 | 1 |
| 106 | 1.125 | 1 |
| 107 | 1.125 | 1 |
| 108 | 1.125 | 1 |
| 109 | 1.125 | 1 |
| 110 | 1.375 | 1 |
| 111 | 1.375 | 1 |
| 112 | 1.375 | 1 |
| 113 | 1.375 | 1 |
| 114 | 1.375 | 1 |
| 115 | 1.375 | 1 |
| 116 | 1.375 | 1 |
| 117 | 1.375 | 1 |
| 118 | 1.375 | 1 |
| 119 | 1.375 | 1 |
| 120 | 1.375 | 1 |
| 121 | 1.375 | 1 |
| 122 | 1.375 | 1 |
| 123 | 1.375 | 1 |
| 124 | 1.375 | 1 |
| 125 | 1.375 | 1 |
| 126 | 1.375 | 1 |
| 127 | 1.375 | 1 |
| 128 | 1.375 | 1 |
| 129 | 1.375 | 1 |
| 130 | 1.375 | 1 |
| 131 | 1.375 | 1 |
| 132 | 1.375 | 1 |
| 133 | 1.375 | 1 |
| 134 | 1.375 | 1 |
| 135 | 1.375 | 1 |
| 136 | 1.375 | 1 |
| 137 | 1.375 | 1 |
| 138 | 1.375 | 1 |
| 139 | 1.625 | 1 |
| 140 | 1.625 | 1 |
| 141 | 1.625 | 1 |
| 142 | 1.625 | 1 |
| 143 | 1.625 | 1 |
| 144 | 1.625 | 1 |
| 145 | 1.625 | 1 |
| 146 | 1.625 | 1 |
| 147 | 1.625 | 1 |
| 148 | 1.625 | 1 |
| 149 | 1.625 | 1 |
| 150 | 1.625 | 1 |
| 151 | 1.625 | 1 |
| 152 | 1.625 | 1 |
| 153 | 1.625 | 1 |
| 154 | 1.625 | 1 |
| 155 | 1.625 | 1 |
| 156 | 1.625 | 1 |
| 157 | 1.625 | 1 |
| 158 | 1.625 | 1 |
| 159 | 1.625 | 1 |
| 160 | 1.625 | 1 |
| 161 | 1.625 | 1 |
| 162 | 1.875 | 1 |
| 163 | 1.875 | 1 |
| 164 | 1.875 | 1 |
| 165 | 1.875 | 1 |
| 166 | 1.875 | 1 |
| 167 | 1.875 | 1 |
| 168 | 1.875 | 1 |
| 169 | 1.875 | 1 |
| 170 | 1.875 | 1 |
| 171 | 1.875 | 1 |
| 172 | 1.875 | 1 |
| 173 | 1.875 | 1 |
| 174 | 1.875 | 1 |
| 175 | 1.875 | 1 |
| 176 | 1.875 | 1 |
| 177 | 1.875 | 1 |
| 178 | 1.875 | 1 |
| 179 | 1.875 | 1 |
| 180 | 1.875 | 1 |
| 181 | 1.875 | 1 |

| | | |
|---|---|---|
| 89 | 1.375 | 1 |
| 90 | 1.625 | 1 |
| 91 | 1.625 | 1 |
| 92 | 1.625 | 1 |
| 93 | 1.625 | 1 |
| 94 | 1.625 | 1 |
| 95 | 1.625 | 1 |
| 96 | 1.625 | 1 |
| 97 | 1.625 | 1 |
| 98 | 1.625 | 1 |
| 99 | 1.625 | 1 |
| 100 | 1.625 | 1 |
| 101 | 1.625 | 1 |
| 102 | 1.625 | 1 |
| 103 | 1.625 | 1 |
| 104 | 1.625 | 1 |
| 105 | 1.625 | 1 |
| 106 | 1.625 | 1 |
| 107 | 1.625 | 1 |
| 108 | 1.625 | 1 |
| 109 | 1.875 | 1 |
| 110 | 1.875 | 1 |
| 111 | 1.875 | 1 |
| 112 | 1.875 | 1 |
| 113 | 1.875 | 1 |
| 114 | 1.875 | 1 |
| 115 | 1.875 | 1 |
| 116 | 1.875 | 1 |
| 117 | 1.875 | 1 |
| 118 | 1.875 | 1 |
| 119 | 1.875 | 1 |
| 120 | 1.875 | 1 |
| 121 | 1.875 | 1 |
| 122 | 1.875 | 1 |
| 123 | 1.875 | 1 |
| 124 | 1.875 | 1 |
| 125 | 1.875 | 1 |
| 126 | 1.875 | 1 |
| 127 | 2.125 | 1 |
| 128 | 2.125 | 1 |
| 129 | 2.125 | 1 |
| 130 | 2.125 | 1 |
| 131 | 2.125 | 1 |
| 132 | 2.125 | 1 |
| 133 | 2.125 | 1 |
| 134 | 2.125 | 1 |
| 135 | 2.125 | 1 |
| 136 | 2.375 | 1 |
| 137 | 2.375 | 1 |
| 138 | 2.375 | 1 |
| 139 | 2.375 | 1 |
| 140 | 2.375 | 1 |
| 141 | 2.375 | 1 |
| 142 | 2.375 | 1 |
| 143 | 2.375 | 1 |
| 144 | 2.375 | 1 |
| 145 | 2.375 | 1 |
| 146 | 2.375 | 1 |
| 147 | 2.375 | 1 |
| 148 | 2.375 | 1 |
| 149 | 2.625 | 1 |
| 150 | 2.625 | 1 |
| 151 | 2.625 | 1 |
| 152 | 2.625 | 1 |
| 153 | 2.625 | 1 |
| 154 | 2.625 | 1 |
| 155 | 2.625 | 1 |
| 156 | 2.625 | 1 |
| 157 | 2.875 | 1 |
| 158 | 2.875 | 1 |
| 159 | 2.875 | 1 |
| 160 | 2.875 | 1 |
| 161 | 2.875 | 1 |
| 162 | 2.875 | 1 |
| 163 | 2.875 | 1 |
| 164 | 2.875 | 1 |
| 165 | 2.875 | 1 |
| 166 | 2.875 | 1 |
| 167 | 2.875 | 1 |
| 168 | 2.875 | 1 |
| 169 | 3.125 | 1 |
| 170 | 3.125 | 1 |
| 171 | 3.125 | 1 |
| 172 | 3.125 | 1 |
| 173 | 3.125 | 1 |
| 174 | 3.125 | 1 |
| 175 | 3.125 | 1 |
| 176 | 3.125 | 1 |
| 177 | 3.125 | 1 |
| 178 | 3.125 | 1 |
| 179 | 3.125 | 1 |
| 180 | 3.375 | 1 |
| 181 | 3.375 | 1 |

| | | |
|---|---|---|
| 182 | 1.875 | 1 |
| 183 | 1.875 | 1 |
| 184 | 1.875 | 1 |
| 185 | 2.125 | 1 |
| 186 | 2.125 | 1 |
| 187 | 2.125 | 1 |
| 188 | 2.125 | 1 |
| 189 | 2.125 | 1 |
| 190 | 2.125 | 1 |
| 191 | 2.125 | 1 |
| 192 | 2.125 | 1 |
| 193 | 2.125 | 1 |
| 194 | 2.125 | 1 |
| 195 | 2.125 | 1 |
| 196 | 2.125 | 1 |
| 197 | 2.125 | 1 |
| 198 | 2.375 | 1 |
| 199 | 2.375 | 1 |
| 200 | 2.375 | 1 |
| 201 | 2.375 | 1 |
| 202 | 2.375 | 1 |
| 203 | 2.375 | 1 |
| 204 | 2.375 | 1 |
| 205 | 2.375 | 1 |
| 206 | 2.375 | 1 |
| 207 | 2.375 | 1 |
| 208 | 2.375 | 1 |
| 209 | 2.375 | 1 |
| 210 | 2.375 | 1 |
| 211 | 2.625 | 1 |
| 212 | 2.625 | 1 |
| 213 | 2.625 | 1 |
| 214 | 2.625 | 1 |
| 215 | 2.625 | 1 |
| 216 | 2.625 | 1 |
| 217 | 2.625 | 1 |
| 218 | 2.625 | 1 |
| 219 | 2.625 | 1 |
| 220 | 2.625 | 1 |
| 221 | 2.625 | 1 |
| 222 | 2.625 | 1 |
| 223 | 2.625 | 1 |
| 224 | 2.625 | 1 |
| 225 | 2.625 | 1 |
| 226 | 2.625 | 1 |
| 227 | 2.875 | 1 |
| 228 | 2.875 | 1 |
| 229 | 2.875 | 1 |
| 230 | 2.875 | 1 |
| 231 | 2.875 | 1 |
| 232 | 2.875 | 1 |
| 233 | 2.875 | 1 |
| 234 | 2.875 | 1 |
| 235 | 2.875 | 1 |
| 236 | 2.875 | 1 |
| 237 | 2.875 | 1 |
| 238 | 2.875 | 1 |
| 239 | 3.125 | 1 |
| 240 | 3.125 | 1 |
| 241 | 3.125 | 1 |
| 242 | 3.125 | 1 |
| 243 | 3.125 | 1 |
| 244 | 3.125 | 1 |
| 245 | 3.125 | 1 |
| 246 | 3.125 | 1 |
| 247 | 3.375 | 1 |
| 248 | 3.375 | 1 |
| 249 | 3.375 | 1 |
| 250 | 3.375 | 1 |
| 251 | 3.375 | 1 |
| 252 | 3.375 | 1 |
| 253 | 3.375 | 1 |
| 254 | 3.625 | 1 |
| 255 | 3.625 | 1 |
| 256 | 3.625 | 1 |
| 257 | 3.625 | 1 |
| 258 | 3.625 | 1 |
| 259 | 3.625 | 1 |
| 260 | 3.625 | 1 |
| 261 | 3.875 | 1 |
| 262 | 3.875 | 1 |
| 263 | 3.875 | 1 |
| 264 | 3.875 | 1 |
| 265 | 3.875 | 1 |
| 266 | 3.875 | 1 |
| 267 | 3.875 | 1 |
| 268 | 4.125 | 1 |
| 269 | 4.125 | 1 |
| 270 | 4.125 | 1 |
| 271 | 4.375 | 1 |
| 272 | 4.375 | 1 |
| 273 | 4.375 | 1 |
| 274 | 4.625 | 1 |

| | | |
|---|---|---|
| 182 | 3.375 | 1 |
| 183 | 3.375 | 1 |
| 184 | 3.375 | 1 |
| 185 | 3.375 | 1 |
| 186 | 3.375 | 1 |
| 187 | 3.375 | 1 |
| 188 | 3.375 | 1 |
| 189 | 3.375 | 1 |
| 190 | 3.375 | 1 |
| 191 | 3.625 | 1 |
| 192 | 3.625 | 1 |
| 193 | 3.625 | 1 |
| 194 | 3.625 | 1 |
| 195 | 3.875 | 1 |
| 196 | 3.875 | 1 |
| 197 | 3.875 | 1 |
| 198 | 3.875 | 1 |
| 199 | 3.875 | 1 |
| 200 | 3.875 | 1 |
| 201 | 3.875 | 1 |
| 202 | 3.875 | 1 |
| 203 | 3.875 | 1 |
| 204 | 3.875 | 1 |
| 205 | 4.125 | 1 |
| 206 | 4.125 | 1 |
| 207 | 4.125 | 1 |
| 208 | 4.125 | 1 |
| 209 | 4.125 | 1 |
| 210 | 4.125 | 1 |
| 211 | 4.125 | 1 |
| 212 | 4.125 | 1 |
| 213 | 4.125 | 1 |
| 214 | 4.125 | 1 |
| 215 | 4.375 | 1 |
| 216 | 4.375 | 1 |
| 217 | 4.375 | 1 |
| 218 | 4.375 | 1 |
| 219 | 4.375 | 1 |
| 220 | 4.375 | 1 |
| 221 | 4.625 | 1 |
| 222 | 4.625 | 1 |
| 223 | 4.625 | 1 |
| 224 | 4.625 | 1 |
| 225 | 4.625 | 1 |
| 226 | 4.625 | 1 |
| 227 | 4.875 | 1 |
| 228 | 4.875 | 1 |
| 229 | 4.875 | 1 |
| 230 | 5.875 | 1 |
| 231 | 5.875 | 1 |
| 232 | 5.875 | 1 |
| 233 | 5.875 | 1 |
| 234 | 6.125 | 1 |
| 235 | 6.125 | 1 |
| 236 | 6.375 | 1 |
| 237 | 6.375 | 1 |
| 238 | 6.625 | 1 |
| 239 | 6.625 | 1 |
| 240 | 6.875 | 1 |
| 241 | 7.875 | 1 |
| 242 | 0.125 | 0 |
| 243 | 0.125 | 0 |
| 244 | 0.125 | 0 |
| 245 | 0.125 | 0 |
| 246 | 0.125 | 0 |
| 247 | 0.125 | 0 |
| 248 | 0.125 | 0 |
| 249 | 0.125 | 0 |
| 250 | 0.125 | 0 |
| 251 | 0.125 | 0 |
| 252 | 0.125 | 0 |
| 253 | 0.125 | 0 |
| 254 | 0.125 | 0 |
| 255 | 0.125 | 0 |
| 256 | 0.125 | 0 |
| 257 | 0.125 | 0 |
| 258 | 0.125 | 0 |
| 259 | 0.125 | 0 |
| 260 | 0.125 | 0 |
| 261 | 0.125 | 0 |
| 262 | 0.125 | 0 |
| 263 | 0.125 | 0 |
| 264 | 0.125 | 0 |
| 265 | 0.125 | 0 |
| 266 | 0.125 | 0 |
| 267 | 0.125 | 0 |
| 268 | 0.125 | 0 |
| 269 | 0.125 | 0 |
| 270 | 0.125 | 0 |
| 271 | 0.125 | 0 |
| 272 | 0.125 | 0 |
| 273 | 0.375 | 0 |
| 274 | 0.375 | 0 |

| | | |
|---|---|---|
| 275 | 4.625 | 1 |
| 276 | 4.625 | 1 |
| 277 | 4.625 | 1 |
| 278 | 4.625 | 1 |
| 279 | 4.625 | 1 |
| 280 | 5.125 | 1 |
| 281 | 5.125 | 1 |
| 282 | 5.125 | 1 |
| 283 | 5.375 | 1 |
| 284 | 5.375 | 1 |
| 285 | 5.375 | 1 |
| 286 | 5.625 | 1 |
| 287 | 5.625 | 1 |
| 288 | 5.875 | 1 |
| 289 | 5.875 | 1 |
| 290 | 6.125 | 1 |
| 291 | 6.125 | 1 |
| 292 | 6.375 | 1 |
| 293 | 6.375 | 1 |
| 294 | 6.625 | 1 |
| 295 | 6.625 | 1 |
| 296 | 7.125 | 1 |
| 297 | 7.375 | 1 |
| 298 | 7.625 | 1 |
| 299 | 8.625 | 1 |
| 300 | 8.875 | 1 |
| 301 | 0.125 | 0 |
| 302 | 0.125 | 0 |
| 303 | 0.125 | 0 |
| 304 | 0.125 | 0 |
| 305 | 0.125 | 0 |
| 306 | 0.125 | 0 |
| 307 | 0.125 | 0 |
| 308 | 0.125 | 0 |
| 309 | 0.125 | 0 |
| 310 | 0.125 | 0 |
| 311 | 0.125 | 0 |
| 312 | 0.125 | 0 |
| 313 | 0.125 | 0 |
| 314 | 0.125 | 0 |
| 315 | 0.125 | 0 |
| 316 | 0.125 | 0 |
| 317 | 0.125 | 0 |
| 318 | 0.125 | 0 |
| 319 | 0.125 | 0 |
| 320 | 0.125 | 0 |
| 321 | 0.125 | 0 |
| 322 | 0.125 | 0 |
| 323 | 0.125 | 0 |
| 324 | 0.125 | 0 |
| 325 | 0.125 | 0 |
| 326 | 0.125 | 0 |
| 327 | 0.125 | 0 |
| 328 | 0.125 | 0 |
| 329 | 0.125 | 0 |
| 330 | 0.125 | 0 |
| 331 | 0.375 | 0 |
| 332 | 0.375 | 0 |
| 333 | 0.375 | 0 |
| 334 | 0.375 | 0 |
| 335 | 0.375 | 0 |
| 336 | 0.375 | 0 |
| 337 | 0.375 | 0 |
| 338 | 0.375 | 0 |
| 339 | 0.375 | 0 |
| 340 | 0.375 | 0 |
| 341 | 0.375 | 0 |
| 342 | 0.375 | 0 |
| 343 | 0.375 | 0 |
| 344 | 0.375 | 0 |
| 345 | 0.375 | 0 |
| 346 | 0.375 | 0 |
| 347 | 0.375 | 0 |
| 348 | 0.375 | 0 |
| 349 | 0.375 | 0 |
| 350 | 0.375 | 0 |
| 351 | 0.375 | 0 |
| 352 | 0.375 | 0 |
| 353 | 0.375 | 0 |
| 354 | 0.375 | 0 |
| 355 | 0.375 | 0 |
| 356 | 0.375 | 0 |
| 357 | 0.375 | 0 |
| 358 | 0.375 | 0 |
| 359 | 0.375 | 0 |
| 360 | 0.375 | 0 |
| 361 | 0.625 | 0 |
| 362 | 0.625 | 0 |
| 363 | 0.625 | 0 |
| 364 | 0.625 | 0 |
| 365 | 0.625 | 0 |
| 366 | 0.625 | 0 |
| 367 | 0.625 | 0 |

| | | |
|---|---|---|
| 275 | 0.375 | 0 |
| 276 | 0.375 | 0 |
| 277 | 0.375 | 0 |
| 278 | 0.375 | 0 |
| 279 | 0.375 | 0 |
| 280 | 0.375 | 0 |
| 281 | 0.375 | 0 |
| 282 | 0.375 | 0 |
| 283 | 0.375 | 0 |
| 284 | 0.375 | 0 |
| 285 | 0.375 | 0 |
| 286 | 0.375 | 0 |
| 287 | 0.375 | 0 |
| 288 | 0.375 | 0 |
| 289 | 0.375 | 0 |
| 290 | 0.375 | 0 |
| 291 | 0.375 | 0 |
| 292 | 0.375 | 0 |
| 293 | 0.375 | 0 |
| 294 | 0.375 | 0 |
| 295 | 0.375 | 0 |
| 296 | 0.375 | 0 |
| 297 | 0.375 | 0 |
| 298 | 0.375 | 0 |
| 299 | 0.375 | 0 |
| 300 | 0.375 | 0 |
| 301 | 0.375 | 0 |
| 302 | 0.375 | 0 |
| 303 | 0.375 | 0 |
| 304 | 0.625 | 0 |
| 305 | 0.625 | 0 |
| 306 | 0.625 | 0 |
| 307 | 0.625 | 0 |
| 308 | 0.625 | 0 |
| 309 | 0.625 | 0 |
| 310 | 0.625 | 0 |
| 311 | 0.625 | 0 |
| 312 | 0.625 | 0 |
| 313 | 0.625 | 0 |
| 314 | 0.625 | 0 |
| 315 | 0.625 | 0 |
| 316 | 0.625 | 0 |
| 317 | 0.625 | 0 |
| 318 | 0.625 | 0 |
| 319 | 0.625 | 0 |
| 320 | 0.625 | 0 |
| 321 | 0.625 | 0 |
| 322 | 0.625 | 0 |
| 323 | 0.625 | 0 |
| 324 | 0.625 | 0 |
| 325 | 0.625 | 0 |
| 326 | 0.625 | 0 |
| 327 | 0.625 | 0 |
| 328 | 0.625 | 0 |
| 329 | 0.625 | 0 |
| 330 | 0.625 | 0 |
| 331 | 0.625 | 0 |
| 332 | 0.625 | 0 |
| 333 | 0.625 | 0 |
| 334 | 0.625 | 0 |
| 335 | 0.875 | 0 |
| 336 | 0.875 | 0 |
| 337 | 0.875 | 0 |
| 338 | 0.875 | 0 |
| 339 | 0.875 | 0 |
| 340 | 0.875 | 0 |
| 341 | 0.875 | 0 |
| 342 | 0.875 | 0 |
| 343 | 0.875 | 0 |
| 344 | 0.875 | 0 |
| 345 | 0.875 | 0 |
| 346 | 0.875 | 0 |
| 347 | 0.875 | 0 |
| 348 | 0.875 | 0 |
| 349 | 0.875 | 0 |
| 350 | 0.875 | 0 |
| 351 | 0.875 | 0 |
| 352 | 0.875 | 0 |
| 353 | 0.875 | 0 |
| 354 | 0.875 | 0 |
| 355 | 0.875 | 0 |
| 356 | 0.875 | 0 |
| 357 | 0.875 | 0 |
| 358 | 0.875 | 0 |
| 359 | 0.875 | 0 |
| 360 | 0.875 | 0 |
| 361 | 0.875 | 0 |
| 362 | 0.875 | 0 |
| 363 | 0.875 | 0 |
| 364 | 0.875 | 0 |
| 365 | 0.875 | 0 |
| 366 | 1.125 | 0 |
| 367 | 1.125 | 0 |

| | | |
|---|---|---|
| 368 | 0.625 | 0 |
| 369 | 0.625 | 0 |
| 370 | 0.625 | 0 |
| 371 | 0.625 | 0 |
| 372 | 0.625 | 0 |
| 373 | 0.625 | 0 |
| 374 | 0.625 | 0 |
| 375 | 0.625 | 0 |
| 376 | 0.625 | 0 |
| 377 | 0.625 | 0 |
| 378 | 0.625 | 0 |
| 379 | 0.625 | 0 |
| 380 | 0.625 | 0 |
| 381 | 0.625 | 0 |
| 382 | 0.625 | 0 |
| 383 | 0.625 | 0 |
| 384 | 0.625 | 0 |
| 385 | 0.625 | 0 |
| 386 | 0.625 | 0 |
| 387 | 0.625 | 0 |
| 388 | 0.625 | 0 |
| 389 | 0.625 | 0 |
| 390 | 0.625 | 0 |
| 391 | 0.875 | 0 |
| 392 | 0.875 | 0 |
| 393 | 0.875 | 0 |
| 394 | 0.875 | 0 |
| 395 | 0.875 | 0 |
| 396 | 0.875 | 0 |
| 397 | 0.875 | 0 |
| 398 | 0.875 | 0 |
| 399 | 0.875 | 0 |
| 400 | 0.875 | 0 |
| 401 | 0.875 | 0 |
| 402 | 0.875 | 0 |
| 403 | 0.875 | 0 |
| 404 | 0.875 | 0 |
| 405 | 0.875 | 0 |
| 406 | 0.875 | 0 |
| 407 | 0.875 | 0 |
| 408 | 0.875 | 0 |
| 409 | 0.875 | 0 |
| 410 | 0.875 | 0 |
| 411 | 0.875 | 0 |
| 412 | 0.875 | 0 |
| 413 | 0.875 | 0 |
| 414 | 0.875 | 0 |
| 415 | 0.875 | 0 |
| 416 | 0.875 | 0 |
| 417 | 0.875 | 0 |
| 418 | 0.875 | 0 |
| 419 | 0.875 | 0 |
| 420 | 0.875 | 0 |
| 421 | 1.125 | 0 |
| 422 | 1.125 | 0 |
| 423 | 1.125 | 0 |
| 424 | 1.125 | 0 |
| 425 | 1.125 | 0 |
| 426 | 1.125 | 0 |
| 427 | 1.125 | 0 |
| 428 | 1.125 | 0 |
| 429 | 1.125 | 0 |
| 430 | 1.125 | 0 |
| 431 | 1.125 | 0 |
| 432 | 1.125 | 0 |
| 433 | 1.125 | 0 |
| 434 | 1.125 | 0 |
| 435 | 1.125 | 0 |
| 436 | 1.125 | 0 |
| 437 | 1.125 | 0 |
| 438 | 1.125 | 0 |
| 439 | 1.125 | 0 |
| 440 | 1.125 | 0 |
| 441 | 1.125 | 0 |
| 442 | 1.125 | 0 |
| 443 | 1.125 | 0 |
| 444 | 1.125 | 0 |
| 445 | 1.125 | 0 |
| 446 | 1.125 | 0 |
| 447 | 1.125 | 0 |
| 448 | 1.125 | 0 |
| 449 | 1.125 | 0 |
| 450 | 1.125 | 0 |
| 451 | 1.125 | 0 |
| 452 | 1.125 | 0 |
| 453 | 1.125 | 0 |
| 454 | 1.125 | 0 |
| 455 | 1.125 | 0 |
| 456 | 1.125 | 0 |
| 457 | 1.125 | 0 |
| 458 | 1.125 | 0 |
| 459 | 1.125 | 0 |
| 460 | 1.125 | 0 |

| | | |
|---|---|---|
| 368 | 1.125 | 0 |
| 369 | 1.125 | 0 |
| 370 | 1.125 | 0 |
| 371 | 1.125 | 0 |
| 372 | 1.125 | 0 |
| 373 | 1.125 | 0 |
| 374 | 1.125 | 0 |
| 375 | 1.125 | 0 |
| 376 | 1.125 | 0 |
| 377 | 1.125 | 0 |
| 378 | 1.125 | 0 |
| 379 | 1.125 | 0 |
| 380 | 1.125 | 0 |
| 381 | 1.125 | 0 |
| 382 | 1.125 | 0 |
| 383 | 1.125 | 0 |
| 384 | 1.125 | 0 |
| 385 | 1.125 | 0 |
| 386 | 1.125 | 0 |
| 387 | 1.125 | 0 |
| 388 | 1.125 | 0 |
| 389 | 1.125 | 0 |
| 390 | 1.125 | 0 |
| 391 | 1.125 | 0 |
| 392 | 1.125 | 0 |
| 393 | 1.125 | 0 |
| 394 | 1.125 | 0 |
| 395 | 1.125 | 0 |
| 396 | 1.125 | 0 |
| 397 | 1.125 | 0 |
| 398 | 1.125 | 0 |
| 399 | 1.125 | 0 |
| 400 | 1.125 | 0 |
| 401 | 1.125 | 0 |
| 402 | 1.125 | 0 |
| 403 | 1.125 | 0 |
| 404 | 1.125 | 0 |
| 405 | 1.375 | 0 |
| 406 | 1.375 | 0 |
| 407 | 1.375 | 0 |
| 408 | 1.375 | 0 |
| 409 | 1.375 | 0 |
| 410 | 1.375 | 0 |
| 411 | 1.375 | 0 |
| 412 | 1.375 | 0 |
| 413 | 1.375 | 0 |
| 414 | 1.375 | 0 |
| 415 | 1.375 | 0 |
| 416 | 1.375 | 0 |
| 417 | 1.375 | 0 |
| 418 | 1.375 | 0 |
| 419 | 1.375 | 0 |
| 420 | 1.375 | 0 |
| 421 | 1.375 | 0 |
| 422 | 1.375 | 0 |
| 423 | 1.375 | 0 |
| 424 | 1.375 | 0 |
| 425 | 1.375 | 0 |
| 426 | 1.375 | 0 |
| 427 | 1.375 | 0 |
| 428 | 1.375 | 0 |
| 429 | 1.375 | 0 |
| 430 | 1.375 | 0 |
| 431 | 1.375 | 0 |
| 432 | 1.375 | 0 |
| 433 | 1.375 | 0 |
| 434 | 1.375 | 0 |
| 435 | 1.375 | 0 |
| 436 | 1.375 | 0 |
| 437 | 1.375 | 0 |
| 438 | 1.375 | 0 |
| 439 | 1.375 | 0 |
| 440 | 1.375 | 0 |
| 441 | 1.375 | 0 |
| 442 | 1.375 | 0 |
| 443 | 1.375 | 0 |
| 444 | 1.625 | 0 |
| 445 | 1.625 | 0 |
| 446 | 1.625 | 0 |
| 447 | 1.625 | 0 |
| 448 | 1.625 | 0 |
| 449 | 1.625 | 0 |
| 450 | 1.625 | 0 |
| 451 | 1.625 | 0 |
| 452 | 1.625 | 0 |
| 453 | 1.625 | 0 |
| 454 | 1.625 | 0 |
| 455 | 1.625 | 0 |
| 456 | 1.625 | 0 |
| 457 | 1.625 | 0 |
| 458 | 1.625 | 0 |
| 459 | 1.625 | 0 |
| 460 | 1.625 | 0 |

| | | |
|---|---|---|
| 461 | 1.375 | 0 |
| 462 | 1.375 | 0 |
| 463 | 1.375 | 0 |
| 464 | 1.375 | 0 |
| 465 | 1.375 | 0 |
| 466 | 1.375 | 0 |
| 467 | 1.375 | 0 |
| 468 | 1.375 | 0 |
| 469 | 1.375 | 0 |
| 470 | 1.375 | 0 |
| 471 | 1.375 | 0 |
| 472 | 1.375 | 0 |
| 473 | 1.375 | 0 |
| 474 | 1.375 | 0 |
| 475 | 1.375 | 0 |
| 476 | 1.375 | 0 |
| 477 | 1.375 | 0 |
| 478 | 1.375 | 0 |
| 479 | 1.375 | 0 |
| 480 | 1.375 | 0 |
| 481 | 1.375 | 0 |
| 482 | 1.375 | 0 |
| 483 | 1.375 | 0 |
| 484 | 1.375 | 0 |
| 485 | 1.375 | 0 |
| 486 | 1.375 | 0 |
| 487 | 1.375 | 0 |
| 488 | 1.375 | 0 |
| 489 | 1.375 | 0 |
| 490 | 1.375 | 0 |
| 491 | 1.375 | 0 |
| 492 | 1.375 | 0 |
| 493 | 1.375 | 0 |
| 494 | 1.375 | 0 |
| 495 | 1.375 | 0 |
| 496 | 1.375 | 0 |
| 497 | 1.375 | 0 |
| 498 | 1.375 | 0 |
| 499 | 1.375 | 0 |
| 500 | 1.375 | 0 |
| 501 | 1.625 | 0 |
| 502 | 1.625 | 0 |
| 503 | 1.625 | 0 |
| 504 | 1.625 | 0 |
| 505 | 1.625 | 0 |
| 506 | 1.625 | 0 |
| 507 | 1.625 | 0 |
| 508 | 1.625 | 0 |
| 509 | 1.625 | 0 |
| 510 | 1.625 | 0 |
| 511 | 1.625 | 0 |
| 512 | 1.625 | 0 |
| 513 | 1.625 | 0 |
| 514 | 1.625 | 0 |
| 515 | 1.625 | 0 |
| 516 | 1.625 | 0 |
| 517 | 1.625 | 0 |
| 518 | 1.625 | 0 |
| 519 | 1.625 | 0 |
| 520 | 1.625 | 0 |
| 521 | 1.625 | 0 |
| 522 | 1.625 | 0 |
| 523 | 1.625 | 0 |
| 524 | 1.625 | 0 |
| 525 | 1.625 | 0 |
| 526 | 1.625 | 0 |
| 527 | 1.625 | 0 |
| 528 | 1.625 | 0 |
| 529 | 1.625 | 0 |
| 530 | 1.625 | 0 |
| 531 | 1.625 | 0 |
| 532 | 1.625 | 0 |
| 533 | 1.625 | 0 |
| 534 | 1.625 | 0 |
| 535 | 1.625 | 0 |
| 536 | 1.625 | 0 |
| 537 | 1.625 | 0 |
| 538 | 1.625 | 0 |
| 539 | 1.625 | 0 |
| 540 | 1.625 | 0 |
| 541 | 1.875 | 0 |
| 542 | 1.875 | 0 |
| 543 | 1.875 | 0 |
| 544 | 1.875 | 0 |
| 545 | 1.875 | 0 |
| 546 | 1.875 | 0 |
| 547 | 1.875 | 0 |
| 548 | 1.875 | 0 |
| 549 | 1.875 | 0 |
| 550 | 1.875 | 0 |
| 551 | 1.875 | 0 |
| 552 | 1.875 | 0 |
| 553 | 1.875 | 0 |

| | | |
|---|---|---|
| 461 | 1.625 | 0 |
| 462 | 1.625 | 0 |
| 463 | 1.625 | 0 |
| 464 | 1.625 | 0 |
| 465 | 1.625 | 0 |
| 466 | 1.625 | 0 |
| 467 | 1.625 | 0 |
| 468 | 1.625 | 0 |
| 469 | 1.625 | 0 |
| 470 | 1.625 | 0 |
| 471 | 1.625 | 0 |
| 472 | 1.625 | 0 |
| 473 | 1.625 | 0 |
| 474 | 1.625 | 0 |
| 475 | 1.625 | 0 |
| 476 | 1.625 | 0 |
| 477 | 1.625 | 0 |
| 478 | 1.625 | 0 |
| 479 | 1.625 | 0 |
| 480 | 1.625 | 0 |
| 481 | 1.625 | 0 |
| 482 | 1.625 | 0 |
| 483 | 1.875 | 0 |
| 484 | 1.875 | 0 |
| 485 | 1.875 | 0 |
| 486 | 1.875 | 0 |
| 487 | 1.875 | 0 |
| 488 | 1.875 | 0 |
| 489 | 1.875 | 0 |
| 490 | 1.875 | 0 |
| 491 | 1.875 | 0 |
| 492 | 1.875 | 0 |
| 493 | 1.875 | 0 |
| 494 | 1.875 | 0 |
| 495 | 1.875 | 0 |
| 496 | 1.875 | 0 |
| 497 | 1.875 | 0 |
| 498 | 1.875 | 0 |
| 499 | 1.875 | 0 |
| 500 | 1.875 | 0 |
| 501 | 1.875 | 0 |
| 502 | 1.875 | 0 |
| 503 | 1.875 | 0 |
| 504 | 1.875 | 0 |
| 505 | 1.875 | 0 |
| 506 | 1.875 | 0 |
| 507 | 1.875 | 0 |
| 508 | 1.875 | 0 |
| 509 | 1.875 | 0 |
| 510 | 1.875 | 0 |
| 511 | 1.875 | 0 |
| 512 | 1.875 | 0 |
| 513 | 1.875 | 0 |
| 514 | 1.875 | 0 |
| 515 | 1.875 | 0 |
| 516 | 1.875 | 0 |
| 517 | 1.875 | 0 |
| 518 | 1.875 | 0 |
| 519 | 1.875 | 0 |
| 520 | 1.875 | 0 |
| 521 | 1.875 | 0 |
| 522 | 2.125 | 0 |
| 523 | 2.125 | 0 |
| 524 | 2.125 | 0 |
| 525 | 2.125 | 0 |
| 526 | 2.125 | 0 |
| 527 | 2.125 | 0 |
| 528 | 2.125 | 0 |
| 529 | 2.125 | 0 |
| 530 | 2.125 | 0 |
| 531 | 2.125 | 0 |
| 532 | 2.125 | 0 |
| 533 | 2.125 | 0 |
| 534 | 2.125 | 0 |
| 535 | 2.125 | 0 |
| 536 | 2.125 | 0 |
| 537 | 2.125 | 0 |
| 538 | 2.125 | 0 |
| 539 | 2.125 | 0 |
| 540 | 2.125 | 0 |
| 541 | 2.125 | 0 |
| 542 | 2.125 | 0 |
| 543 | 2.125 | 0 |
| 544 | 2.125 | 0 |
| 545 | 2.125 | 0 |
| 546 | 2.125 | 0 |
| 547 | 2.125 | 0 |
| 548 | 2.125 | 0 |
| 549 | 2.125 | 0 |
| 550 | 2.125 | 0 |
| 551 | 2.125 | 0 |
| 552 | 2.125 | 0 |
| 553 | 2.125 | 0 |

| | | |
|---|---|---|
| 554 | 1.875 | 0 |
| 555 | 1.875 | 0 |
| 556 | 1.875 | 0 |
| 557 | 1.875 | 0 |
| 558 | 1.875 | 0 |
| 559 | 1.875 | 0 |
| 560 | 1.875 | 0 |
| 561 | 1.875 | 0 |
| 562 | 1.875 | 0 |
| 563 | 1.875 | 0 |
| 564 | 1.875 | 0 |
| 565 | 1.875 | 0 |
| 566 | 1.875 | 0 |
| 567 | 1.875 | 0 |
| 568 | 1.875 | 0 |
| 569 | 1.875 | 0 |
| 570 | 1.875 | 0 |
| 571 | 1.875 | 0 |
| 572 | 1.875 | 0 |
| 573 | 1.875 | 0 |
| 574 | 1.875 | 0 |
| 575 | 1.875 | 0 |
| 576 | 1.875 | 0 |
| 577 | 1.875 | 0 |
| 578 | 1.875 | 0 |
| 579 | 1.875 | 0 |
| 580 | 1.875 | 0 |
| 581 | 2.125 | 0 |
| 582 | 2.125 | 0 |
| 583 | 2.125 | 0 |
| 584 | 2.125 | 0 |
| 585 | 2.125 | 0 |
| 586 | 2.125 | 0 |
| 587 | 2.125 | 0 |
| 588 | 2.125 | 0 |
| 589 | 2.125 | 0 |
| 590 | 2.125 | 0 |
| 591 | 2.125 | 0 |
| 592 | 2.125 | 0 |
| 593 | 2.125 | 0 |
| 594 | 2.125 | 0 |
| 595 | 2.125 | 0 |
| 596 | 2.125 | 0 |
| 597 | 2.125 | 0 |
| 598 | 2.125 | 0 |
| 599 | 2.125 | 0 |
| 600 | 2.125 | 0 |
| 601 | 2.125 | 0 |
| 602 | 2.125 | 0 |
| 603 | 2.125 | 0 |
| 604 | 2.125 | 0 |
| 605 | 2.125 | 0 |
| 606 | 2.125 | 0 |
| 607 | 2.125 | 0 |
| 608 | 2.125 | 0 |
| 609 | 2.125 | 0 |
| 610 | 2.125 | 0 |
| 611 | 2.125 | 0 |
| 612 | 2.375 | 0 |
| 613 | 2.375 | 0 |
| 614 | 2.375 | 0 |
| 615 | 2.375 | 0 |
| 616 | 2.375 | 0 |
| 617 | 2.375 | 0 |
| 618 | 2.375 | 0 |
| 619 | 2.375 | 0 |
| 620 | 2.375 | 0 |
| 621 | 2.375 | 0 |
| 622 | 2.375 | 0 |
| 623 | 2.375 | 0 |
| 624 | 2.375 | 0 |
| 625 | 2.375 | 0 |
| 626 | 2.375 | 0 |
| 627 | 2.375 | 0 |
| 628 | 2.375 | 0 |
| 629 | 2.375 | 0 |
| 630 | 2.375 | 0 |
| 631 | 2.375 | 0 |
| 632 | 2.375 | 0 |
| 633 | 2.375 | 0 |
| 634 | 2.375 | 0 |
| 635 | 2.375 | 0 |
| 636 | 2.375 | 0 |
| 637 | 2.375 | 0 |
| 638 | 2.375 | 0 |
| 639 | 2.375 | 0 |
| 640 | 2.375 | 0 |
| 641 | 2.375 | 0 |
| 642 | 2.375 | 0 |
| 643 | 2.625 | 0 |
| 644 | 2.625 | 0 |
| 645 | 2.625 | 0 |
| 646 | 2.625 | 0 |

| | | |
|---|---|---|
| 554 | 2.125 | 0 |
| 555 | 2.125 | 0 |
| 556 | 2.125 | 0 |
| 557 | 2.125 | 0 |
| 558 | 2.125 | 0 |
| 559 | 2.375 | 0 |
| 560 | 2.375 | 0 |
| 561 | 2.375 | 0 |
| 562 | 2.375 | 0 |
| 563 | 2.375 | 0 |
| 564 | 2.375 | 0 |
| 565 | 2.375 | 0 |
| 566 | 2.375 | 0 |
| 567 | 2.375 | 0 |
| 568 | 2.375 | 0 |
| 569 | 2.375 | 0 |
| 570 | 2.375 | 0 |
| 571 | 2.375 | 0 |
| 572 | 2.375 | 0 |
| 573 | 2.375 | 0 |
| 574 | 2.375 | 0 |
| 575 | 2.375 | 0 |
| 576 | 2.375 | 0 |
| 577 | 2.375 | 0 |
| 578 | 2.375 | 0 |
| 579 | 2.375 | 0 |
| 580 | 2.375 | 0 |
| 581 | 2.375 | 0 |
| 582 | 2.375 | 0 |
| 583 | 2.375 | 0 |
| 584 | 2.375 | 0 |
| 585 | 2.375 | 0 |
| 586 | 2.375 | 0 |
| 587 | 2.375 | 0 |
| 588 | 2.375 | 0 |
| 589 | 2.375 | 0 |
| 590 | 2.375 | 0 |
| 591 | 2.375 | 0 |
| 592 | 2.375 | 0 |
| 593 | 2.375 | 0 |
| 594 | 2.375 | 0 |
| 595 | 2.375 | 0 |
| 596 | 2.625 | 0 |
| 597 | 2.625 | 0 |
| 598 | 2.625 | 0 |
| 599 | 2.625 | 0 |
| 600 | 2.625 | 0 |
| 601 | 2.625 | 0 |
| 602 | 2.625 | 0 |
| 603 | 2.625 | 0 |
| 604 | 2.625 | 0 |
| 605 | 2.625 | 0 |
| 606 | 2.625 | 0 |
| 607 | 2.625 | 0 |
| 608 | 2.625 | 0 |
| 609 | 2.625 | 0 |
| 610 | 2.625 | 0 |
| 611 | 2.625 | 0 |
| 612 | 2.625 | 0 |
| 613 | 2.625 | 0 |
| 614 | 2.625 | 0 |
| 615 | 2.625 | 0 |
| 616 | 2.625 | 0 |
| 617 | 2.625 | 0 |
| 618 | 2.625 | 0 |
| 619 | 2.625 | 0 |
| 620 | 2.625 | 0 |
| 621 | 2.625 | 0 |
| 622 | 2.625 | 0 |
| 623 | 2.625 | 0 |
| 624 | 2.625 | 0 |
| 625 | 2.625 | 0 |
| 626 | 2.625 | 0 |
| 627 | 2.625 | 0 |
| 628 | 2.625 | 0 |
| 629 | 2.625 | 0 |
| 630 | 2.625 | 0 |
| 631 | 2.625 | 0 |
| 632 | 2.625 | 0 |
| 633 | 2.875 | 0 |
| 634 | 2.875 | 0 |
| 635 | 2.875 | 0 |
| 636 | 2.875 | 0 |
| 637 | 2.875 | 0 |
| 638 | 2.875 | 0 |
| 639 | 2.875 | 0 |
| 640 | 2.875 | 0 |
| 641 | 2.875 | 0 |
| 642 | 2.875 | 0 |
| 643 | 2.875 | 0 |
| 644 | 2.875 | 0 |
| 645 | 2.875 | 0 |
| 646 | 2.875 | 0 |

| | | |
|---|---|---|
| 647 | 2.625 | 0 |
| 648 | 2.625 | 0 |
| 649 | 2.625 | 0 |
| 650 | 2.625 | 0 |
| 651 | 2.625 | 0 |
| 652 | 2.625 | 0 |
| 653 | 2.625 | 0 |
| 654 | 2.625 | 0 |
| 655 | 2.625 | 0 |
| 656 | 2.625 | 0 |
| 657 | 2.625 | 0 |
| 658 | 2.625 | 0 |
| 659 | 2.625 | 0 |
| 660 | 2.625 | 0 |
| 661 | 2.625 | 0 |
| 662 | 2.625 | 0 |
| 663 | 2.625 | 0 |
| 664 | 2.625 | 0 |
| 665 | 2.625 | 0 |
| 666 | 2.625 | 0 |
| 667 | 2.625 | 0 |
| 668 | 2.625 | 0 |
| 669 | 2.625 | 0 |
| 670 | 2.625 | 0 |
| 671 | 2.625 | 0 |
| 672 | 2.625 | 0 |
| 673 | 2.625 | 0 |
| 674 | 2.875 | 0 |
| 675 | 2.875 | 0 |
| 676 | 2.875 | 0 |
| 677 | 2.875 | 0 |
| 678 | 2.875 | 0 |
| 679 | 2.875 | 0 |
| 680 | 2.875 | 0 |
| 681 | 2.875 | 0 |
| 682 | 2.875 | 0 |
| 683 | 2.875 | 0 |
| 684 | 2.875 | 0 |
| 685 | 2.875 | 0 |
| 686 | 2.875 | 0 |
| 687 | 2.875 | 0 |
| 688 | 2.875 | 0 |
| 689 | 2.875 | 0 |
| 690 | 2.875 | 0 |
| 691 | 2.875 | 0 |
| 692 | 2.875 | 0 |
| 693 | 2.875 | 0 |
| 694 | 2.875 | 0 |
| 695 | 2.875 | 0 |
| 696 | 2.875 | 0 |
| 697 | 2.875 | 0 |
| 698 | 2.875 | 0 |
| 699 | 2.875 | 0 |
| 700 | 2.875 | 0 |
| 701 | 2.875 | 0 |
| 702 | 2.875 | 0 |
| 703 | 2.875 | 0 |
| 704 | 2.875 | 0 |
| 705 | 3.125 | 0 |
| 706 | 3.125 | 0 |
| 707 | 3.125 | 0 |
| 708 | 3.125 | 0 |
| 709 | 3.125 | 0 |
| 710 | 3.125 | 0 |
| 711 | 3.125 | 0 |
| 712 | 3.125 | 0 |
| 713 | 3.125 | 0 |
| 714 | 3.125 | 0 |
| 715 | 3.125 | 0 |
| 716 | 3.125 | 0 |
| 717 | 3.125 | 0 |
| 718 | 3.125 | 0 |
| 719 | 3.125 | 0 |
| 720 | 3.125 | 0 |
| 721 | 3.125 | 0 |
| 722 | 3.125 | 0 |
| 723 | 3.125 | 0 |
| 724 | 3.125 | 0 |
| 725 | 3.125 | 0 |
| 726 | 3.125 | 0 |
| 727 | 3.125 | 0 |
| 728 | 3.125 | 0 |
| 729 | 3.125 | 0 |
| 730 | 3.125 | 0 |
| 731 | 3.125 | 0 |
| 732 | 3.125 | 0 |
| 733 | 3.125 | 0 |
| 734 | 3.125 | 0 |
| 735 | 3.375 | 0 |
| 736 | 3.375 | 0 |
| 737 | 3.375 | 0 |
| 738 | 3.375 | 0 |
| 739 | 3.375 | 0 |

| | | |
|---|---|---|
| 647 | 2.875 | 0 |
| 648 | 2.875 | 0 |
| 649 | 2.875 | 0 |
| 650 | 2.875 | 0 |
| 651 | 2.875 | 0 |
| 652 | 2.875 | 0 |
| 653 | 2.875 | 0 |
| 654 | 2.875 | 0 |
| 655 | 2.875 | 0 |
| 656 | 2.875 | 0 |
| 657 | 2.875 | 0 |
| 658 | 2.875 | 0 |
| 659 | 2.875 | 0 |
| 660 | 2.875 | 0 |
| 661 | 2.875 | 0 |
| 662 | 2.875 | 0 |
| 663 | 2.875 | 0 |
| 664 | 2.875 | 0 |
| 665 | 2.875 | 0 |
| 666 | 2.875 | 0 |
| 667 | 2.875 | 0 |
| 668 | 2.875 | 0 |
| 669 | 2.875 | 0 |
| 670 | 3.125 | 0 |
| 671 | 3.125 | 0 |
| 672 | 3.125 | 0 |
| 673 | 3.125 | 0 |
| 674 | 3.125 | 0 |
| 675 | 3.125 | 0 |
| 676 | 3.125 | 0 |
| 677 | 3.125 | 0 |
| 678 | 3.125 | 0 |
| 679 | 3.125 | 0 |
| 680 | 3.125 | 0 |
| 681 | 3.125 | 0 |
| 682 | 3.125 | 0 |
| 683 | 3.125 | 0 |
| 684 | 3.125 | 0 |
| 685 | 3.125 | 0 |
| 686 | 3.125 | 0 |
| 687 | 3.125 | 0 |
| 688 | 3.125 | 0 |
| 689 | 3.125 | 0 |
| 690 | 3.125 | 0 |
| 691 | 3.125 | 0 |
| 692 | 3.125 | 0 |
| 693 | 3.125 | 0 |
| 694 | 3.125 | 0 |
| 695 | 3.125 | 0 |
| 696 | 3.125 | 0 |
| 697 | 3.125 | 0 |
| 698 | 3.125 | 0 |
| 699 | 3.125 | 0 |
| 700 | 3.125 | 0 |
| 701 | 3.125 | 0 |
| 702 | 3.375 | 0 |
| 703 | 3.375 | 0 |
| 704 | 3.375 | 0 |
| 705 | 3.375 | 0 |
| 706 | 3.375 | 0 |
| 707 | 3.375 | 0 |
| 708 | 3.375 | 0 |
| 709 | 3.375 | 0 |
| 710 | 3.375 | 0 |
| 711 | 3.375 | 0 |
| 712 | 3.375 | 0 |
| 713 | 3.375 | 0 |
| 714 | 3.375 | 0 |
| 715 | 3.375 | 0 |
| 716 | 3.375 | 0 |
| 717 | 3.375 | 0 |
| 718 | 3.375 | 0 |
| 719 | 3.375 | 0 |
| 720 | 3.375 | 0 |
| 721 | 3.375 | 0 |
| 722 | 3.375 | 0 |
| 723 | 3.375 | 0 |
| 724 | 3.375 | 0 |
| 725 | 3.375 | 0 |
| 726 | 3.375 | 0 |
| 727 | 3.375 | 0 |
| 728 | 3.375 | 0 |
| 729 | 3.375 | 0 |
| 730 | 3.375 | 0 |
| 731 | 3.375 | 0 |
| 732 | 3.375 | 0 |
| 733 | 3.375 | 0 |
| 734 | 3.625 | 0 |
| 735 | 3.625 | 0 |
| 736 | 3.625 | 0 |
| 737 | 3.625 | 0 |
| 738 | 3.625 | 0 |
| 739 | 3.625 | 0 |

| | | |
|---|---|---|
| 740 | 3.375 | 0 |
| 741 | 3.375 | 0 |
| 742 | 3.375 | 0 |
| 743 | 3.375 | 0 |
| 744 | 3.375 | 0 |
| 745 | 3.375 | 0 |
| 746 | 3.375 | 0 |
| 747 | 3.375 | 0 |
| 748 | 3.375 | 0 |
| 749 | 3.375 | 0 |
| 750 | 3.375 | 0 |
| 751 | 3.375 | 0 |
| 752 | 3.375 | 0 |
| 753 | 3.375 | 0 |
| 754 | 3.375 | 0 |
| 755 | 3.375 | 0 |
| 756 | 3.375 | 0 |
| 757 | 3.375 | 0 |
| 758 | 3.375 | 0 |
| 759 | 3.375 | 0 |
| 760 | 3.375 | 0 |
| 761 | 3.375 | 0 |
| 762 | 3.375 | 0 |
| 763 | 3.375 | 0 |
| 764 | 3.375 | 0 |
| 765 | 3.625 | 0 |
| 766 | 3.625 | 0 |
| 767 | 3.625 | 0 |
| 768 | 3.625 | 0 |
| 769 | 3.625 | 0 |
| 770 | 3.625 | 0 |
| 771 | 3.625 | 0 |
| 772 | 3.625 | 0 |
| 773 | 3.625 | 0 |
| 774 | 3.625 | 0 |
| 775 | 3.625 | 0 |
| 776 | 3.625 | 0 |
| 777 | 3.625 | 0 |
| 778 | 3.625 | 0 |
| 779 | 3.625 | 0 |
| 780 | 3.625 | 0 |
| 781 | 3.625 | 0 |
| 782 | 3.625 | 0 |
| 783 | 3.625 | 0 |
| 784 | 3.625 | 0 |
| 785 | 3.625 | 0 |
| 786 | 3.625 | 0 |
| 787 | 3.625 | 0 |
| 788 | 3.625 | 0 |
| 789 | 3.625 | 0 |
| 790 | 3.625 | 0 |
| 791 | 3.625 | 0 |
| 792 | 3.625 | 0 |
| 793 | 3.625 | 0 |
| 794 | 3.625 | 0 |
| 795 | 3.875 | 0 |
| 796 | 3.875 | 0 |
| 797 | 3.875 | 0 |
| 798 | 3.875 | 0 |
| 799 | 3.875 | 0 |
| 800 | 3.875 | 0 |
| 801 | 3.875 | 0 |
| 802 | 3.875 | 0 |
| 803 | 3.875 | 0 |
| 804 | 3.875 | 0 |
| 805 | 3.875 | 0 |
| 806 | 3.875 | 0 |
| 807 | 3.875 | 0 |
| 808 | 3.875 | 0 |
| 809 | 3.875 | 0 |
| 810 | 3.875 | 0 |
| 811 | 3.875 | 0 |
| 812 | 3.875 | 0 |
| 813 | 3.875 | 0 |
| 814 | 3.875 | 0 |
| 815 | 3.875 | 0 |
| 816 | 3.875 | 0 |
| 817 | 3.875 | 0 |
| 818 | 3.875 | 0 |
| 819 | 3.875 | 0 |
| 820 | 3.875 | 0 |
| 821 | 3.875 | 0 |
| 822 | 3.875 | 0 |
| 823 | 3.875 | 0 |
| 824 | 3.875 | 0 |
| 825 | 4.125 | 0 |
| 826 | 4.125 | 0 |
| 827 | 4.125 | 0 |
| 828 | 4.125 | 0 |
| 829 | 4.125 | 0 |
| 830 | 4.125 | 0 |
| 831 | 4.125 | 0 |
| 832 | 4.125 | 0 |

| | | |
|---|---|---|
| 740 | 3.625 | 0 |
| 741 | 3.625 | 0 |
| 742 | 3.625 | 0 |
| 743 | 3.625 | 0 |
| 744 | 3.625 | 0 |
| 745 | 3.625 | 0 |
| 746 | 3.625 | 0 |
| 747 | 3.625 | 0 |
| 748 | 3.625 | 0 |
| 749 | 3.625 | 0 |
| 750 | 3.625 | 0 |
| 751 | 3.625 | 0 |
| 752 | 3.625 | 0 |
| 753 | 3.625 | 0 |
| 754 | 3.625 | 0 |
| 755 | 3.625 | 0 |
| 756 | 3.625 | 0 |
| 757 | 3.625 | 0 |
| 758 | 3.625 | 0 |
| 759 | 3.625 | 0 |
| 760 | 3.625 | 0 |
| 761 | 3.625 | 0 |
| 762 | 3.625 | 0 |
| 763 | 3.625 | 0 |
| 764 | 3.625 | 0 |
| 765 | 3.625 | 0 |
| 766 | 3.875 | 0 |
| 767 | 3.875 | 0 |
| 768 | 3.875 | 0 |
| 769 | 3.875 | 0 |
| 770 | 3.875 | 0 |
| 771 | 3.875 | 0 |
| 772 | 3.875 | 0 |
| 773 | 3.875 | 0 |
| 774 | 3.875 | 0 |
| 775 | 3.875 | 0 |
| 776 | 3.875 | 0 |
| 777 | 3.875 | 0 |
| 778 | 3.875 | 0 |
| 779 | 3.875 | 0 |
| 780 | 3.875 | 0 |
| 781 | 3.875 | 0 |
| 782 | 3.875 | 0 |
| 783 | 3.875 | 0 |
| 784 | 3.875 | 0 |
| 785 | 3.875 | 0 |
| 786 | 3.875 | 0 |
| 787 | 3.875 | 0 |
| 788 | 3.875 | 0 |
| 789 | 3.875 | 0 |
| 790 | 3.875 | 0 |
| 791 | 3.875 | 0 |
| 792 | 3.875 | 0 |
| 793 | 3.875 | 0 |
| 794 | 3.875 | 0 |
| 795 | 3.875 | 0 |
| 796 | 3.875 | 0 |
| 797 | 3.875 | 0 |
| 798 | 4.125 | 0 |
| 799 | 4.125 | 0 |
| 800 | 4.125 | 0 |
| 801 | 4.125 | 0 |
| 802 | 4.125 | 0 |
| 803 | 4.125 | 0 |
| 804 | 4.125 | 0 |
| 805 | 4.125 | 0 |
| 806 | 4.125 | 0 |
| 807 | 4.125 | 0 |
| 808 | 4.125 | 0 |
| 809 | 4.125 | 0 |
| 810 | 4.125 | 0 |
| 811 | 4.125 | 0 |
| 812 | 4.125 | 0 |
| 813 | 4.125 | 0 |
| 814 | 4.125 | 0 |
| 815 | 4.125 | 0 |
| 816 | 4.125 | 0 |
| 817 | 4.125 | 0 |
| 818 | 4.125 | 0 |
| 819 | 4.125 | 0 |
| 820 | 4.125 | 0 |
| 821 | 4.125 | 0 |
| 822 | 4.125 | 0 |
| 823 | 4.125 | 0 |
| 824 | 4.375 | 0 |
| 825 | 4.375 | 0 |
| 826 | 4.375 | 0 |
| 827 | 4.375 | 0 |
| 828 | 4.375 | 0 |
| 829 | 4.375 | 0 |
| 830 | 4.375 | 0 |
| 831 | 4.375 | 0 |
| 832 | 4.375 | 0 |

| | | |
|---|---|---|
| 833 | 4.125 | 0 |
| 834 | 4.125 | 0 |
| 835 | 4.125 | 0 |
| 836 | 4.125 | 0 |
| 837 | 4.125 | 0 |
| 838 | 4.125 | 0 |
| 839 | 4.125 | 0 |
| 840 | 4.125 | 0 |
| 841 | 4.125 | 0 |
| 842 | 4.125 | 0 |
| 843 | 4.125 | 0 |
| 844 | 4.125 | 0 |
| 845 | 4.125 | 0 |
| 846 | 4.125 | 0 |
| 847 | 4.125 | 0 |
| 848 | 4.125 | 0 |
| 849 | 4.125 | 0 |
| 850 | 4.125 | 0 |
| 851 | 4.125 | 0 |
| 852 | 4.125 | 0 |
| 853 | 4.125 | 0 |
| 854 | 4.125 | 0 |
| 855 | 4.375 | 0 |
| 856 | 4.375 | 0 |
| 857 | 4.375 | 0 |
| 858 | 4.375 | 0 |
| 859 | 4.375 | 0 |
| 860 | 4.375 | 0 |
| 861 | 4.375 | 0 |
| 862 | 4.375 | 0 |
| 863 | 4.375 | 0 |
| 864 | 4.375 | 0 |
| 865 | 4.375 | 0 |
| 866 | 4.375 | 0 |
| 867 | 4.375 | 0 |
| 868 | 4.375 | 0 |
| 869 | 4.375 | 0 |
| 870 | 4.375 | 0 |
| 871 | 4.375 | 0 |
| 872 | 4.375 | 0 |
| 873 | 4.375 | 0 |
| 874 | 4.375 | 0 |
| 875 | 4.375 | 0 |
| 876 | 4.375 | 0 |
| 877 | 4.375 | 0 |
| 878 | 4.375 | 0 |
| 879 | 4.375 | 0 |
| 880 | 4.375 | 0 |
| 881 | 4.375 | 0 |
| 882 | 4.375 | 0 |
| 883 | 4.375 | 0 |
| 884 | 4.375 | 0 |
| 885 | 4.625 | 0 |
| 886 | 4.625 | 0 |
| 887 | 4.625 | 0 |
| 888 | 4.625 | 0 |
| 889 | 4.625 | 0 |
| 890 | 4.625 | 0 |
| 891 | 4.625 | 0 |
| 892 | 4.625 | 0 |
| 893 | 4.625 | 0 |
| 894 | 4.625 | 0 |
| 895 | 4.625 | 0 |
| 896 | 4.625 | 0 |
| 897 | 4.625 | 0 |
| 898 | 4.625 | 0 |
| 899 | 4.625 | 0 |
| 900 | 4.625 | 0 |
| 901 | 4.625 | 0 |
| 902 | 4.625 | 0 |
| 903 | 4.625 | 0 |
| 904 | 4.625 | 0 |
| 905 | 4.625 | 0 |
| 906 | 4.625 | 0 |
| 907 | 4.625 | 0 |
| 908 | 4.625 | 0 |
| 909 | 4.625 | 0 |
| 910 | 4.625 | 0 |
| 911 | 4.625 | 0 |
| 912 | 4.625 | 0 |
| 913 | 4.625 | 0 |
| 914 | 4.625 | 0 |
| 915 | 4.875 | 0 |
| 916 | 4.875 | 0 |
| 917 | 4.875 | 0 |
| 918 | 4.875 | 0 |
| 919 | 4.875 | 0 |
| 920 | 4.875 | 0 |
| 921 | 4.875 | 0 |
| 922 | 4.875 | 0 |
| 923 | 4.875 | 0 |
| 924 | 4.875 | 0 |
| 925 | 4.875 | 0 |

| | | |
|---|---|---|
| 833 | 4.375 | 0 |
| 834 | 4.375 | 0 |
| 835 | 4.375 | 0 |
| 836 | 4.375 | 0 |
| 837 | 4.375 | 0 |
| 838 | 4.375 | 0 |
| 839 | 4.375 | 0 |
| 840 | 4.375 | 0 |
| 841 | 4.375 | 0 |
| 842 | 4.375 | 0 |
| 843 | 4.375 | 0 |
| 844 | 4.375 | 0 |
| 845 | 4.375 | 0 |
| 846 | 4.375 | 0 |
| 847 | 4.375 | 0 |
| 848 | 4.375 | 0 |
| 849 | 4.375 | 0 |
| 850 | 4.625 | 0 |
| 851 | 4.625 | 0 |
| 852 | 4.625 | 0 |
| 853 | 4.625 | 0 |
| 854 | 4.625 | 0 |
| 855 | 4.625 | 0 |
| 856 | 4.625 | 0 |
| 857 | 4.625 | 0 |
| 858 | 4.625 | 0 |
| 859 | 4.625 | 0 |
| 860 | 4.625 | 0 |
| 861 | 4.625 | 0 |
| 862 | 4.625 | 0 |
| 863 | 4.625 | 0 |
| 864 | 4.625 | 0 |
| 865 | 4.625 | 0 |
| 866 | 4.625 | 0 |
| 867 | 4.625 | 0 |
| 868 | 4.625 | 0 |
| 869 | 4.625 | 0 |
| 870 | 4.625 | 0 |
| 871 | 4.625 | 0 |
| 872 | 4.625 | 0 |
| 873 | 4.625 | 0 |
| 874 | 4.625 | 0 |
| 875 | 4.625 | 0 |
| 876 | 4.875 | 0 |
| 877 | 4.875 | 0 |
| 878 | 4.875 | 0 |
| 879 | 4.875 | 0 |
| 880 | 4.875 | 0 |
| 881 | 4.875 | 0 |
| 882 | 4.875 | 0 |
| 883 | 4.875 | 0 |
| 884 | 4.875 | 0 |
| 885 | 4.875 | 0 |
| 886 | 4.875 | 0 |
| 887 | 4.875 | 0 |
| 888 | 4.875 | 0 |
| 889 | 4.875 | 0 |
| 890 | 4.875 | 0 |
| 891 | 4.875 | 0 |
| 892 | 4.875 | 0 |
| 893 | 4.875 | 0 |
| 894 | 4.875 | 0 |
| 895 | 4.875 | 0 |
| 896 | 4.875 | 0 |
| 897 | 4.875 | 0 |
| 898 | 4.875 | 0 |
| 899 | 4.875 | 0 |
| 900 | 4.875 | 0 |
| 901 | 4.875 | 0 |
| 902 | 5.125 | 0 |
| 903 | 5.125 | 0 |
| 904 | 5.125 | 0 |
| 905 | 5.125 | 0 |
| 906 | 5.125 | 0 |
| 907 | 5.125 | 0 |
| 908 | 5.125 | 0 |
| 909 | 5.125 | 0 |
| 910 | 5.125 | 0 |
| 911 | 5.125 | 0 |
| 912 | 5.125 | 0 |
| 913 | 5.125 | 0 |
| 914 | 5.125 | 0 |
| 915 | 5.125 | 0 |
| 916 | 5.125 | 0 |
| 917 | 5.125 | 0 |
| 918 | 5.125 | 0 |
| 919 | 5.125 | 0 |
| 920 | 5.125 | 0 |
| 921 | 5.125 | 0 |
| 922 | 5.125 | 0 |
| 923 | 5.125 | 0 |
| 924 | 5.125 | 0 |
| 925 | 5.125 | 0 |

| | | |
|---|---|---|
| 926 | 4.875 | 0 |
| 927 | 4.875 | 0 |
| 928 | 4.875 | 0 |
| 929 | 4.875 | 0 |
| 930 | 4.875 | 0 |
| 931 | 4.875 | 0 |
| 932 | 4.875 | 0 |
| 933 | 4.875 | 0 |
| 934 | 4.875 | 0 |
| 935 | 4.875 | 0 |
| 936 | 4.875 | 0 |
| 937 | 4.875 | 0 |
| 938 | 4.875 | 0 |
| 939 | 4.875 | 0 |
| 940 | 4.875 | 0 |
| 941 | 4.875 | 0 |
| 942 | 4.875 | 0 |
| 943 | 4.875 | 0 |
| 944 | 4.875 | 0 |
| 945 | 5.125 | 0 |
| 946 | 5.125 | 0 |
| 947 | 5.125 | 0 |
| 948 | 5.125 | 0 |
| 949 | 5.125 | 0 |
| 950 | 5.125 | 0 |
| 951 | 5.125 | 0 |
| 952 | 5.125 | 0 |
| 953 | 5.125 | 0 |
| 954 | 5.125 | 0 |
| 955 | 5.125 | 0 |
| 956 | 5.125 | 0 |
| 957 | 5.125 | 0 |
| 958 | 5.125 | 0 |
| 959 | 5.125 | 0 |
| 960 | 5.125 | 0 |
| 961 | 5.125 | 0 |
| 962 | 5.125 | 0 |
| 963 | 5.125 | 0 |
| 964 | 5.125 | 0 |
| 965 | 5.125 | 0 |
| 966 | 5.125 | 0 |
| 967 | 5.125 | 0 |
| 968 | 5.125 | 0 |
| 969 | 5.125 | 0 |
| 970 | 5.125 | 0 |
| 971 | 5.125 | 0 |
| 972 | 5.125 | 0 |
| 973 | 5.125 | 0 |
| 974 | 5.125 | 0 |
| 975 | 5.125 | 0 |
| 976 | 5.125 | 0 |
| 977 | 5.125 | 0 |
| 978 | 5.125 | 0 |
| 979 | 5.125 | 0 |
| 980 | 5.125 | 0 |
| 981 | 5.375 | 0 |
| 982 | 5.375 | 0 |
| 983 | 5.375 | 0 |
| 984 | 5.375 | 0 |
| 985 | 5.375 | 0 |
| 986 | 5.375 | 0 |
| 987 | 5.375 | 0 |
| 988 | 5.375 | 0 |
| 989 | 5.375 | 0 |
| 990 | 5.375 | 0 |
| 991 | 5.375 | 0 |
| 992 | 5.375 | 0 |
| 993 | 5.375 | 0 |
| 994 | 5.375 | 0 |
| 995 | 5.375 | 0 |
| 996 | 5.375 | 0 |
| 997 | 5.375 | 0 |
| 998 | 5.375 | 0 |
| 999 | 5.375 | 0 |
| 1000 | 5.375 | 0 |
| 1001 | 5.375 | 0 |
| 1002 | 5.375 | 0 |
| 1003 | 5.375 | 0 |
| 1004 | 5.375 | 0 |
| 1005 | 5.375 | 0 |
| 1006 | 5.375 | 0 |
| 1007 | 5.375 | 0 |
| 1008 | 5.375 | 0 |
| 1009 | 5.375 | 0 |
| 1010 | 5.375 | 0 |
| 1011 | 5.375 | 0 |
| 1012 | 5.375 | 0 |
| 1013 | 5.375 | 0 |
| 1014 | 5.375 | 0 |
| 1015 | 5.375 | 0 |
| 1016 | 5.375 | 0 |
| 1017 | 5.625 | 0 |
| 1018 | 5.625 | 0 |

| | | |
|---|---|---|
| 926 | 5.125 | 0 |
| 927 | 5.125 | 0 |
| 928 | 5.125 | 0 |
| 929 | 5.125 | 0 |
| 930 | 5.125 | 0 |
| 931 | 5.125 | 0 |
| 932 | 5.125 | 0 |
| 933 | 5.125 | 0 |
| 934 | 5.125 | 0 |
| 935 | 5.125 | 0 |
| 936 | 5.125 | 0 |
| 937 | 5.125 | 0 |
| 938 | 5.125 | 0 |
| 939 | 5.375 | 0 |
| 940 | 5.375 | 0 |
| 941 | 5.375 | 0 |
| 942 | 5.375 | 0 |
| 943 | 5.375 | 0 |
| 944 | 5.375 | 0 |
| 945 | 5.375 | 0 |
| 946 | 5.375 | 0 |
| 947 | 5.375 | 0 |
| 948 | 5.375 | 0 |
| 949 | 5.375 | 0 |
| 950 | 5.375 | 0 |
| 951 | 5.375 | 0 |
| 952 | 5.375 | 0 |
| 953 | 5.375 | 0 |
| 954 | 5.375 | 0 |
| 955 | 5.375 | 0 |
| 956 | 5.375 | 0 |
| 957 | 5.375 | 0 |
| 958 | 5.375 | 0 |
| 959 | 5.375 | 0 |
| 960 | 5.375 | 0 |
| 961 | 5.375 | 0 |
| 962 | 5.375 | 0 |
| 963 | 5.375 | 0 |
| 964 | 5.375 | 0 |
| 965 | 5.375 | 0 |
| 966 | 5.375 | 0 |
| 967 | 5.375 | 0 |
| 968 | 5.375 | 0 |
| 969 | 5.375 | 0 |
| 970 | 5.375 | 0 |
| 971 | 5.375 | 0 |
| 972 | 5.375 | 0 |
| 973 | 5.375 | 0 |
| 974 | 5.375 | 0 |
| 975 | 5.375 | 0 |
| 976 | 5.625 | 0 |
| 977 | 5.625 | 0 |
| 978 | 5.625 | 0 |
| 979 | 5.625 | 0 |
| 980 | 5.625 | 0 |
| 981 | 5.625 | 0 |
| 982 | 5.625 | 0 |
| 983 | 5.625 | 0 |
| 984 | 5.625 | 0 |
| 985 | 5.625 | 0 |
| 986 | 5.625 | 0 |
| 987 | 5.625 | 0 |
| 988 | 5.625 | 0 |
| 989 | 5.625 | 0 |
| 990 | 5.625 | 0 |
| 991 | 5.625 | 0 |
| 992 | 5.625 | 0 |
| 993 | 5.625 | 0 |
| 994 | 5.625 | 0 |
| 995 | 5.625 | 0 |
| 996 | 5.625 | 0 |
| 997 | 5.625 | 0 |
| 998 | 5.625 | 0 |
| 999 | 5.625 | 0 |
| 1000 | 5.625 | 0 |
| 1001 | 5.625 | 0 |
| 1002 | 5.625 | 0 |
| 1003 | 5.625 | 0 |
| 1004 | 5.625 | 0 |
| 1005 | 5.625 | 0 |
| 1006 | 5.625 | 0 |
| 1007 | 5.625 | 0 |
| 1008 | 5.625 | 0 |
| 1009 | 5.625 | 0 |
| 1010 | 5.625 | 0 |
| 1011 | 5.625 | 0 |
| 1012 | 5.625 | 0 |
| 1013 | 5.875 | 0 |
| 1014 | 5.875 | 0 |
| 1015 | 5.875 | 0 |
| 1016 | 5.875 | 0 |
| 1017 | 5.875 | 0 |
| 1018 | 5.875 | 0 |

| | | |
|---|---|---|
| 1019 | 5.625 | 0 |
| 1020 | 5.625 | 0 |
| 1021 | 5.625 | 0 |
| 1022 | 5.625 | 0 |
| 1023 | 5.625 | 0 |
| 1024 | 5.625 | 0 |
| 1025 | 5.625 | 0 |
| 1026 | 5.625 | 0 |
| 1027 | 5.625 | 0 |
| 1028 | 5.625 | 0 |
| 1029 | 5.625 | 0 |
| 1030 | 5.625 | 0 |
| 1031 | 5.625 | 0 |
| 1032 | 5.625 | 0 |
| 1033 | 5.625 | 0 |
| 1034 | 5.625 | 0 |
| 1035 | 5.625 | 0 |
| 1036 | 5.625 | 0 |
| 1037 | 5.625 | 0 |
| 1038 | 5.625 | 0 |
| 1039 | 5.625 | 0 |
| 1040 | 5.625 | 0 |
| 1041 | 5.625 | 0 |
| 1042 | 5.625 | 0 |
| 1043 | 5.625 | 0 |
| 1044 | 5.625 | 0 |
| 1045 | 5.625 | 0 |
| 1046 | 5.625 | 0 |
| 1047 | 5.625 | 0 |
| 1048 | 5.625 | 0 |
| 1049 | 5.625 | 0 |
| 1050 | 5.625 | 0 |
| 1051 | 5.625 | 0 |
| 1052 | 5.625 | 0 |
| 1053 | 5.875 | 0 |
| 1054 | 5.875 | 0 |
| 1055 | 5.875 | 0 |
| 1056 | 5.875 | 0 |
| 1057 | 5.875 | 0 |
| 1058 | 5.875 | 0 |
| 1059 | 5.875 | 0 |
| 1060 | 5.875 | 0 |
| 1061 | 5.875 | 0 |
| 1062 | 5.875 | 0 |
| 1063 | 5.875 | 0 |
| 1064 | 5.875 | 0 |
| 1065 | 5.875 | 0 |
| 1066 | 5.875 | 0 |
| 1067 | 5.875 | 0 |
| 1068 | 5.875 | 0 |
| 1069 | 5.875 | 0 |
| 1070 | 5.875 | 0 |
| 1071 | 5.875 | 0 |
| 1072 | 5.875 | 0 |
| 1073 | 5.875 | 0 |
| 1074 | 5.875 | 0 |
| 1075 | 5.875 | 0 |
| 1076 | 5.875 | 0 |
| 1077 | 5.875 | 0 |
| 1078 | 5.875 | 0 |
| 1079 | 5.875 | 0 |
| 1080 | 5.875 | 0 |
| 1081 | 5.875 | 0 |
| 1082 | 5.875 | 0 |
| 1083 | 5.875 | 0 |
| 1084 | 5.875 | 0 |
| 1085 | 5.875 | 0 |
| 1086 | 5.875 | 0 |
| 1087 | 5.875 | 0 |
| 1088 | 5.875 | 0 |
| 1089 | 6.125 | 0 |
| 1090 | 6.125 | 0 |
| 1091 | 6.125 | 0 |
| 1092 | 6.125 | 0 |
| 1093 | 6.125 | 0 |
| 1094 | 6.125 | 0 |
| 1095 | 6.125 | 0 |
| 1096 | 6.125 | 0 |
| 1097 | 6.125 | 0 |
| 1098 | 6.125 | 0 |
| 1099 | 6.125 | 0 |
| 1100 | 6.125 | 0 |
| 1101 | 6.125 | 0 |
| 1102 | 6.125 | 0 |
| 1103 | 6.125 | 0 |
| 1104 | 6.125 | 0 |
| 1105 | 6.125 | 0 |
| 1106 | 6.125 | 0 |
| 1107 | 6.125 | 0 |
| 1108 | 6.125 | 0 |
| 1109 | 6.125 | 0 |
| 1110 | 6.125 | 0 |
| 1111 | 6.125 | 0 |

| | | |
|---|---|---|
| 1019 | 5.875 | 0 |
| 1020 | 5.875 | 0 |
| 1021 | 5.875 | 0 |
| 1022 | 5.875 | 0 |
| 1023 | 5.875 | 0 |
| 1024 | 5.875 | 0 |
| 1025 | 5.875 | 0 |
| 1026 | 5.875 | 0 |
| 1027 | 5.875 | 0 |
| 1028 | 5.875 | 0 |
| 1029 | 5.875 | 0 |
| 1030 | 5.875 | 0 |
| 1031 | 5.875 | 0 |
| 1032 | 5.875 | 0 |
| 1033 | 5.875 | 0 |
| 1034 | 5.875 | 0 |
| 1035 | 5.875 | 0 |
| 1036 | 5.875 | 0 |
| 1037 | 5.875 | 0 |
| 1038 | 5.875 | 0 |
| 1039 | 5.875 | 0 |
| 1040 | 5.875 | 0 |
| 1041 | 5.875 | 0 |
| 1042 | 5.875 | 0 |
| 1043 | 5.875 | 0 |
| 1044 | 5.875 | 0 |
| 1045 | 5.875 | 0 |
| 1046 | 5.875 | 0 |
| 1047 | 5.875 | 0 |
| 1048 | 5.875 | 0 |
| 1049 | 5.875 | 0 |
| 1050 | 6.125 | 0 |
| 1051 | 6.125 | 0 |
| 1052 | 6.125 | 0 |
| 1053 | 6.125 | 0 |
| 1054 | 6.125 | 0 |
| 1055 | 6.125 | 0 |
| 1056 | 6.125 | 0 |
| 1057 | 6.125 | 0 |
| 1058 | 6.125 | 0 |
| 1059 | 6.125 | 0 |
| 1060 | 6.125 | 0 |
| 1061 | 6.125 | 0 |
| 1062 | 6.125 | 0 |
| 1063 | 6.125 | 0 |
| 1064 | 6.125 | 0 |
| 1065 | 6.125 | 0 |
| 1066 | 6.125 | 0 |
| 1067 | 6.125 | 0 |
| 1068 | 6.125 | 0 |
| 1069 | 6.125 | 0 |
| 1070 | 6.125 | 0 |
| 1071 | 6.125 | 0 |
| 1072 | 6.125 | 0 |
| 1073 | 6.125 | 0 |
| 1074 | 6.125 | 0 |
| 1075 | 6.125 | 0 |
| 1076 | 6.125 | 0 |
| 1077 | 6.125 | 0 |
| 1078 | 6.125 | 0 |
| 1079 | 6.125 | 0 |
| 1080 | 6.125 | 0 |
| 1081 | 6.125 | 0 |
| 1082 | 6.125 | 0 |
| 1083 | 6.125 | 0 |
| 1084 | 6.125 | 0 |
| 1085 | 6.125 | 0 |
| 1086 | 6.125 | 0 |
| 1087 | 6.125 | 0 |
| 1088 | 6.375 | 0 |
| 1089 | 6.375 | 0 |
| 1090 | 6.375 | 0 |
| 1091 | 6.375 | 0 |
| 1092 | 6.375 | 0 |
| 1093 | 6.375 | 0 |
| 1094 | 6.375 | 0 |
| 1095 | 6.375 | 0 |
| 1096 | 6.375 | 0 |
| 1097 | 6.375 | 0 |
| 1098 | 6.375 | 0 |
| 1099 | 6.375 | 0 |
| 1100 | 6.375 | 0 |
| 1101 | 6.375 | 0 |
| 1102 | 6.375 | 0 |
| 1103 | 6.375 | 0 |
| 1104 | 6.375 | 0 |
| 1105 | 6.375 | 0 |
| 1106 | 6.375 | 0 |
| 1107 | 6.375 | 0 |
| 1108 | 6.375 | 0 |
| 1109 | 6.375 | 0 |
| 1110 | 6.375 | 0 |
| 1111 | 6.375 | 0 |

| | | |
|---|---|---|
| 1112 | 6.125 | 0 |
| 1113 | 6.125 | 0 |
| 1114 | 6.125 | 0 |
| 1115 | 6.125 | 0 |
| 1116 | 6.125 | 0 |
| 1117 | 6.125 | 0 |
| 1118 | 6.125 | 0 |
| 1119 | 6.125 | 0 |
| 1120 | 6.375 | 0 |
| 1121 | 6.375 | 0 |
| 1122 | 6.375 | 0 |
| 1123 | 6.375 | 0 |
| 1124 | 6.375 | 0 |
| 1125 | 6.375 | 0 |
| 1126 | 6.375 | 0 |
| 1127 | 6.375 | 0 |
| 1128 | 6.375 | 0 |
| 1129 | 6.375 | 0 |
| 1130 | 6.375 | 0 |
| 1131 | 6.375 | 0 |
| 1132 | 6.375 | 0 |
| 1133 | 6.375 | 0 |
| 1134 | 6.375 | 0 |
| 1135 | 6.375 | 0 |
| 1136 | 6.375 | 0 |
| 1137 | 6.375 | 0 |
| 1138 | 6.375 | 0 |
| 1139 | 6.375 | 0 |
| 1140 | 6.375 | 0 |
| 1141 | 6.375 | 0 |
| 1142 | 6.375 | 0 |
| 1143 | 6.375 | 0 |
| 1144 | 6.375 | 0 |
| 1145 | 6.375 | 0 |
| 1146 | 6.375 | 0 |
| 1147 | 6.375 | 0 |
| 1148 | 6.375 | 0 |
| 1149 | 6.375 | 0 |
| 1150 | 6.375 | 0 |
| 1151 | 6.625 | 0 |
| 1152 | 6.625 | 0 |
| 1153 | 6.625 | 0 |
| 1154 | 6.625 | 0 |
| 1155 | 6.625 | 0 |
| 1156 | 6.625 | 0 |
| 1157 | 6.625 | 0 |
| 1158 | 6.625 | 0 |
| 1159 | 6.625 | 0 |
| 1160 | 6.625 | 0 |
| 1161 | 6.625 | 0 |
| 1162 | 6.625 | 0 |
| 1163 | 6.625 | 0 |
| 1164 | 6.625 | 0 |
| 1165 | 6.625 | 0 |
| 1166 | 6.625 | 0 |
| 1167 | 6.625 | 0 |
| 1168 | 6.625 | 0 |
| 1169 | 6.625 | 0 |
| 1170 | 6.625 | 0 |
| 1171 | 6.625 | 0 |
| 1172 | 6.625 | 0 |
| 1173 | 6.625 | 0 |
| 1174 | 6.625 | 0 |
| 1175 | 6.625 | 0 |
| 1176 | 6.625 | 0 |
| 1177 | 6.625 | 0 |
| 1178 | 6.625 | 0 |
| 1179 | 6.625 | 0 |
| 1180 | 6.625 | 0 |
| 1181 | 6.625 | 0 |
| 1182 | 6.875 | 0 |
| 1183 | 6.875 | 0 |
| 1184 | 6.875 | 0 |
| 1185 | 6.875 | 0 |
| 1186 | 6.875 | 0 |
| 1187 | 6.875 | 0 |
| 1188 | 6.875 | 0 |
| 1189 | 6.875 | 0 |
| 1190 | 6.875 | 0 |
| 1191 | 6.875 | 0 |
| 1192 | 6.875 | 0 |
| 1193 | 6.875 | 0 |
| 1194 | 6.875 | 0 |
| 1195 | 6.875 | 0 |
| 1196 | 6.875 | 0 |
| 1197 | 6.875 | 0 |
| 1198 | 6.875 | 0 |
| 1199 | 6.875 | 0 |
| 1200 | 6.875 | 0 |
| 1201 | 6.875 | 0 |
| 1202 | 6.875 | 0 |
| 1203 | 6.875 | 0 |
| 1204 | 6.875 | 0 |

| | | |
|---|---|---|
| 1112 | 6.375 | 0 |
| 1113 | 6.375 | 0 |
| 1114 | 6.375 | 0 |
| 1115 | 6.375 | 0 |
| 1116 | 6.375 | 0 |
| 1117 | 6.375 | 0 |
| 1118 | 6.375 | 0 |
| 1119 | 6.375 | 0 |
| 1120 | 6.375 | 0 |
| 1121 | 6.375 | 0 |
| 1122 | 6.375 | 0 |
| 1123 | 6.375 | 0 |
| 1124 | 6.375 | 0 |
| 1125 | 6.375 | 0 |
| 1126 | 6.625 | 0 |
| 1127 | 6.625 | 0 |
| 1128 | 6.625 | 0 |
| 1129 | 6.625 | 0 |
| 1130 | 6.625 | 0 |
| 1131 | 6.625 | 0 |
| 1132 | 6.625 | 0 |
| 1133 | 6.625 | 0 |
| 1134 | 6.625 | 0 |
| 1135 | 6.625 | 0 |
| 1136 | 6.625 | 0 |
| 1137 | 6.625 | 0 |
| 1138 | 6.625 | 0 |
| 1139 | 6.625 | 0 |
| 1140 | 6.625 | 0 |
| 1141 | 6.625 | 0 |
| 1142 | 6.625 | 0 |
| 1143 | 6.625 | 0 |
| 1144 | 6.625 | 0 |
| 1145 | 6.625 | 0 |
| 1146 | 6.625 | 0 |
| 1147 | 6.625 | 0 |
| 1148 | 6.625 | 0 |
| 1149 | 6.625 | 0 |
| 1150 | 6.625 | 0 |
| 1151 | 6.625 | 0 |
| 1152 | 6.625 | 0 |
| 1153 | 6.625 | 0 |
| 1154 | 6.625 | 0 |
| 1155 | 6.625 | 0 |
| 1156 | 6.625 | 0 |
| 1157 | 6.625 | 0 |
| 1158 | 6.625 | 0 |
| 1159 | 6.625 | 0 |
| 1160 | 6.625 | 0 |
| 1161 | 6.625 | 0 |
| 1162 | 6.625 | 0 |
| 1163 | 6.625 | 0 |
| 1164 | 6.875 | 0 |
| 1165 | 6.875 | 0 |
| 1166 | 6.875 | 0 |
| 1167 | 6.875 | 0 |
| 1168 | 6.875 | 0 |
| 1169 | 6.875 | 0 |
| 1170 | 6.875 | 0 |
| 1171 | 6.875 | 0 |
| 1172 | 6.875 | 0 |
| 1173 | 6.875 | 0 |
| 1174 | 6.875 | 0 |
| 1175 | 6.875 | 0 |
| 1176 | 6.875 | 0 |
| 1177 | 6.875 | 0 |
| 1178 | 6.875 | 0 |
| 1179 | 6.875 | 0 |
| 1180 | 6.875 | 0 |
| 1181 | 6.875 | 0 |
| 1182 | 6.875 | 0 |
| 1183 | 6.875 | 0 |
| 1184 | 6.875 | 0 |
| 1185 | 6.875 | 0 |
| 1186 | 6.875 | 0 |
| 1187 | 6.875 | 0 |
| 1188 | 6.875 | 0 |
| 1189 | 6.875 | 0 |
| 1190 | 6.875 | 0 |
| 1191 | 6.875 | 0 |
| 1192 | 6.875 | 0 |
| 1193 | 6.875 | 0 |
| 1194 | 6.875 | 0 |
| 1195 | 6.875 | 0 |
| 1196 | 6.875 | 0 |
| 1197 | 6.875 | 0 |
| 1198 | 6.875 | 0 |
| 1199 | 6.875 | 0 |
| 1200 | 6.875 | 0 |
| 1201 | 6.875 | 0 |
| 1202 | 7.125 | 0 |
| 1203 | 7.125 | 0 |
| 1204 | 7.125 | 0 |

| | | |
|---|---|---|
| 1205 | 6.875 | 0 |
| 1206 | 6.875 | 0 |
| 1207 | 6.875 | 0 |
| 1208 | 6.875 | 0 |
| 1209 | 6.875 | 0 |
| 1210 | 6.875 | 0 |
| 1211 | 6.875 | 0 |
| 1212 | 6.875 | 0 |
| 1213 | 7.125 | 0 |
| 1214 | 7.125 | 0 |
| 1215 | 7.125 | 0 |
| 1216 | 7.125 | 0 |
| 1217 | 7.125 | 0 |
| 1218 | 7.125 | 0 |
| 1219 | 7.125 | 0 |
| 1220 | 7.125 | 0 |
| 1221 | 7.125 | 0 |
| 1222 | 7.125 | 0 |
| 1223 | 7.125 | 0 |
| 1224 | 7.125 | 0 |
| 1225 | 7.125 | 0 |
| 1226 | 7.125 | 0 |
| 1227 | 7.125 | 0 |
| 1228 | 7.125 | 0 |
| 1229 | 7.125 | 0 |
| 1230 | 7.125 | 0 |
| 1231 | 7.125 | 0 |
| 1232 | 7.125 | 0 |
| 1233 | 7.125 | 0 |
| 1234 | 7.125 | 0 |
| 1235 | 7.125 | 0 |
| 1236 | 7.125 | 0 |
| 1237 | 7.125 | 0 |
| 1238 | 7.125 | 0 |
| 1239 | 7.125 | 0 |
| 1240 | 7.125 | 0 |
| 1241 | 7.125 | 0 |
| 1242 | 7.125 | 0 |
| 1243 | 7.125 | 0 |
| 1244 | 7.375 | 0 |
| 1245 | 7.375 | 0 |
| 1246 | 7.375 | 0 |
| 1247 | 7.375 | 0 |
| 1248 | 7.375 | 0 |
| 1249 | 7.375 | 0 |
| 1250 | 7.375 | 0 |
| 1251 | 7.375 | 0 |
| 1252 | 7.375 | 0 |
| 1253 | 7.375 | 0 |
| 1254 | 7.375 | 0 |
| 1255 | 7.375 | 0 |
| 1256 | 7.375 | 0 |
| 1257 | 7.375 | 0 |
| 1258 | 7.375 | 0 |
| 1259 | 7.375 | 0 |
| 1260 | 7.375 | 0 |
| 1261 | 7.375 | 0 |
| 1262 | 7.375 | 0 |
| 1263 | 7.375 | 0 |
| 1264 | 7.375 | 0 |
| 1265 | 7.375 | 0 |
| 1266 | 7.375 | 0 |
| 1267 | 7.375 | 0 |
| 1268 | 7.375 | 0 |
| 1269 | 7.375 | 0 |
| 1270 | 7.375 | 0 |
| 1271 | 7.375 | 0 |
| 1272 | 7.375 | 0 |
| 1273 | 7.375 | 0 |
| 1274 | 7.375 | 0 |
| 1275 | 7.625 | 0 |
| 1276 | 7.625 | 0 |
| 1277 | 7.625 | 0 |
| 1278 | 7.625 | 0 |
| 1279 | 7.625 | 0 |
| 1280 | 7.625 | 0 |
| 1281 | 7.625 | 0 |
| 1282 | 7.625 | 0 |
| 1283 | 7.625 | 0 |
| 1284 | 7.625 | 0 |
| 1285 | 7.625 | 0 |
| 1286 | 7.625 | 0 |
| 1287 | 7.625 | 0 |
| 1288 | 7.625 | 0 |
| 1289 | 7.625 | 0 |
| 1290 | 7.625 | 0 |
| 1291 | 7.625 | 0 |
| 1292 | 7.625 | 0 |
| 1293 | 7.625 | 0 |
| 1294 | 7.625 | 0 |
| 1295 | 7.625 | 0 |
| 1296 | 7.625 | 0 |
| 1297 | 7.625 | 0 |

| | | |
|---|---|---|
| 1205 | 7.125 | 0 |
| 1206 | 7.125 | 0 |
| 1207 | 7.125 | 0 |
| 1208 | 7.125 | 0 |
| 1209 | 7.125 | 0 |
| 1210 | 7.125 | 0 |
| 1211 | 7.125 | 0 |
| 1212 | 7.125 | 0 |
| 1213 | 7.125 | 0 |
| 1214 | 7.125 | 0 |
| 1215 | 7.125 | 0 |
| 1216 | 7.125 | 0 |
| 1217 | 7.125 | 0 |
| 1218 | 7.125 | 0 |
| 1219 | 7.125 | 0 |
| 1220 | 7.125 | 0 |
| 1221 | 7.125 | 0 |
| 1222 | 7.125 | 0 |
| 1223 | 7.125 | 0 |
| 1224 | 7.125 | 0 |
| 1225 | 7.125 | 0 |
| 1226 | 7.125 | 0 |
| 1227 | 7.125 | 0 |
| 1228 | 7.125 | 0 |
| 1229 | 7.125 | 0 |
| 1230 | 7.125 | 0 |
| 1231 | 7.125 | 0 |
| 1232 | 7.125 | 0 |
| 1233 | 7.125 | 0 |
| 1234 | 7.125 | 0 |
| 1235 | 7.125 | 0 |
| 1236 | 7.375 | 0 |
| 1237 | 7.375 | 0 |
| 1238 | 7.375 | 0 |
| 1239 | 7.375 | 0 |
| 1240 | 7.375 | 0 |
| 1241 | 7.375 | 0 |
| 1242 | 7.375 | 0 |
| 1243 | 7.375 | 0 |
| 1244 | 7.375 | 0 |
| 1245 | 7.375 | 0 |
| 1246 | 7.375 | 0 |
| 1247 | 7.375 | 0 |
| 1248 | 7.375 | 0 |
| 1249 | 7.375 | 0 |
| 1250 | 7.375 | 0 |
| 1251 | 7.375 | 0 |
| 1252 | 7.375 | 0 |
| 1253 | 7.375 | 0 |
| 1254 | 7.375 | 0 |
| 1255 | 7.375 | 0 |
| 1256 | 7.375 | 0 |
| 1257 | 7.375 | 0 |
| 1258 | 7.375 | 0 |
| 1259 | 7.375 | 0 |
| 1260 | 7.375 | 0 |
| 1261 | 7.375 | 0 |
| 1262 | 7.375 | 0 |
| 1263 | 7.375 | 0 |
| 1264 | 7.375 | 0 |
| 1265 | 7.375 | 0 |
| 1266 | 7.375 | 0 |
| 1267 | 7.375 | 0 |
| 1268 | 7.375 | 0 |
| 1269 | 7.375 | 0 |
| 1270 | 7.625 | 0 |
| 1271 | 7.625 | 0 |
| 1272 | 7.625 | 0 |
| 1273 | 7.625 | 0 |
| 1274 | 7.625 | 0 |
| 1275 | 7.625 | 0 |
| 1276 | 7.625 | 0 |
| 1277 | 7.625 | 0 |
| 1278 | 7.625 | 0 |
| 1279 | 7.625 | 0 |
| 1280 | 7.625 | 0 |
| 1281 | 7.625 | 0 |
| 1282 | 7.625 | 0 |
| 1283 | 7.625 | 0 |
| 1284 | 7.625 | 0 |
| 1285 | 7.625 | 0 |
| 1286 | 7.625 | 0 |
| 1287 | 7.625 | 0 |
| 1288 | 7.625 | 0 |
| 1289 | 7.625 | 0 |
| 1290 | 7.625 | 0 |
| 1291 | 7.625 | 0 |
| 1292 | 7.625 | 0 |
| 1293 | 7.625 | 0 |
| 1294 | 7.625 | 0 |
| 1295 | 7.625 | 0 |
| 1296 | 7.625 | 0 |
| 1297 | 7.625 | 0 |

| | | |
|---|---|---|
| 1298 | 7.625 | 0 |
| 1299 | 7.625 | 0 |
| 1300 | 7.625 | 0 |
| 1301 | 7.625 | 0 |
| 1302 | 7.625 | 0 |
| 1303 | 7.625 | 0 |
| 1304 | 7.625 | 0 |
| 1305 | 7.625 | 0 |
| 1306 | 7.875 | 0 |
| 1307 | 7.875 | 0 |
| 1308 | 7.875 | 0 |
| 1309 | 7.875 | 0 |
| 1310 | 7.875 | 0 |
| 1311 | 7.875 | 0 |
| 1312 | 7.875 | 0 |
| 1313 | 7.875 | 0 |
| 1314 | 7.875 | 0 |
| 1315 | 7.875 | 0 |
| 1316 | 7.875 | 0 |
| 1317 | 7.875 | 0 |
| 1318 | 7.875 | 0 |
| 1319 | 7.875 | 0 |
| 1320 | 7.875 | 0 |
| 1321 | 7.875 | 0 |
| 1322 | 7.875 | 0 |
| 1323 | 7.875 | 0 |
| 1324 | 7.875 | 0 |
| 1325 | 7.875 | 0 |
| 1326 | 7.875 | 0 |
| 1327 | 7.875 | 0 |
| 1328 | 7.875 | 0 |
| 1329 | 7.875 | 0 |
| 1330 | 7.875 | 0 |
| 1331 | 7.875 | 0 |
| 1332 | 7.875 | 0 |
| 1333 | 7.875 | 0 |
| 1334 | 7.875 | 0 |
| 1335 | 7.875 | 0 |
| 1336 | 7.875 | 0 |
| 1337 | 8.125 | 0 |
| 1338 | 8.125 | 0 |
| 1339 | 8.125 | 0 |
| 1340 | 8.125 | 0 |
| 1341 | 8.125 | 0 |
| 1342 | 8.125 | 0 |
| 1343 | 8.125 | 0 |
| 1344 | 8.125 | 0 |
| 1345 | 8.125 | 0 |
| 1346 | 8.125 | 0 |
| 1347 | 8.125 | 0 |
| 1348 | 8.125 | 0 |
| 1349 | 8.125 | 0 |
| 1350 | 8.125 | 0 |
| 1351 | 8.125 | 0 |
| 1352 | 8.125 | 0 |
| 1353 | 8.125 | 0 |
| 1354 | 8.125 | 0 |
| 1355 | 8.125 | 0 |
| 1356 | 8.125 | 0 |
| 1357 | 8.125 | 0 |
| 1358 | 8.125 | 0 |
| 1359 | 8.125 | 0 |
| 1360 | 8.125 | 0 |
| 1361 | 8.125 | 0 |
| 1362 | 8.125 | 0 |
| 1363 | 8.375 | 0 |
| 1364 | 8.375 | 0 |
| 1365 | 8.375 | 0 |
| 1366 | 8.375 | 0 |
| 1367 | 8.375 | 0 |
| 1368 | 8.375 | 0 |
| 1369 | 8.375 | 0 |
| 1370 | 8.375 | 0 |
| 1371 | 8.375 | 0 |
| 1372 | 8.375 | 0 |
| 1373 | 8.375 | 0 |
| 1374 | 8.375 | 0 |
| 1375 | 8.375 | 0 |
| 1376 | 8.375 | 0 |
| 1377 | 8.375 | 0 |
| 1378 | 8.375 | 0 |
| 1379 | 8.375 | 0 |
| 1380 | 8.375 | 0 |
| 1381 | 8.375 | 0 |
| 1382 | 8.375 | 0 |
| 1383 | 8.375 | 0 |
| 1384 | 8.375 | 0 |
| 1385 | 8.375 | 0 |
| 1386 | 8.375 | 0 |
| 1387 | 8.375 | 0 |
| 1388 | 8.375 | 0 |
| 1389 | 8.625 | 0 |
| 1390 | 8.625 | 0 |

| | | |
|---|---|---|
| 1298 | 7.625 | 0 |
| 1299 | 7.625 | 0 |
| 1300 | 7.625 | 0 |
| 1301 | 7.625 | 0 |
| 1302 | 7.625 | 0 |
| 1303 | 7.625 | 0 |
| 1304 | 7.875 | 0 |
| 1305 | 7.875 | 0 |
| 1306 | 7.875 | 0 |
| 1307 | 7.875 | 0 |
| 1308 | 7.875 | 0 |
| 1309 | 7.875 | 0 |
| 1310 | 7.875 | 0 |
| 1311 | 7.875 | 0 |
| 1312 | 7.875 | 0 |
| 1313 | 7.875 | 0 |
| 1314 | 7.875 | 0 |
| 1315 | 7.875 | 0 |
| 1316 | 7.875 | 0 |
| 1317 | 7.875 | 0 |
| 1318 | 7.875 | 0 |
| 1319 | 7.875 | 0 |
| 1320 | 7.875 | 0 |
| 1321 | 7.875 | 0 |
| 1322 | 7.875 | 0 |
| 1323 | 7.875 | 0 |
| 1324 | 7.875 | 0 |
| 1325 | 7.875 | 0 |
| 1326 | 7.875 | 0 |
| 1327 | 7.875 | 0 |
| 1328 | 7.875 | 0 |
| 1329 | 7.875 | 0 |
| 1330 | 7.875 | 0 |
| 1331 | 7.875 | 0 |
| 1332 | 7.875 | 0 |
| 1333 | 7.875 | 0 |
| 1334 | 7.875 | 0 |
| 1335 | 7.875 | 0 |
| 1336 | 7.875 | 0 |
| 1337 | 7.875 | 0 |
| 1338 | 8.125 | 0 |
| 1339 | 8.125 | 0 |
| 1340 | 8.125 | 0 |
| 1341 | 8.125 | 0 |
| 1342 | 8.125 | 0 |
| 1343 | 8.125 | 0 |
| 1344 | 8.125 | 0 |
| 1345 | 8.125 | 0 |
| 1346 | 8.125 | 0 |
| 1347 | 8.125 | 0 |
| 1348 | 8.125 | 0 |
| 1349 | 8.125 | 0 |
| 1350 | 8.125 | 0 |
| 1351 | 8.125 | 0 |
| 1352 | 8.125 | 0 |
| 1353 | 8.125 | 0 |
| 1354 | 8.125 | 0 |
| 1355 | 8.125 | 0 |
| 1356 | 8.125 | 0 |
| 1357 | 8.125 | 0 |
| 1358 | 8.125 | 0 |
| 1359 | 8.125 | 0 |
| 1360 | 8.125 | 0 |
| 1361 | 8.125 | 0 |
| 1362 | 8.125 | 0 |
| 1363 | 8.125 | 0 |
| 1364 | 8.125 | 0 |
| 1365 | 8.375 | 0 |
| 1366 | 8.375 | 0 |
| 1367 | 8.375 | 0 |
| 1368 | 8.375 | 0 |
| 1369 | 8.375 | 0 |
| 1370 | 8.375 | 0 |
| 1371 | 8.375 | 0 |
| 1372 | 8.375 | 0 |
| 1373 | 8.375 | 0 |
| 1374 | 8.375 | 0 |
| 1375 | 8.375 | 0 |
| 1376 | 8.375 | 0 |
| 1377 | 8.375 | 0 |
| 1378 | 8.375 | 0 |
| 1379 | 8.375 | 0 |
| 1380 | 8.375 | 0 |
| 1381 | 8.375 | 0 |
| 1382 | 8.375 | 0 |
| 1383 | 8.375 | 0 |
| 1384 | 8.375 | 0 |
| 1385 | 8.375 | 0 |
| 1386 | 8.375 | 0 |
| 1387 | 8.375 | 0 |
| 1388 | 8.375 | 0 |
| 1389 | 8.375 | 0 |
| 1390 | 8.375 | 0 |

| | | |
|---|---|---|
| 1391 | 8.625 | 0 |
| 1392 | 8.625 | 0 |
| 1393 | 8.625 | 0 |
| 1394 | 8.625 | 0 |
| 1395 | 8.625 | 0 |
| 1396 | 8.625 | 0 |
| 1397 | 8.625 | 0 |
| 1398 | 8.625 | 0 |
| 1399 | 8.625 | 0 |
| 1400 | 8.625 | 0 |
| 1401 | 8.625 | 0 |
| 1402 | 8.625 | 0 |
| 1403 | 8.625 | 0 |
| 1404 | 8.625 | 0 |
| 1405 | 8.625 | 0 |
| 1406 | 8.625 | 0 |
| 1407 | 8.625 | 0 |
| 1408 | 8.625 | 0 |
| 1409 | 8.625 | 0 |
| 1410 | 8.625 | 0 |
| 1411 | 8.625 | 0 |
| 1412 | 8.625 | 0 |
| 1413 | 8.625 | 0 |
| 1414 | 8.625 | 0 |
| 1415 | 8.875 | 0 |
| 1416 | 8.875 | 0 |
| 1417 | 8.875 | 0 |
| 1418 | 8.875 | 0 |
| 1419 | 8.875 | 0 |
| 1420 | 8.875 | 0 |
| 1421 | 8.875 | 0 |
| 1422 | 8.875 | 0 |
| 1423 | 8.875 | 0 |
| 1424 | 8.875 | 0 |
| 1425 | 8.875 | 0 |
| 1426 | 8.875 | 0 |
| 1427 | 8.875 | 0 |
| 1428 | 8.875 | 0 |
| 1429 | 8.875 | 0 |
| 1430 | 8.875 | 0 |
| 1431 | 8.875 | 0 |
| 1432 | 8.875 | 0 |
| 1433 | 8.875 | 0 |
| 1434 | 8.875 | 0 |
| 1435 | 8.875 | 0 |
| 1436 | 8.875 | 0 |
| 1437 | 8.875 | 0 |
| 1438 | 8.875 | 0 |
| 1439 | 8.875 | 0 |
| 1440 | 8.875 | 0 |
| 1441 | 8.875 | 0 |
| 1442 | 8.875 | 0 |
| 1443 | 8.875 | 0 |
| 1444 | 8.875 | 0 |
| 1445 | 8.875 | 0 |
| 1446 | 8.875 | 0 |
| 1447 | 8.875 | 0 |
| 1448 | 8.875 | 0 |
| 1449 | 8.875 | 0 |
| 1450 | 8.875 | 0 |
| 1451 | 8.875 | 0 |
| 1452 | 8.875 | 0 |
| 1453 | 8.875 | 0 |
| 1454 | 8.875 | 0 |
| 1455 | 8.875 | 0 |
| 1456 | 8.875 | 0 |
| 1457 | 8.875 | 0 |
| 1458 | 8.875 | 0 |
| 1459 | 8.875 | 0 |
| 1460 | 8.875 | 0 |
| 1461 | 8.875 | 0 |
| 1462 | 8.875 | 0 |
| 1463 | 8.875 | 0 |
| 1464 | 8.875 | 0 |
| 1465 | 8.875 | 0 |
| 1466 | 8.875 | 0 |
| 1467 | 8.875 | 0 |
| 1468 | 8.875 | 0 |
| 1469 | 8.875 | 0 |
| 1470 | 8.875 | 0 |
| 1471 | 8.875 | 0 |
| 1472 | 8.875 | 0 |
| 1473 | 8.875 | 0 |
| 1474 | 8.875 | 0 |
| 1475 | 8.875 | 0 |
| 1476 | 8.875 | 0 |
| 1477 | 8.875 | 0 |
| 1478 | 8.875 | 0 |

| | | |
|---|---|---|
| 1391 | 8.375 | 0 |
| 1392 | 8.625 | 0 |
| 1393 | 8.625 | 0 |
| 1394 | 8.625 | 0 |
| 1395 | 8.625 | 0 |
| 1396 | 8.625 | 0 |
| 1397 | 8.625 | 0 |
| 1398 | 8.625 | 0 |
| 1399 | 8.625 | 0 |
| 1400 | 8.625 | 0 |
| 1401 | 8.625 | 0 |
| 1402 | 8.625 | 0 |
| 1403 | 8.625 | 0 |
| 1404 | 8.625 | 0 |
| 1405 | 8.625 | 0 |
| 1406 | 8.625 | 0 |
| 1407 | 8.625 | 0 |
| 1408 | 8.625 | 0 |
| 1409 | 8.625 | 0 |
| 1410 | 8.625 | 0 |
| 1411 | 8.625 | 0 |
| 1412 | 8.625 | 0 |
| 1413 | 8.625 | 0 |
| 1414 | 8.625 | 0 |
| 1415 | 8.625 | 0 |
| 1416 | 8.625 | 0 |
| 1417 | 8.625 | 0 |
| 1418 | 8.625 | 0 |
| 1419 | 8.875 | 0 |
| 1420 | 8.875 | 0 |
| 1421 | 8.875 | 0 |
| 1422 | 8.875 | 0 |
| 1423 | 8.875 | 0 |
| 1424 | 8.875 | 0 |
| 1425 | 8.875 | 0 |
| 1426 | 8.875 | 0 |
| 1427 | 8.875 | 0 |
| 1428 | 8.875 | 0 |
| 1429 | 8.875 | 0 |
| 1430 | 8.875 | 0 |
| 1431 | 8.875 | 0 |
| 1432 | 8.875 | 0 |
| 1433 | 8.875 | 0 |
| 1434 | 8.875 | 0 |
| 1435 | 8.875 | 0 |
| 1436 | 8.875 | 0 |
| 1437 | 8.875 | 0 |
| 1438 | 8.875 | 0 |
| 1439 | 8.875 | 0 |
| 1440 | 8.875 | 0 |
| 1441 | 8.875 | 0 |
| 1442 | 8.875 | 0 |
| 1443 | 8.875 | 0 |
| 1444 | 8.875 | 0 |
| 1445 | 8.875 | 0 |
| 1446 | 8.875 | 0 |
| 1447 | 8.875 | 0 |
| 1448 | 8.875 | 0 |
| 1449 | 8.875 | 0 |
| 1450 | 8.875 | 0 |
| 1451 | 8.875 | 0 |
| 1452 | 8.875 | 0 |
| 1453 | 8.875 | 0 |
| 1454 | 8.875 | 0 |
| 1455 | 8.875 | 0 |
| 1456 | 8.875 | 0 |
| 1457 | 8.875 | 0 |
| 1458 | 8.875 | 0 |
| 1459 | 8.875 | 0 |
| 1460 | 8.875 | 0 |
| 1461 | 8.875 | 0 |
| 1462 | 8.875 | 0 |
| 1463 | 8.875 | 0 |
| 1464 | 8.875 | 0 |
| 1465 | 8.875 | 0 |
| 1466 | 8.875 | 0 |
| 1467 | 8.875 | 0 |
| 1468 | 8.875 | 0 |
| 1469 | 8.875 | 0 |
| 1470 | 8.875 | 0 |
| 1471 | 8.875 | 0 |
| 1472 | 8.875 | 0 |
| 1473 | 8.875 | 0 |
| 1474 | 8.875 | 0 |
| 1475 | 8.875 | 0 |
| 1476 | 8.875 | 0 |
| 1477 | 8.875 | 0 |
| 1478 | 8.875 | 0 |
| 1479 | 8.875 | 0 |
| 1480 | 8.875 | 0 |
| 1481 | 8.875 | 0 |

Generated patient level data* based on Figure 1 from Schippinger et al. (2007)

* Based on the method described by Hoyle and Henley (2011)

| Control | | |
|---|---|---|
| ID | Follow_up | Event |
| 1 | 0.209 | 1 |
| 2 | 0.209 | 1 |
| 3 | 0.209 | 1 |
| 4 | 0.209 | 1 |
| 5 | 0.209 | 1 |
| 6 | 0.209 | 1 |
| 7 | 0.209 | 1 |
| 8 | 0.209 | 1 |
| 9 | 0.209 | 1 |
| 10 | 0.209 | 1 |
| 11 | 0.209 | 1 |
| 12 | 0.209 | 1 |
| 13 | 0.209 | 1 |
| 14 | 0.626 | 1 |
| 15 | 0.626 | 1 |
| 16 | 0.626 | 1 |
| 17 | 0.626 | 1 |
| 18 | 0.626 | 1 |
| 19 | 0.626 | 1 |
| 20 | 0.626 | 1 |
| 21 | 1.043 | 1 |
| 22 | 1.043 | 1 |
| 23 | 1.043 | 1 |
| 24 | 1.043 | 1 |
| 25 | 1.043 | 1 |
| 26 | 1.043 | 1 |
| 27 | 1.043 | 1 |
| 28 | 1.043 | 1 |
| 29 | 1.043 | 1 |
| 30 | 1.043 | 1 |
| 31 | 1.460 | 1 |
| 32 | 1.877 | 1 |
| 33 | 1.877 | 1 |
| 34 | 1.877 | 1 |
| 35 | 1.877 | 1 |
| 36 | 2.294 | 1 |
| 37 | 2.294 | 1 |
| 38 | 2.294 | 1 |
| 39 | 2.294 | 1 |
| 40 | 2.711 | 1 |
| 41 | 3.128 | 1 |
| 42 | 3.128 | 1 |
| 43 | 3.128 | 1 |
| 44 | 3.962 | 1 |
| 45 | 4.379 | 1 |
| 46 | 4.379 | 1 |
| 47 | 4.379 | 1 |
| 48 | 7.715 | 1 |
| 49 | 8.132 | 1 |
| 50 | 0.209 | 0 |
| 51 | 0.209 | 0 |
| 52 | 0.626 | 0 |
| 53 | 0.626 | 0 |
| 54 | 1.043 | 0 |
| 55 | 1.043 | 0 |
| 56 | 1.460 | 0 |
| 57 | 1.460 | 0 |
| 58 | 1.877 | 0 |
| 59 | 1.877 | 0 |
| 60 | 1.877 | 0 |
| 61 | 1.877 | 0 |
| 62 | 1.877 | 0 |
| 63 | 2.294 | 0 |
| 64 | 2.294 | 0 |
| 65 | 2.294 | 0 |
| 66 | 2.294 | 0 |
| 67 | 2.294 | 0 |
| 68 | 2.711 | 0 |
| 69 | 2.711 | 0 |
| 70 | 2.711 | 0 |
| 71 | 2.711 | 0 |
| 72 | 2.711 | 0 |
| 73 | 3.128 | 0 |
| 74 | 3.128 | 0 |
| 75 | 3.128 | 0 |
| 76 | 3.128 | 0 |
| 77 | 3.128 | 0 |
| 78 | 3.545 | 0 |
| 79 | 3.545 | 0 |
| 80 | 3.545 | 0 |
| 81 | 3.545 | 0 |
| 82 | 3.545 | 0 |
| 83 | 3.545 | 0 |
| 84 | 3.545 | 0 |
| 85 | 3.545 | 0 |
| 86 | 3.962 | 0 |
| 87 | 3.962 | 0 |
| 88 | 3.962 | 0 |

| Fluoropyrimidine | | |
|---|---|---|
| ID | Follow_up | Event |
| 1 | 0.209 | 1 |
| 2 | 0.209 | 1 |
| 3 | 0.626 | 1 |
| 4 | 0.626 | 1 |
| 5 | 0.626 | 1 |
| 6 | 0.626 | 1 |
| 7 | 0.626 | 1 |
| 8 | 1.043 | 1 |
| 9 | 1.043 | 1 |
| 10 | 1.460 | 1 |
| 11 | 1.460 | 1 |
| 12 | 1.460 | 1 |
| 13 | 1.460 | 1 |
| 14 | 1.460 | 1 |
| 15 | 1.460 | 1 |
| 16 | 1.460 | 1 |
| 17 | 1.877 | 1 |
| 18 | 1.877 | 1 |
| 19 | 1.877 | 1 |
| 20 | 1.877 | 1 |
| 21 | 1.877 | 1 |
| 22 | 1.877 | 1 |
| 23 | 2.294 | 1 |
| 24 | 2.711 | 1 |
| 25 | 3.128 | 1 |
| 26 | 3.128 | 1 |
| 27 | 3.128 | 1 |
| 28 | 3.545 | 1 |
| 29 | 3.545 | 1 |
| 30 | 3.545 | 1 |
| 31 | 3.545 | 1 |
| 32 | 3.545 | 1 |
| 33 | 4.379 | 1 |
| 34 | 4.379 | 1 |
| 35 | 4.379 | 1 |
| 36 | 5.213 | 1 |
| 37 | 0.209 | 0 |
| 38 | 0.209 | 0 |
| 39 | 0.626 | 0 |
| 40 | 0.626 | 0 |
| 41 | 1.043 | 0 |
| 42 | 1.043 | 0 |
| 43 | 1.460 | 0 |
| 44 | 1.460 | 0 |
| 45 | 1.877 | 0 |
| 46 | 1.877 | 0 |
| 47 | 1.877 | 0 |
| 48 | 1.877 | 0 |
| 49 | 1.877 | 0 |
| 50 | 1.877 | 0 |
| 51 | 2.294 | 0 |
| 52 | 2.294 | 0 |
| 53 | 2.294 | 0 |
| 54 | 2.294 | 0 |
| 55 | 2.294 | 0 |
| 56 | 2.294 | 0 |
| 57 | 2.711 | 0 |
| 58 | 2.711 | 0 |
| 59 | 2.711 | 0 |
| 60 | 2.711 | 0 |
| 61 | 2.711 | 0 |
| 62 | 2.711 | 0 |
| 63 | 3.128 | 0 |
| 64 | 3.128 | 0 |
| 65 | 3.128 | 0 |
| 66 | 3.128 | 0 |
| 67 | 3.128 | 0 |
| 68 | 3.128 | 0 |
| 69 | 3.545 | 0 |
| 70 | 3.545 | 0 |
| 71 | 3.545 | 0 |
| 72 | 3.545 | 0 |
| 73 | 3.545 | 0 |
| 74 | 3.545 | 0 |
| 75 | 3.545 | 0 |
| 76 | 3.962 | 0 |
| 77 | 3.962 | 0 |
| 78 | 3.962 | 0 |
| 79 | 3.962 | 0 |
| 80 | 3.962 | 0 |
| 81 | 3.962 | 0 |
| 82 | 3.962 | 0 |
| 83 | 4.379 | 0 |
| 84 | 4.379 | 0 |
| 85 | 4.379 | 0 |
| 86 | 4.379 | 0 |
| 87 | 4.379 | 0 |
| 88 | 4.379 | 0 |

| | |
|---|---|
| ID | Patient ID |
| Follow_up | Follow up time in years |
| Event | 0 = no recurrence |
| | 1 = recurrence |

| | | |
|---|---|---|
| 89 | 3.962 | 0 |
| 90 | 3.962 | 0 |
| 91 | 3.962 | 0 |
| 92 | 3.962 | 0 |
| 93 | 3.962 | 0 |
| 94 | 4.379 | 0 |
| 95 | 4.379 | 0 |
| 96 | 4.379 | 0 |
| 97 | 4.379 | 0 |
| 98 | 4.379 | 0 |
| 99 | 4.379 | 0 |
| 100 | 4.379 | 0 |
| 101 | 4.379 | 0 |
| 102 | 4.796 | 0 |
| 103 | 4.796 | 0 |
| 104 | 4.796 | 0 |
| 105 | 4.796 | 0 |
| 106 | 4.796 | 0 |
| 107 | 4.796 | 0 |
| 108 | 4.796 | 0 |
| 109 | 4.796 | 0 |
| 110 | 5.213 | 0 |
| 111 | 5.213 | 0 |
| 112 | 5.213 | 0 |
| 113 | 5.213 | 0 |
| 114 | 5.213 | 0 |
| 115 | 5.213 | 0 |
| 116 | 5.213 | 0 |
| 117 | 5.213 | 0 |
| 118 | 5.213 | 0 |
| 119 | 5.213 | 0 |
| 120 | 5.213 | 0 |
| 121 | 5.630 | 0 |
| 122 | 5.630 | 0 |
| 123 | 5.630 | 0 |
| 124 | 5.630 | 0 |
| 125 | 5.630 | 0 |
| 126 | 5.630 | 0 |
| 127 | 5.630 | 0 |
| 128 | 5.630 | 0 |
| 129 | 5.630 | 0 |
| 130 | 5.630 | 0 |
| 131 | 5.630 | 0 |
| 132 | 6.047 | 0 |
| 133 | 6.047 | 0 |
| 134 | 6.047 | 0 |
| 135 | 6.047 | 0 |
| 136 | 6.047 | 0 |
| 137 | 6.047 | 0 |
| 138 | 6.047 | 0 |
| 139 | 6.047 | 0 |
| 140 | 6.047 | 0 |
| 141 | 6.047 | 0 |
| 142 | 6.047 | 0 |
| 143 | 6.464 | 0 |
| 144 | 6.464 | 0 |
| 145 | 6.464 | 0 |
| 146 | 6.464 | 0 |
| 147 | 6.464 | 0 |
| 148 | 6.464 | 0 |
| 149 | 6.464 | 0 |
| 150 | 6.464 | 0 |
| 151 | 6.464 | 0 |
| 152 | 6.464 | 0 |
| 153 | 6.464 | 0 |
| 154 | 6.881 | 0 |
| 155 | 6.881 | 0 |
| 156 | 6.881 | 0 |
| 157 | 6.881 | 0 |
| 158 | 6.881 | 0 |
| 159 | 6.881 | 0 |
| 160 | 6.881 | 0 |
| 161 | 6.881 | 0 |
| 162 | 6.881 | 0 |
| 163 | 6.881 | 0 |
| 164 | 6.881 | 0 |
| 165 | 7.298 | 0 |
| 166 | 7.298 | 0 |
| 167 | 7.298 | 0 |
| 168 | 7.298 | 0 |
| 169 | 7.298 | 0 |
| 170 | 7.298 | 0 |
| 171 | 7.298 | 0 |
| 172 | 7.298 | 0 |
| 173 | 7.298 | 0 |
| 174 | 7.298 | 0 |
| 175 | 7.298 | 0 |
| 176 | 7.715 | 0 |
| 177 | 7.715 | 0 |
| 178 | 7.715 | 0 |
| 179 | 7.715 | 0 |
| 180 | 7.715 | 0 |
| 181 | 7.715 | 0 |

| | | |
|---|---|---|
| 89 | 4.379 | 0 |
| 90 | 4.796 | 0 |
| 91 | 4.796 | 0 |
| 92 | 4.796 | 0 |
| 93 | 4.796 | 0 |
| 94 | 4.796 | 0 |
| 95 | 4.796 | 0 |
| 96 | 4.796 | 0 |
| 97 | 5.213 | 0 |
| 98 | 5.213 | 0 |
| 99 | 5.213 | 0 |
| 100 | 5.213 | 0 |
| 101 | 5.213 | 0 |
| 102 | 5.213 | 0 |
| 103 | 5.213 | 0 |
| 104 | 5.213 | 0 |
| 105 | 5.213 | 0 |
| 106 | 5.213 | 0 |
| 107 | 5.630 | 0 |
| 108 | 5.630 | 0 |
| 109 | 5.630 | 0 |
| 110 | 5.630 | 0 |
| 111 | 5.630 | 0 |
| 112 | 5.630 | 0 |
| 113 | 5.630 | 0 |
| 114 | 5.630 | 0 |
| 115 | 5.630 | 0 |
| 116 | 5.630 | 0 |
| 117 | 6.047 | 0 |
| 118 | 6.047 | 0 |
| 119 | 6.047 | 0 |
| 120 | 6.047 | 0 |
| 121 | 6.047 | 0 |
| 122 | 6.047 | 0 |
| 123 | 6.047 | 0 |
| 124 | 6.047 | 0 |
| 125 | 6.047 | 0 |
| 126 | 6.047 | 0 |
| 127 | 6.464 | 0 |
| 128 | 6.464 | 0 |
| 129 | 6.464 | 0 |
| 130 | 6.464 | 0 |
| 131 | 6.464 | 0 |
| 132 | 6.464 | 0 |
| 133 | 6.464 | 0 |
| 134 | 6.464 | 0 |
| 135 | 6.464 | 0 |
| 136 | 6.464 | 0 |
| 137 | 6.881 | 0 |
| 138 | 6.881 | 0 |
| 139 | 6.881 | 0 |
| 140 | 6.881 | 0 |
| 141 | 6.881 | 0 |
| 142 | 6.881 | 0 |
| 143 | 6.881 | 0 |
| 144 | 6.881 | 0 |
| 145 | 6.881 | 0 |
| 146 | 6.881 | 0 |
| 147 | 6.881 | 0 |
| 148 | 6.881 | 0 |
| 149 | 6.881 | 0 |
| 150 | 6.881 | 0 |
| 151 | 6.881 | 0 |
| 152 | 7.298 | 0 |
| 153 | 7.298 | 0 |
| 154 | 7.298 | 0 |
| 155 | 7.298 | 0 |
| 156 | 7.298 | 0 |
| 157 | 7.298 | 0 |
| 158 | 7.298 | 0 |
| 159 | 7.298 | 0 |
| 160 | 7.298 | 0 |
| 161 | 7.298 | 0 |
| 162 | 7.298 | 0 |
| 163 | 7.298 | 0 |
| 164 | 7.298 | 0 |
| 165 | 7.298 | 0 |
| 166 | 7.298 | 0 |
| 167 | 7.715 | 0 |
| 168 | 7.715 | 0 |
| 169 | 7.715 | 0 |
| 170 | 7.715 | 0 |
| 171 | 7.715 | 0 |
| 172 | 7.715 | 0 |
| 173 | 7.715 | 0 |
| 174 | 7.715 | 0 |
| 175 | 7.715 | 0 |
| 176 | 7.715 | 0 |
| 177 | 7.715 | 0 |
| 178 | 7.715 | 0 |
| 179 | 7.715 | 0 |
| 180 | 7.715 | 0 |
| 181 | 7.715 | 0 |

| | | |
|---|---|---|
| 182 | 7.715 | 0 |
| 183 | 7.715 | 0 |
| 184 | 7.715 | 0 |
| 185 | 7.715 | 0 |
| 186 | 7.715 | 0 |
| 187 | 8.132 | 0 |
| 188 | 8.132 | 0 |
| 189 | 8.132 | 0 |
| 190 | 8.132 | 0 |
| 191 | 8.132 | 0 |
| 192 | 8.132 | 0 |
| 193 | 8.132 | 0 |
| 194 | 8.132 | 0 |
| 195 | 8.132 | 0 |
| 196 | 8.132 | 0 |
| 197 | 8.132 | 0 |
| 198 | 8.549 | 0 |
| 199 | 8.549 | 0 |
| 200 | 8.549 | 0 |
| 201 | 8.549 | 0 |
| 202 | 8.549 | 0 |
| 203 | 8.549 | 0 |
| 204 | 8.549 | 0 |
| 205 | 8.549 | 0 |
| 206 | 8.966 | 0 |
| 207 | 8.966 | 0 |
| 208 | 8.966 | 0 |
| 209 | 8.966 | 0 |
| 210 | 8.966 | 0 |
| 211 | 8.966 | 0 |
| 212 | 8.966 | 0 |
| 213 | 8.966 | 0 |
| 214 | 9.383 | 0 |
| 215 | 9.383 | 0 |
| 216 | 9.383 | 0 |
| 217 | 9.383 | 0 |
| 218 | 9.383 | 0 |
| 219 | 9.383 | 0 |
| 220 | 9.383 | 0 |
| 221 | 9.383 | 0 |
| 222 | 9.800 | 0 |
| 223 | 9.800 | 0 |
| 224 | 9.800 | 0 |
| 225 | 9.800 | 0 |
| 226 | 9.800 | 0 |
| 227 | 9.800 | 0 |
| 228 | 9.800 | 0 |
| 229 | 9.800 | 0 |
| 230 | 10.217 | 0 |
| 231 | 10.217 | 0 |
| 232 | 10.217 | 0 |
| 233 | 10.217 | 0 |
| 234 | 10.217 | 0 |
| 235 | 10.634 | 0 |
| 236 | 10.634 | 0 |
| 237 | 10.634 | 0 |
| 238 | 10.634 | 0 |
| 239 | 10.634 | 0 |
| 240 | 11.051 | 0 |
| 241 | 11.051 | 0 |
| 242 | 11.051 | 0 |
| 243 | 11.051 | 0 |
| 244 | 11.051 | 0 |
| 245 | 11.468 | 0 |
| 246 | 11.468 | 0 |
| 247 | 11.468 | 0 |
| 248 | 11.468 | 0 |
| 249 | 11.468 | 0 |
| 250 | 11.468 | 0 |
| 251 | 11.468 | 0 |
| 252 | 11.468 | 0 |
| 253 | 11.468 | 0 |

| | | |
|---|---|---|
| 182 | 8.132 | 0 |
| 183 | 8.132 | 0 |
| 184 | 8.132 | 0 |
| 185 | 8.132 | 0 |
| 186 | 8.132 | 0 |
| 187 | 8.132 | 0 |
| 188 | 8.132 | 0 |
| 189 | 8.132 | 0 |
| 190 | 8.132 | 0 |
| 191 | 8.132 | 0 |
| 192 | 8.132 | 0 |
| 193 | 8.132 | 0 |
| 194 | 8.132 | 0 |
| 195 | 8.132 | 0 |
| 196 | 8.132 | 0 |
| 197 | 8.549 | 0 |
| 198 | 8.549 | 0 |
| 199 | 8.549 | 0 |
| 200 | 8.549 | 0 |
| 201 | 8.549 | 0 |
| 202 | 8.549 | 0 |
| 203 | 8.549 | 0 |
| 204 | 8.549 | 0 |
| 205 | 8.966 | 0 |
| 206 | 8.966 | 0 |
| 207 | 8.966 | 0 |
| 208 | 8.966 | 0 |
| 209 | 8.966 | 0 |
| 210 | 8.966 | 0 |
| 211 | 8.966 | 0 |
| 212 | 8.966 | 0 |
| 213 | 9.383 | 0 |
| 214 | 9.383 | 0 |
| 215 | 9.383 | 0 |
| 216 | 9.383 | 0 |
| 217 | 9.383 | 0 |
| 218 | 9.383 | 0 |
| 219 | 9.383 | 0 |
| 220 | 9.383 | 0 |
| 221 | 9.800 | 0 |
| 222 | 9.800 | 0 |
| 223 | 9.800 | 0 |
| 224 | 9.800 | 0 |
| 225 | 9.800 | 0 |
| 226 | 9.800 | 0 |
| 227 | 9.800 | 0 |
| 228 | 9.800 | 0 |
| 229 | 10.217 | 0 |
| 230 | 10.217 | 0 |
| 231 | 10.217 | 0 |
| 232 | 10.217 | 0 |
| 233 | 10.217 | 0 |
| 234 | 10.217 | 0 |
| 235 | 10.217 | 0 |
| 236 | 10.634 | 0 |
| 237 | 10.634 | 0 |
| 238 | 10.634 | 0 |
| 239 | 10.634 | 0 |
| 240 | 10.634 | 0 |
| 241 | 10.634 | 0 |
| 242 | 10.634 | 0 |
| 243 | 11.051 | 0 |
| 244 | 11.051 | 0 |
| 245 | 11.051 | 0 |
| 246 | 11.051 | 0 |
| 247 | 11.051 | 0 |
| 248 | 11.051 | 0 |
| 249 | 11.051 | 0 |
| 250 | 11.468 | 0 |
| 251 | 11.468 | 0 |
| 252 | 11.468 | 0 |
| 253 | 11.468 | 0 |
| 254 | 11.468 | 0 |
| 255 | 11.468 | 0 |
| 256 | 11.468 | 0 |
| 257 | 11.468 | 0 |

Generated patient level data* based on Figure 4b from Andre et al. (2009)

* Based on the method described by Hoyle and Henley (2011)

| Fluoropyrimidine monotherapy | | |
|---|---|---|
| ID | Follow_up | Event |
| 1 | 0.063 | 1 |
| 2 | 0.188 | 1 |
| 3 | 0.188 | 1 |
| 4 | 0.313 | 1 |
| 5 | 0.313 | 1 |
| 6 | 0.313 | 1 |
| 7 | 0.438 | 1 |
| 8 | 0.438 | 1 |
| 9 | 0.438 | 1 |
| 10 | 0.563 | 1 |
| 11 | 0.563 | 1 |
| 12 | 0.563 | 1 |
| 13 | 0.563 | 1 |
| 14 | 0.563 | 1 |
| 15 | 0.563 | 1 |
| 16 | 0.688 | 1 |
| 17 | 0.688 | 1 |
| 18 | 0.688 | 1 |
| 19 | 0.688 | 1 |
| 20 | 0.813 | 1 |
| 21 | 0.813 | 1 |
| 22 | 0.813 | 1 |
| 23 | 0.813 | 1 |
| 24 | 0.938 | 1 |
| 25 | 0.938 | 1 |
| 26 | 1.063 | 1 |
| 27 | 1.063 | 1 |
| 28 | 1.063 | 1 |
| 29 | 1.063 | 1 |
| 30 | 1.063 | 1 |
| 31 | 1.063 | 1 |
| 32 | 1.063 | 1 |
| 33 | 1.063 | 1 |
| 34 | 1.188 | 1 |
| 35 | 1.188 | 1 |
| 36 | 1.188 | 1 |
| 37 | 1.188 | 1 |
| 38 | 1.313 | 1 |
| 39 | 1.313 | 1 |
| 40 | 1.438 | 1 |
| 41 | 1.438 | 1 |
| 42 | 1.438 | 1 |
| 43 | 1.563 | 1 |
| 44 | 1.563 | 1 |
| 45 | 1.563 | 1 |
| 46 | 1.563 | 1 |
| 47 | 1.563 | 1 |
| 48 | 1.563 | 1 |
| 49 | 1.688 | 1 |
| 50 | 1.813 | 1 |
| 51 | 1.938 | 1 |
| 52 | 1.938 | 1 |
| 53 | 1.938 | 1 |
| 54 | 2.063 | 1 |
| 55 | 2.063 | 1 |
| 56 | 2.063 | 1 |
| 57 | 2.063 | 1 |
| 58 | 2.063 | 1 |
| 59 | 2.188 | 1 |
| 60 | 2.313 | 1 |
| 61 | 2.313 | 1 |
| 62 | 2.313 | 1 |
| 63 | 2.438 | 1 |
| 64 | 2.563 | 1 |
| 65 | 2.563 | 1 |
| 66 | 2.813 | 1 |
| 67 | 2.938 | 1 |
| 68 | 3.063 | 1 |
| 69 | 3.063 | 1 |
| 70 | 3.063 | 1 |
| 71 | 3.063 | 1 |
| 72 | 3.188 | 1 |
| 73 | 3.438 | 1 |
| 74 | 3.438 | 1 |
| 75 | 3.688 | 1 |
| 76 | 3.688 | 1 |
| 77 | 3.813 | 1 |
| 78 | 3.938 | 1 |
| 79 | 4.063 | 1 |
| 80 | 4.188 | 1 |
| 81 | 4.313 | 1 |
| 82 | 4.438 | 1 |
| 83 | 4.438 | 1 |
| 84 | 4.563 | 1 |
| 85 | 4.688 | 1 |
| 86 | 4.688 | 1 |
| 87 | 4.813 | 1 |
| 88 | 0.063 | 0 |

| Fluoropyrimidine combined with oxaliplatin | | |
|---|---|---|
| ID | Follow_up | Event |
| 1 | 0.063 | 1 |
| 2 | 0.188 | 1 |
| 3 | 0.188 | 1 |
| 4 | 0.188 | 1 |
| 5 | 0.313 | 1 |
| 6 | 0.438 | 1 |
| 7 | 0.563 | 1 |
| 8 | 0.563 | 1 |
| 9 | 0.688 | 1 |
| 10 | 0.813 | 1 |
| 11 | 0.813 | 1 |
| 12 | 0.813 | 1 |
| 13 | 0.938 | 1 |
| 14 | 1.063 | 1 |
| 15 | 1.063 | 1 |
| 16 | 1.188 | 1 |
| 17 | 1.188 | 1 |
| 18 | 1.188 | 1 |
| 19 | 1.188 | 1 |
| 20 | 1.313 | 1 |
| 21 | 1.313 | 1 |
| 22 | 1.313 | 1 |
| 23 | 1.313 | 1 |
| 24 | 1.313 | 1 |
| 25 | 1.313 | 1 |
| 26 | 1.438 | 1 |
| 27 | 1.438 | 1 |
| 28 | 1.438 | 1 |
| 29 | 1.438 | 1 |
| 30 | 1.563 | 1 |
| 31 | 1.688 | 1 |
| 32 | 1.688 | 1 |
| 33 | 1.688 | 1 |
| 34 | 1.688 | 1 |
| 35 | 1.813 | 1 |
| 36 | 1.813 | 1 |
| 37 | 1.813 | 1 |
| 38 | 1.813 | 1 |
| 39 | 1.938 | 1 |
| 40 | 1.938 | 1 |
| 41 | 1.938 | 1 |
| 42 | 1.938 | 1 |
| 43 | 2.063 | 1 |
| 44 | 2.063 | 1 |
| 45 | 2.063 | 1 |
| 46 | 2.188 | 1 |
| 47 | 2.188 | 1 |
| 48 | 2.188 | 1 |
| 49 | 2.313 | 1 |
| 50 | 2.313 | 1 |
| 51 | 2.313 | 1 |
| 52 | 2.313 | 1 |
| 53 | 2.313 | 1 |
| 54 | 2.438 | 1 |
| 55 | 2.688 | 1 |
| 56 | 2.688 | 1 |
| 57 | 2.938 | 1 |
| 58 | 2.938 | 1 |
| 59 | 3.438 | 1 |
| 60 | 3.438 | 1 |
| 61 | 3.438 | 1 |
| 62 | 3.438 | 1 |
| 63 | 3.813 | 1 |
| 64 | 3.813 | 1 |
| 65 | 3.938 | 1 |
| 66 | 4.063 | 1 |
| 67 | 4.063 | 1 |
| 68 | 4.188 | 1 |
| 69 | 4.188 | 1 |
| 70 | 4.188 | 1 |
| 71 | 4.188 | 1 |
| 72 | 4.563 | 1 |
| 73 | 4.563 | 1 |
| 74 | 4.688 | 1 |
| 75 | 4.938 | 1 |
| 76 | 5.063 | 1 |
| 77 | 5.188 | 1 |
| 78 | 5.188 | 1 |
| 79 | 5.188 | 1 |
| 80 | 5.313 | 1 |
| 81 | 5.438 | 1 |
| 82 | 5.438 | 1 |
| 83 | 0.563 | 0 |
| 84 | 0.688 | 0 |
| 85 | 0.813 | 0 |
| 86 | 0.938 | 0 |
| 87 | 2.563 | 0 |
| 88 | 2.688 | 0 |

| | |
|---|---|
| ID | Patient ID |
| Follow_up | Follow up time in years |
| Event | 0 = no recurrence |
| | 1 = recurrence |

| | | |
|---|---|---|
| 89 | 0.188 | 0 |
| 90 | 0.313 | 0 |
| 91 | 0.438 | 0 |
| 92 | 1.813 | 0 |
| 93 | 2.563 | 0 |
| 94 | 2.688 | 0 |
| 95 | 2.813 | 0 |
| 96 | 2.938 | 0 |
| 97 | 4.063 | 0 |
| 98 | 4.188 | 0 |
| 99 | 4.313 | 0 |
| 100 | 4.438 | 0 |
| 101 | 4.563 | 0 |
| 102 | 4.563 | 0 |
| 103 | 4.563 | 0 |
| 104 | 4.563 | 0 |
| 105 | 4.563 | 0 |
| 106 | 4.563 | 0 |
| 107 | 4.563 | 0 |
| 108 | 4.563 | 0 |
| 109 | 4.563 | 0 |
| 110 | 4.563 | 0 |
| 111 | 4.563 | 0 |
| 112 | 4.563 | 0 |
| 113 | 4.563 | 0 |
| 114 | 4.688 | 0 |
| 115 | 4.688 | 0 |
| 116 | 4.688 | 0 |
| 117 | 4.688 | 0 |
| 118 | 4.688 | 0 |
| 119 | 4.688 | 0 |
| 120 | 4.688 | 0 |
| 121 | 4.688 | 0 |
| 122 | 4.688 | 0 |
| 123 | 4.688 | 0 |
| 124 | 4.688 | 0 |
| 125 | 4.688 | 0 |
| 126 | 4.688 | 0 |
| 127 | 4.813 | 0 |
| 128 | 4.813 | 0 |
| 129 | 4.813 | 0 |
| 130 | 4.813 | 0 |
| 131 | 4.813 | 0 |
| 132 | 4.813 | 0 |
| 133 | 4.813 | 0 |
| 134 | 4.813 | 0 |
| 135 | 4.813 | 0 |
| 136 | 4.813 | 0 |
| 137 | 4.813 | 0 |
| 138 | 4.813 | 0 |
| 139 | 4.813 | 0 |
| 140 | 4.938 | 0 |
| 141 | 4.938 | 0 |
| 142 | 4.938 | 0 |
| 143 | 4.938 | 0 |
| 144 | 4.938 | 0 |
| 145 | 4.938 | 0 |
| 146 | 4.938 | 0 |
| 147 | 4.938 | 0 |
| 148 | 4.938 | 0 |
| 149 | 4.938 | 0 |
| 150 | 4.938 | 0 |
| 151 | 4.938 | 0 |
| 152 | 4.938 | 0 |
| 153 | 5.063 | 0 |
| 154 | 5.063 | 0 |
| 155 | 5.063 | 0 |
| 156 | 5.063 | 0 |
| 157 | 5.063 | 0 |
| 158 | 5.063 | 0 |
| 159 | 5.063 | 0 |
| 160 | 5.063 | 0 |
| 161 | 5.063 | 0 |
| 162 | 5.063 | 0 |
| 163 | 5.063 | 0 |
| 164 | 5.063 | 0 |
| 165 | 5.063 | 0 |
| 166 | 5.063 | 0 |
| 167 | 5.063 | 0 |
| 168 | 5.063 | 0 |
| 169 | 5.063 | 0 |
| 170 | 5.063 | 0 |
| 171 | 5.063 | 0 |
| 172 | 5.063 | 0 |
| 173 | 5.063 | 0 |
| 174 | 5.063 | 0 |
| 175 | 5.063 | 0 |
| 176 | 5.063 | 0 |
| 177 | 5.063 | 0 |
| 178 | 5.063 | 0 |
| 179 | 5.063 | 0 |
| 180 | 5.063 | 0 |
| 181 | 5.063 | 0 |

| | | |
|---|---|---|
| 89 | 2.813 | 0 |
| 90 | 2.938 | 0 |
| 91 | 3.563 | 0 |
| 92 | 3.688 | 0 |
| 93 | 3.813 | 0 |
| 94 | 3.938 | 0 |
| 95 | 4.063 | 0 |
| 96 | 4.063 | 0 |
| 97 | 4.188 | 0 |
| 98 | 4.188 | 0 |
| 99 | 4.313 | 0 |
| 100 | 4.313 | 0 |
| 101 | 4.438 | 0 |
| 102 | 4.438 | 0 |
| 103 | 4.563 | 0 |
| 104 | 4.563 | 0 |
| 105 | 4.563 | 0 |
| 106 | 4.563 | 0 |
| 107 | 4.563 | 0 |
| 108 | 4.563 | 0 |
| 109 | 4.563 | 0 |
| 110 | 4.563 | 0 |
| 111 | 4.563 | 0 |
| 112 | 4.563 | 0 |
| 113 | 4.563 | 0 |
| 114 | 4.563 | 0 |
| 115 | 4.563 | 0 |
| 116 | 4.563 | 0 |
| 117 | 4.563 | 0 |
| 118 | 4.563 | 0 |
| 119 | 4.563 | 0 |
| 120 | 4.688 | 0 |
| 121 | 4.688 | 0 |
| 122 | 4.688 | 0 |
| 123 | 4.688 | 0 |
| 124 | 4.688 | 0 |
| 125 | 4.688 | 0 |
| 126 | 4.688 | 0 |
| 127 | 4.688 | 0 |
| 128 | 4.688 | 0 |
| 129 | 4.688 | 0 |
| 130 | 4.688 | 0 |
| 131 | 4.688 | 0 |
| 132 | 4.688 | 0 |
| 133 | 4.688 | 0 |
| 134 | 4.688 | 0 |
| 135 | 4.688 | 0 |
| 136 | 4.688 | 0 |
| 137 | 4.813 | 0 |
| 138 | 4.813 | 0 |
| 139 | 4.813 | 0 |
| 140 | 4.813 | 0 |
| 141 | 4.813 | 0 |
| 142 | 4.813 | 0 |
| 143 | 4.813 | 0 |
| 144 | 4.813 | 0 |
| 145 | 4.813 | 0 |
| 146 | 4.813 | 0 |
| 147 | 4.813 | 0 |
| 148 | 4.813 | 0 |
| 149 | 4.813 | 0 |
| 150 | 4.813 | 0 |
| 151 | 4.813 | 0 |
| 152 | 4.813 | 0 |
| 153 | 4.813 | 0 |
| 154 | 4.938 | 0 |
| 155 | 4.938 | 0 |
| 156 | 4.938 | 0 |
| 157 | 4.938 | 0 |
| 158 | 4.938 | 0 |
| 159 | 4.938 | 0 |
| 160 | 4.938 | 0 |
| 161 | 4.938 | 0 |
| 162 | 4.938 | 0 |
| 163 | 4.938 | 0 |
| 164 | 4.938 | 0 |
| 165 | 4.938 | 0 |
| 166 | 4.938 | 0 |
| 167 | 4.938 | 0 |
| 168 | 4.938 | 0 |
| 169 | 4.938 | 0 |
| 170 | 4.938 | 0 |
| 171 | 5.063 | 0 |
| 172 | 5.063 | 0 |
| 173 | 5.063 | 0 |
| 174 | 5.063 | 0 |
| 175 | 5.063 | 0 |
| 176 | 5.063 | 0 |
| 177 | 5.063 | 0 |
| 178 | 5.063 | 0 |
| 179 | 5.063 | 0 |
| 180 | 5.063 | 0 |
| 181 | 5.063 | 0 |

| | | |
|---|---|---|
| 182 | 5.063 | 0 |
| 183 | 5.063 | 0 |
| 184 | 5.063 | 0 |
| 185 | 5.063 | 0 |
| 186 | 5.063 | 0 |
| 187 | 5.063 | 0 |
| 188 | 5.063 | 0 |
| 189 | 5.063 | 0 |
| 190 | 5.063 | 0 |
| 191 | 5.063 | 0 |
| 192 | 5.063 | 0 |
| 193 | 5.063 | 0 |
| 194 | 5.063 | 0 |
| 195 | 5.063 | 0 |
| 196 | 5.063 | 0 |
| 197 | 5.063 | 0 |
| 198 | 5.063 | 0 |
| 199 | 5.063 | 0 |
| 200 | 5.188 | 0 |
| 201 | 5.188 | 0 |
| 202 | 5.188 | 0 |
| 203 | 5.188 | 0 |
| 204 | 5.188 | 0 |
| 205 | 5.188 | 0 |
| 206 | 5.188 | 0 |
| 207 | 5.188 | 0 |
| 208 | 5.188 | 0 |
| 209 | 5.188 | 0 |
| 210 | 5.188 | 0 |
| 211 | 5.188 | 0 |
| 212 | 5.188 | 0 |
| 213 | 5.188 | 0 |
| 214 | 5.188 | 0 |
| 215 | 5.188 | 0 |
| 216 | 5.188 | 0 |
| 217 | 5.188 | 0 |
| 218 | 5.188 | 0 |
| 219 | 5.188 | 0 |
| 220 | 5.188 | 0 |
| 221 | 5.188 | 0 |
| 222 | 5.188 | 0 |
| 223 | 5.188 | 0 |
| 224 | 5.188 | 0 |
| 225 | 5.188 | 0 |
| 226 | 5.188 | 0 |
| 227 | 5.188 | 0 |
| 228 | 5.188 | 0 |
| 229 | 5.188 | 0 |
| 230 | 5.188 | 0 |
| 231 | 5.188 | 0 |
| 232 | 5.188 | 0 |
| 233 | 5.188 | 0 |
| 234 | 5.188 | 0 |
| 235 | 5.188 | 0 |
| 236 | 5.188 | 0 |
| 237 | 5.188 | 0 |
| 238 | 5.188 | 0 |
| 239 | 5.188 | 0 |
| 240 | 5.188 | 0 |
| 241 | 5.188 | 0 |
| 242 | 5.188 | 0 |
| 243 | 5.188 | 0 |
| 244 | 5.188 | 0 |
| 245 | 5.188 | 0 |
| 246 | 5.188 | 0 |
| 247 | 5.313 | 0 |
| 248 | 5.313 | 0 |
| 249 | 5.313 | 0 |
| 250 | 5.313 | 0 |
| 251 | 5.313 | 0 |
| 252 | 5.313 | 0 |
| 253 | 5.313 | 0 |
| 254 | 5.313 | 0 |
| 255 | 5.313 | 0 |
| 256 | 5.313 | 0 |
| 257 | 5.313 | 0 |
| 258 | 5.313 | 0 |
| 259 | 5.313 | 0 |
| 260 | 5.313 | 0 |
| 261 | 5.313 | 0 |
| 262 | 5.313 | 0 |
| 263 | 5.313 | 0 |
| 264 | 5.313 | 0 |
| 265 | 5.313 | 0 |
| 266 | 5.313 | 0 |
| 267 | 5.313 | 0 |
| 268 | 5.313 | 0 |
| 269 | 5.313 | 0 |
| 270 | 5.313 | 0 |
| 271 | 5.313 | 0 |
| 272 | 5.313 | 0 |
| 273 | 5.313 | 0 |
| 274 | 5.313 | 0 |

| | | |
|---|---|---|
| 182 | 5.063 | 0 |
| 183 | 5.063 | 0 |
| 184 | 5.063 | 0 |
| 185 | 5.063 | 0 |
| 186 | 5.063 | 0 |
| 187 | 5.063 | 0 |
| 188 | 5.063 | 0 |
| 189 | 5.063 | 0 |
| 190 | 5.063 | 0 |
| 191 | 5.063 | 0 |
| 192 | 5.063 | 0 |
| 193 | 5.063 | 0 |
| 194 | 5.063 | 0 |
| 195 | 5.063 | 0 |
| 196 | 5.063 | 0 |
| 197 | 5.063 | 0 |
| 198 | 5.063 | 0 |
| 199 | 5.063 | 0 |
| 200 | 5.063 | 0 |
| 201 | 5.063 | 0 |
| 202 | 5.063 | 0 |
| 203 | 5.063 | 0 |
| 204 | 5.063 | 0 |
| 205 | 5.063 | 0 |
| 206 | 5.063 | 0 |
| 207 | 5.063 | 0 |
| 208 | 5.063 | 0 |
| 209 | 5.063 | 0 |
| 210 | 5.063 | 0 |
| 211 | 5.188 | 0 |
| 212 | 5.188 | 0 |
| 213 | 5.188 | 0 |
| 214 | 5.188 | 0 |
| 215 | 5.188 | 0 |
| 216 | 5.188 | 0 |
| 217 | 5.188 | 0 |
| 218 | 5.188 | 0 |
| 219 | 5.188 | 0 |
| 220 | 5.188 | 0 |
| 221 | 5.188 | 0 |
| 222 | 5.188 | 0 |
| 223 | 5.188 | 0 |
| 224 | 5.188 | 0 |
| 225 | 5.188 | 0 |
| 226 | 5.188 | 0 |
| 227 | 5.188 | 0 |
| 228 | 5.188 | 0 |
| 229 | 5.188 | 0 |
| 230 | 5.188 | 0 |
| 231 | 5.188 | 0 |
| 232 | 5.188 | 0 |
| 233 | 5.188 | 0 |
| 234 | 5.188 | 0 |
| 235 | 5.188 | 0 |
| 236 | 5.188 | 0 |
| 237 | 5.188 | 0 |
| 238 | 5.188 | 0 |
| 239 | 5.188 | 0 |
| 240 | 5.188 | 0 |
| 241 | 5.188 | 0 |
| 242 | 5.188 | 0 |
| 243 | 5.188 | 0 |
| 244 | 5.188 | 0 |
| 245 | 5.188 | 0 |
| 246 | 5.188 | 0 |
| 247 | 5.188 | 0 |
| 248 | 5.188 | 0 |
| 249 | 5.188 | 0 |
| 250 | 5.188 | 0 |
| 251 | 5.313 | 0 |
| 252 | 5.313 | 0 |
| 253 | 5.313 | 0 |
| 254 | 5.313 | 0 |
| 255 | 5.313 | 0 |
| 256 | 5.313 | 0 |
| 257 | 5.313 | 0 |
| 258 | 5.313 | 0 |
| 259 | 5.313 | 0 |
| 260 | 5.313 | 0 |
| 261 | 5.313 | 0 |
| 262 | 5.313 | 0 |
| 263 | 5.313 | 0 |
| 264 | 5.313 | 0 |
| 265 | 5.313 | 0 |
| 266 | 5.313 | 0 |
| 267 | 5.313 | 0 |
| 268 | 5.313 | 0 |
| 269 | 5.313 | 0 |
| 270 | 5.313 | 0 |
| 271 | 5.313 | 0 |
| 272 | 5.313 | 0 |
| 273 | 5.313 | 0 |
| 274 | 5.313 | 0 |

| | | |
|---|---|---|
| 275 | 5.313 | 0 |
| 276 | 5.313 | 0 |
| 277 | 5.313 | 0 |
| 278 | 5.313 | 0 |
| 279 | 5.313 | 0 |
| 280 | 5.313 | 0 |
| 281 | 5.313 | 0 |
| 282 | 5.313 | 0 |
| 283 | 5.313 | 0 |
| 284 | 5.313 | 0 |
| 285 | 5.313 | 0 |
| 286 | 5.313 | 0 |
| 287 | 5.313 | 0 |
| 288 | 5.313 | 0 |
| 289 | 5.313 | 0 |
| 290 | 5.313 | 0 |
| 291 | 5.313 | 0 |
| 292 | 5.313 | 0 |
| 293 | 5.313 | 0 |
| 294 | 5.438 | 0 |
| 295 | 5.438 | 0 |
| 296 | 5.438 | 0 |
| 297 | 5.438 | 0 |
| 298 | 5.438 | 0 |
| 299 | 5.438 | 0 |
| 300 | 5.438 | 0 |
| 301 | 5.438 | 0 |
| 302 | 5.438 | 0 |
| 303 | 5.438 | 0 |
| 304 | 5.438 | 0 |
| 305 | 5.438 | 0 |
| 306 | 5.438 | 0 |
| 307 | 5.438 | 0 |
| 308 | 5.438 | 0 |
| 309 | 5.438 | 0 |
| 310 | 5.438 | 0 |
| 311 | 5.438 | 0 |
| 312 | 5.438 | 0 |
| 313 | 5.438 | 0 |
| 314 | 5.438 | 0 |
| 315 | 5.438 | 0 |
| 316 | 5.438 | 0 |
| 317 | 5.438 | 0 |
| 318 | 5.438 | 0 |
| 319 | 5.438 | 0 |
| 320 | 5.438 | 0 |
| 321 | 5.438 | 0 |
| 322 | 5.438 | 0 |
| 323 | 5.438 | 0 |
| 324 | 5.438 | 0 |
| 325 | 5.438 | 0 |
| 326 | 5.438 | 0 |
| 327 | 5.438 | 0 |
| 328 | 5.438 | 0 |
| 329 | 5.438 | 0 |
| 330 | 5.438 | 0 |
| 331 | 5.438 | 0 |
| 332 | 5.438 | 0 |
| 333 | 5.438 | 0 |
| 334 | 5.438 | 0 |
| 335 | 5.438 | 0 |
| 336 | 5.438 | 0 |
| 337 | 5.438 | 0 |
| 338 | 5.438 | 0 |
| 339 | 5.438 | 0 |
| 340 | 5.438 | 0 |
| 341 | 5.438 | 0 |
| 342 | 5.438 | 0 |
| 343 | 5.438 | 0 |
| 344 | 5.438 | 0 |
| 345 | 5.438 | 0 |
| 346 | 5.438 | 0 |
| 347 | 5.438 | 0 |
| 348 | 5.438 | 0 |
| 349 | 5.438 | 0 |
| 350 | 5.438 | 0 |
| 351 | 5.438 | 0 |
| 352 | 5.438 | 0 |
| 353 | 5.438 | 0 |
| 354 | 5.438 | 0 |
| 355 | 5.438 | 0 |
| 356 | 5.438 | 0 |
| 357 | 5.438 | 0 |
| 358 | 5.438 | 0 |
| 359 | 5.438 | 0 |
| 360 | 5.438 | 0 |
| 361 | 5.438 | 0 |
| 362 | 5.438 | 0 |
| 363 | 5.438 | 0 |
| 364 | 5.438 | 0 |
| 365 | 5.438 | 0 |
| 366 | 5.438 | 0 |
| 367 | 5.438 | 0 |

| | | |
|---|---|---|
| 275 | 5.313 | 0 |
| 276 | 5.313 | 0 |
| 277 | 5.313 | 0 |
| 278 | 5.313 | 0 |
| 279 | 5.313 | 0 |
| 280 | 5.313 | 0 |
| 281 | 5.313 | 0 |
| 282 | 5.313 | 0 |
| 283 | 5.313 | 0 |
| 284 | 5.313 | 0 |
| 285 | 5.313 | 0 |
| 286 | 5.313 | 0 |
| 287 | 5.313 | 0 |
| 288 | 5.313 | 0 |
| 289 | 5.313 | 0 |
| 290 | 5.313 | 0 |
| 291 | 5.438 | 0 |
| 292 | 5.438 | 0 |
| 293 | 5.438 | 0 |
| 294 | 5.438 | 0 |
| 295 | 5.438 | 0 |
| 296 | 5.438 | 0 |
| 297 | 5.438 | 0 |
| 298 | 5.438 | 0 |
| 299 | 5.438 | 0 |
| 300 | 5.438 | 0 |
| 301 | 5.438 | 0 |
| 302 | 5.438 | 0 |
| 303 | 5.438 | 0 |
| 304 | 5.438 | 0 |
| 305 | 5.438 | 0 |
| 306 | 5.438 | 0 |
| 307 | 5.438 | 0 |
| 308 | 5.438 | 0 |
| 309 | 5.438 | 0 |
| 310 | 5.438 | 0 |
| 311 | 5.438 | 0 |
| 312 | 5.438 | 0 |
| 313 | 5.438 | 0 |
| 314 | 5.438 | 0 |
| 315 | 5.438 | 0 |
| 316 | 5.438 | 0 |
| 317 | 5.438 | 0 |
| 318 | 5.438 | 0 |
| 319 | 5.438 | 0 |
| 320 | 5.438 | 0 |
| 321 | 5.438 | 0 |
| 322 | 5.438 | 0 |
| 323 | 5.438 | 0 |
| 324 | 5.438 | 0 |
| 325 | 5.438 | 0 |
| 326 | 5.438 | 0 |
| 327 | 5.438 | 0 |
| 328 | 5.438 | 0 |
| 329 | 5.438 | 0 |
| 330 | 5.438 | 0 |
| 331 | 5.438 | 0 |
| 332 | 5.438 | 0 |
| 333 | 5.438 | 0 |
| 334 | 5.438 | 0 |
| 335 | 5.438 | 0 |
| 336 | 5.438 | 0 |
| 337 | 5.438 | 0 |
| 338 | 5.438 | 0 |
| 339 | 5.438 | 0 |
| 340 | 5.438 | 0 |
| 341 | 5.438 | 0 |
| 342 | 5.438 | 0 |
| 343 | 5.438 | 0 |
| 344 | 5.438 | 0 |
| 345 | 5.438 | 0 |
| 346 | 5.438 | 0 |
| 347 | 5.438 | 0 |
| 348 | 5.438 | 0 |
| 349 | 5.438 | 0 |
| 350 | 5.438 | 0 |
| 351 | 5.438 | 0 |
| 352 | 5.438 | 0 |
| 353 | 5.438 | 0 |
| 354 | 5.438 | 0 |
| 355 | 5.438 | 0 |
| 356 | 5.438 | 0 |
| 357 | 5.438 | 0 |
| 358 | 5.438 | 0 |
| 359 | 5.438 | 0 |
| 360 | 5.438 | 0 |
| 361 | 5.438 | 0 |
| 362 | 5.438 | 0 |
| 363 | 5.438 | 0 |
| 364 | 5.438 | 0 |
| 365 | 5.438 | 0 |
| 366 | 5.438 | 0 |
| 367 | 5.438 | 0 |

| | | |
|---|---|---|
| 368 | 5.438 | 0 |
| 369 | 5.438 | 0 |
| 370 | 5.438 | 0 |
| 371 | 5.438 | 0 |
| 372 | 5.438 | 0 |
| 373 | 5.438 | 0 |
| 374 | 5.438 | 0 |
| 375 | 5.438 | 0 |
| 376 | 5.438 | 0 |
| 377 | 5.438 | 0 |
| 378 | 5.438 | 0 |
| 379 | 5.438 | 0 |
| 380 | 5.438 | 0 |
| 381 | 5.438 | 0 |
| 382 | 5.438 | 0 |
| 383 | 5.438 | 0 |
| 384 | 5.438 | 0 |
| 385 | 5.438 | 0 |
| 386 | 5.438 | 0 |
| 387 | 5.438 | 0 |
| 388 | 5.438 | 0 |
| 389 | 5.438 | 0 |
| 390 | 5.438 | 0 |
| 391 | 5.438 | 0 |
| 392 | 5.438 | 0 |
| 393 | 5.438 | 0 |
| 394 | 5.438 | 0 |
| 395 | 5.438 | 0 |
| 396 | 5.438 | 0 |
| 397 | 5.438 | 0 |
| 398 | 5.438 | 0 |
| 399 | 5.438 | 0 |
| 400 | 5.438 | 0 |
| 401 | 5.438 | 0 |
| 402 | 5.438 | 0 |
| 403 | 5.438 | 0 |
| 404 | 5.438 | 0 |
| 405 | 5.438 | 0 |
| 406 | 5.438 | 0 |
| 407 | 5.438 | 0 |
| 408 | 5.438 | 0 |
| 409 | 5.438 | 0 |
| 410 | 5.438 | 0 |
| 411 | 5.438 | 0 |
| 412 | 5.438 | 0 |
| 413 | 5.438 | 0 |
| 414 | 5.438 | 0 |
| 415 | 5.438 | 0 |
| 416 | 5.438 | 0 |
| 417 | 5.438 | 0 |
| 418 | 5.438 | 0 |
| 419 | 5.438 | 0 |
| 420 | 5.438 | 0 |
| 421 | 5.438 | 0 |
| 422 | 5.438 | 0 |
| 423 | 5.438 | 0 |
| 424 | 5.438 | 0 |
| 425 | 5.438 | 0 |
| 426 | 5.438 | 0 |
| 427 | 5.438 | 0 |
| 428 | 5.438 | 0 |
| 429 | 5.438 | 0 |
| 430 | 5.438 | 0 |
| 431 | 5.438 | 0 |
| 432 | 5.438 | 0 |
| 433 | 5.438 | 0 |
| 434 | 5.438 | 0 |
| 435 | 5.438 | 0 |
| 436 | 5.438 | 0 |
| 437 | 5.438 | 0 |
| 438 | 5.438 | 0 |
| 439 | 5.438 | 0 |
| 440 | 5.438 | 0 |
| 441 | 5.438 | 0 |

| | | |
|---|---|---|
| 368 | 5.438 | 0 |
| 369 | 5.438 | 0 |
| 370 | 5.438 | 0 |
| 371 | 5.438 | 0 |
| 372 | 5.438 | 0 |
| 373 | 5.438 | 0 |
| 374 | 5.438 | 0 |
| 375 | 5.438 | 0 |
| 376 | 5.438 | 0 |
| 377 | 5.438 | 0 |
| 378 | 5.438 | 0 |
| 379 | 5.438 | 0 |
| 380 | 5.438 | 0 |
| 381 | 5.438 | 0 |
| 382 | 5.438 | 0 |
| 383 | 5.438 | 0 |
| 384 | 5.438 | 0 |
| 385 | 5.438 | 0 |
| 386 | 5.438 | 0 |
| 387 | 5.438 | 0 |
| 388 | 5.438 | 0 |
| 389 | 5.438 | 0 |
| 390 | 5.438 | 0 |
| 391 | 5.438 | 0 |
| 392 | 5.438 | 0 |
| 393 | 5.438 | 0 |
| 394 | 5.438 | 0 |
| 395 | 5.438 | 0 |
| 396 | 5.438 | 0 |
| 397 | 5.438 | 0 |
| 398 | 5.438 | 0 |
| 399 | 5.438 | 0 |
| 400 | 5.438 | 0 |
| 401 | 5.438 | 0 |
| 402 | 5.438 | 0 |
| 403 | 5.438 | 0 |
| 404 | 5.438 | 0 |
| 405 | 5.438 | 0 |
| 406 | 5.438 | 0 |
| 407 | 5.438 | 0 |
| 408 | 5.438 | 0 |
| 409 | 5.438 | 0 |
| 410 | 5.438 | 0 |
| 411 | 5.438 | 0 |
| 412 | 5.438 | 0 |
| 413 | 5.438 | 0 |
| 414 | 5.438 | 0 |
| 415 | 5.438 | 0 |
| 416 | 5.438 | 0 |
| 417 | 5.438 | 0 |
| 418 | 5.438 | 0 |
| 419 | 5.438 | 0 |
| 420 | 5.438 | 0 |
| 421 | 5.438 | 0 |
| 422 | 5.438 | 0 |
| 423 | 5.438 | 0 |
| 424 | 5.438 | 0 |
| 425 | 5.438 | 0 |
| 426 | 5.438 | 0 |
| 427 | 5.438 | 0 |
| 428 | 5.438 | 0 |
| 429 | 5.438 | 0 |
| 430 | 5.438 | 0 |
| 431 | 5.438 | 0 |
| 432 | 5.438 | 0 |
| 433 | 5.438 | 0 |
| 434 | 5.438 | 0 |
| 435 | 5.438 | 0 |
| 436 | 5.438 | 0 |
| 437 | 5.438 | 0 |
| 438 | 5.438 | 0 |
| 439 | 5.438 | 0 |
| 440 | 5.438 | 0 |
| 441 | 5.438 | 0 |
| 442 | 5.438 | 0 |
| 443 | 5.438 | 0 |
| 444 | 5.438 | 0 |
| 445 | 5.438 | 0 |
| 446 | 5.438 | 0 |
| 447 | 5.438 | 0 |
| 448 | 5.438 | 0 |
| 449 | 5.438 | 0 |
| 450 | 5.438 | 0 |

Generated patient level data* based on Figure 3b from Yothers et al. (2011)
* Based on the method described by Hoyle and Henley (2011)

| Fluoropyrimidine monotherapy | | | Fluoropyrimidine combined with oxaliplatin | | |
|---|---|---|---|---|---|
| ID | Follow_up | Event | ID | Follow_up | Event |
| 1 | 0.25 | 1 | 1 | 0.25 | 1 |
| 2 | 0.25 | 1 | 2 | 0.25 | 1 |
| 3 | 0.25 | 1 | 3 | 0.25 | 1 |
| 4 | 0.75 | 1 | 4 | 0.25 | 1 |
| 5 | 0.75 | 1 | 5 | 0.25 | 1 |
| 6 | 0.75 | 1 | 6 | 0.75 | 1 |
| 7 | 0.75 | 1 | 7 | 0.75 | 1 |
| 8 | 0.75 | 1 | 8 | 0.75 | 1 |
| 9 | 0.75 | 1 | 9 | 0.75 | 1 |
| 10 | 0.75 | 1 | 10 | 0.75 | 1 |
| 11 | 0.75 | 1 | 11 | 0.75 | 1 |
| 12 | 0.75 | 1 | 12 | 0.75 | 1 |
| 13 | 0.75 | 1 | 13 | 0.75 | 1 |
| 14 | 0.75 | 1 | 14 | 0.75 | 1 |
| 15 | 0.75 | 1 | 15 | 1.25 | 1 |
| 16 | 0.75 | 1 | 16 | 1.25 | 1 |
| 17 | 1.25 | 1 | 17 | 1.25 | 1 |
| 18 | 1.25 | 1 | 18 | 1.25 | 1 |
| 19 | 1.25 | 1 | 19 | 1.25 | 1 |
| 20 | 1.25 | 1 | 20 | 1.25 | 1 |
| 21 | 1.25 | 1 | 21 | 1.25 | 1 |
| 22 | 1.25 | 1 | 22 | 1.75 | 1 |
| 23 | 1.25 | 1 | 23 | 1.75 | 1 |
| 24 | 1.25 | 1 | 24 | 1.75 | 1 |
| 25 | 1.25 | 1 | 25 | 1.75 | 1 |
| 26 | 1.25 | 1 | 26 | 1.75 | 1 |
| 27 | 1.25 | 1 | 27 | 2.25 | 1 |
| 28 | 1.25 | 1 | 28 | 2.25 | 1 |
| 29 | 1.25 | 1 | 29 | 2.25 | 1 |
| 30 | 1.25 | 1 | 30 | 2.25 | 1 |
| 31 | 1.25 | 1 | 31 | 2.25 | 1 |
| 32 | 1.75 | 1 | 32 | 2.25 | 1 |
| 33 | 1.75 | 1 | 33 | 2.75 | 1 |
| 34 | 1.75 | 1 | 34 | 2.75 | 1 |
| 35 | 1.75 | 1 | 35 | 2.75 | 1 |
| 36 | 1.75 | 1 | 36 | 2.75 | 1 |
| 37 | 1.75 | 1 | 37 | 2.75 | 1 |
| 38 | 1.75 | 1 | 38 | 2.75 | 1 |
| 39 | 1.75 | 1 | 39 | 2.75 | 1 |
| 40 | 1.75 | 1 | 40 | 2.75 | 1 |
| 41 | 1.75 | 1 | 41 | 2.75 | 1 |
| 42 | 1.75 | 1 | 42 | 2.75 | 1 |
| 43 | 1.75 | 1 | 43 | 2.75 | 1 |
| 44 | 2.25 | 1 | 44 | 2.75 | 1 |
| 45 | 2.25 | 1 | 45 | 3.25 | 1 |
| 46 | 2.25 | 1 | 46 | 3.25 | 1 |
| 47 | 2.25 | 1 | 47 | 3.25 | 1 |
| 48 | 2.75 | 1 | 48 | 3.25 | 1 |
| 49 | 2.75 | 1 | 49 | 3.25 | 1 |
| 50 | 2.75 | 1 | 50 | 3.75 | 1 |
| 51 | 2.75 | 1 | 51 | 3.75 | 1 |
| 52 | 3.25 | 1 | 52 | 3.75 | 1 |
| 53 | 3.25 | 1 | 53 | 3.75 | 1 |
| 54 | 3.25 | 1 | 54 | 4.25 | 1 |
| 55 | 3.25 | 1 | 55 | 4.25 | 1 |
| 56 | 3.25 | 1 | 56 | 4.25 | 1 |
| 57 | 3.75 | 1 | 57 | 4.25 | 1 |
| 58 | 3.75 | 1 | 58 | 4.25 | 1 |
| 59 | 3.75 | 1 | 59 | 4.75 | 1 |
| 60 | 3.75 | 1 | 60 | 4.75 | 1 |
| 61 | 3.75 | 1 | 61 | 5.25 | 1 |
| 62 | 4.25 | 1 | 62 | 5.25 | 1 |
| 63 | 4.25 | 1 | 63 | 5.25 | 1 |
| 64 | 4.25 | 1 | 64 | 5.25 | 1 |
| 65 | 4.25 | 1 | 65 | 5.25 | 1 |
| 66 | 4.25 | 1 | 66 | 5.25 | 1 |
| 67 | 4.75 | 1 | 67 | 5.75 | 1 |
| 68 | 4.75 | 1 | 68 | 5.75 | 1 |
| 69 | 4.75 | 1 | 69 | 5.75 | 1 |
| 70 | 4.75 | 1 | 70 | 5.75 | 1 |
| 71 | 5.25 | 1 | 71 | 5.75 | 1 |
| 72 | 5.25 | 1 | 72 | 6.25 | 1 |
| 73 | 5.75 | 1 | 73 | 6.25 | 1 |
| 74 | 5.75 | 1 | 74 | 6.25 | 1 |
| 75 | 5.75 | 1 | 75 | 6.25 | 1 |
| 76 | 5.75 | 1 | 76 | 6.25 | 1 |
| 77 | 5.75 | 1 | 77 | 6.75 | 1 |
| 78 | 5.75 | 1 | 78 | 6.75 | 1 |
| 79 | 5.75 | 1 | 79 | 6.75 | 1 |
| 80 | 6.25 | 1 | 80 | 6.75 | 1 |
| 81 | 6.25 | 1 | 81 | 7.25 | 1 |
| 82 | 6.25 | 1 | 82 | 7.25 | 1 |
| 83 | 6.75 | 1 | 83 | 7.25 | 1 |
| 84 | 7.25 | 1 | 84 | 7.75 | 1 |
| 85 | 7.25 | 1 | 85 | 0.25 | 0 |

| | |
|---|---|
| ID | Patient ID |
| Follow_up | Follow up time in years |
| Event | 0 = no recurrence |
| | 1 = recurrence |

| | | |
|---|---|---|
| 86 | 7.75 | 1 |
| 87 | 7.75 | 1 |
| 88 | 7.75 | 1 |
| 89 | 2.25 | 0 |
| 90 | 2.25 | 0 |
| 91 | 2.75 | 0 |
| 92 | 2.75 | 0 |
| 93 | 3.25 | 0 |
| 94 | 3.25 | 0 |
| 95 | 3.75 | 0 |
| 96 | 3.75 | 0 |
| 97 | 4.25 | 0 |
| 98 | 4.25 | 0 |
| 99 | 4.75 | 0 |
| 100 | 4.75 | 0 |
| 101 | 5.25 | 0 |
| 102 | 5.25 | 0 |
| 103 | 5.75 | 0 |
| 104 | 5.75 | 0 |
| 105 | 6.25 | 0 |
| 106 | 6.25 | 0 |
| 107 | 6.25 | 0 |
| 108 | 6.25 | 0 |
| 109 | 6.25 | 0 |
| 110 | 6.25 | 0 |
| 111 | 6.25 | 0 |
| 112 | 6.25 | 0 |
| 113 | 6.25 | 0 |
| 114 | 6.25 | 0 |
| 115 | 6.25 | 0 |
| 116 | 6.25 | 0 |
| 117 | 6.25 | 0 |
| 118 | 6.25 | 0 |
| 119 | 6.25 | 0 |
| 120 | 6.25 | 0 |
| 121 | 6.25 | 0 |
| 122 | 6.25 | 0 |
| 123 | 6.25 | 0 |
| 124 | 6.25 | 0 |
| 125 | 6.25 | 0 |
| 126 | 6.25 | 0 |
| 127 | 6.25 | 0 |
| 128 | 6.25 | 0 |
| 129 | 6.75 | 0 |
| 130 | 6.75 | 0 |
| 131 | 6.75 | 0 |
| 132 | 6.75 | 0 |
| 133 | 6.75 | 0 |
| 134 | 6.75 | 0 |
| 135 | 6.75 | 0 |
| 136 | 6.75 | 0 |
| 137 | 6.75 | 0 |
| 138 | 6.75 | 0 |
| 139 | 6.75 | 0 |
| 140 | 6.75 | 0 |
| 141 | 6.75 | 0 |
| 142 | 6.75 | 0 |
| 143 | 6.75 | 0 |
| 144 | 6.75 | 0 |
| 145 | 6.75 | 0 |
| 146 | 6.75 | 0 |
| 147 | 6.75 | 0 |
| 148 | 6.75 | 0 |
| 149 | 6.75 | 0 |
| 150 | 6.75 | 0 |
| 151 | 6.75 | 0 |
| 152 | 6.75 | 0 |
| 153 | 7.25 | 0 |
| 154 | 7.25 | 0 |
| 155 | 7.25 | 0 |
| 156 | 7.25 | 0 |
| 157 | 7.25 | 0 |
| 158 | 7.25 | 0 |
| 159 | 7.25 | 0 |
| 160 | 7.25 | 0 |
| 161 | 7.25 | 0 |
| 162 | 7.25 | 0 |
| 163 | 7.25 | 0 |
| 164 | 7.25 | 0 |
| 165 | 7.25 | 0 |
| 166 | 7.25 | 0 |
| 167 | 7.25 | 0 |
| 168 | 7.25 | 0 |
| 169 | 7.25 | 0 |
| 170 | 7.25 | 0 |
| 171 | 7.25 | 0 |
| 172 | 7.25 | 0 |
| 173 | 7.25 | 0 |
| 174 | 7.25 | 0 |
| 175 | 7.25 | 0 |
| 176 | 7.25 | 0 |

| | | |
|---|---|---|
| 86 | 0.25 | 0 |
| 87 | 0.75 | 0 |
| 88 | 0.75 | 0 |
| 89 | 1.25 | 0 |
| 90 | 1.25 | 0 |
| 91 | 1.75 | 0 |
| 92 | 1.75 | 0 |
| 93 | 2.25 | 0 |
| 94 | 2.75 | 0 |
| 95 | 3.25 | 0 |
| 96 | 3.75 | 0 |
| 97 | 4.25 | 0 |
| 98 | 4.25 | 0 |
| 99 | 4.75 | 0 |
| 100 | 4.75 | 0 |
| 101 | 4.75 | 0 |
| 102 | 5.25 | 0 |
| 103 | 5.25 | 0 |
| 104 | 5.25 | 0 |
| 105 | 5.75 | 0 |
| 106 | 5.75 | 0 |
| 107 | 5.75 | 0 |
| 108 | 6.25 | 0 |
| 109 | 6.25 | 0 |
| 110 | 6.25 | 0 |
| 111 | 6.25 | 0 |
| 112 | 6.25 | 0 |
| 113 | 6.25 | 0 |
| 114 | 6.25 | 0 |
| 115 | 6.25 | 0 |
| 116 | 6.25 | 0 |
| 117 | 6.25 | 0 |
| 118 | 6.25 | 0 |
| 119 | 6.25 | 0 |
| 120 | 6.25 | 0 |
| 121 | 6.25 | 0 |
| 122 | 6.25 | 0 |
| 123 | 6.25 | 0 |
| 124 | 6.25 | 0 |
| 125 | 6.25 | 0 |
| 126 | 6.25 | 0 |
| 127 | 6.25 | 0 |
| 128 | 6.25 | 0 |
| 129 | 6.25 | 0 |
| 130 | 6.25 | 0 |
| 131 | 6.25 | 0 |
| 132 | 6.25 | 0 |
| 133 | 6.75 | 0 |
| 134 | 6.75 | 0 |
| 135 | 6.75 | 0 |
| 136 | 6.75 | 0 |
| 137 | 6.75 | 0 |
| 138 | 6.75 | 0 |
| 139 | 6.75 | 0 |
| 140 | 6.75 | 0 |
| 141 | 6.75 | 0 |
| 142 | 6.75 | 0 |
| 143 | 6.75 | 0 |
| 144 | 6.75 | 0 |
| 145 | 6.75 | 0 |
| 146 | 6.75 | 0 |
| 147 | 6.75 | 0 |
| 148 | 6.75 | 0 |
| 149 | 6.75 | 0 |
| 150 | 6.75 | 0 |
| 151 | 6.75 | 0 |
| 152 | 6.75 | 0 |
| 153 | 6.75 | 0 |
| 154 | 6.75 | 0 |
| 155 | 6.75 | 0 |
| 156 | 6.75 | 0 |
| 157 | 6.75 | 0 |
| 158 | 7.25 | 0 |
| 159 | 7.25 | 0 |
| 160 | 7.25 | 0 |
| 161 | 7.25 | 0 |
| 162 | 7.25 | 0 |
| 163 | 7.25 | 0 |
| 164 | 7.25 | 0 |
| 165 | 7.25 | 0 |
| 166 | 7.25 | 0 |
| 167 | 7.25 | 0 |
| 168 | 7.25 | 0 |
| 169 | 7.25 | 0 |
| 170 | 7.25 | 0 |
| 171 | 7.25 | 0 |
| 172 | 7.25 | 0 |
| 173 | 7.25 | 0 |
| 174 | 7.25 | 0 |
| 175 | 7.25 | 0 |
| 176 | 7.25 | 0 |

| | | |
|---|---|---|
| 177 | 7.75 | 0 |
| 178 | 7.75 | 0 |
| 179 | 7.75 | 0 |
| 180 | 7.75 | 0 |
| 181 | 7.75 | 0 |
| 182 | 7.75 | 0 |
| 183 | 7.75 | 0 |
| 184 | 7.75 | 0 |
| 185 | 7.75 | 0 |
| 186 | 7.75 | 0 |
| 187 | 7.75 | 0 |
| 188 | 7.75 | 0 |
| 189 | 7.75 | 0 |
| 190 | 7.75 | 0 |
| 191 | 7.75 | 0 |
| 192 | 7.75 | 0 |
| 193 | 7.75 | 0 |
| 194 | 7.75 | 0 |
| 195 | 7.75 | 0 |
| 196 | 7.75 | 0 |
| 197 | 7.75 | 0 |
| 198 | 7.75 | 0 |
| 199 | 7.75 | 0 |
| 200 | 7.75 | 0 |
| 201 | 7.75 | 0 |
| 202 | 7.75 | 0 |
| 203 | 7.75 | 0 |
| 204 | 7.75 | 0 |
| 205 | 7.75 | 0 |
| 206 | 7.75 | 0 |
| 207 | 7.75 | 0 |
| 208 | 7.75 | 0 |
| 209 | 7.75 | 0 |
| 210 | 7.75 | 0 |
| 211 | 7.75 | 0 |
| 212 | 7.75 | 0 |
| 213 | 7.75 | 0 |
| 214 | 7.75 | 0 |
| 215 | 7.75 | 0 |
| 216 | 7.75 | 0 |
| 217 | 7.75 | 0 |
| 218 | 7.75 | 0 |
| 219 | 7.75 | 0 |
| 220 | 7.75 | 0 |
| 221 | 7.75 | 0 |
| 222 | 7.75 | 0 |
| 223 | 7.75 | 0 |
| 224 | 7.75 | 0 |
| 225 | 7.75 | 0 |
| 226 | 7.75 | 0 |
| 227 | 7.75 | 0 |
| 228 | 7.75 | 0 |
| 229 | 7.75 | 0 |
| 230 | 7.75 | 0 |
| 231 | 7.75 | 0 |
| 232 | 7.75 | 0 |
| 233 | 7.75 | 0 |
| 234 | 7.75 | 0 |
| 235 | 7.75 | 0 |
| 236 | 7.75 | 0 |
| 237 | 7.75 | 0 |
| 238 | 7.75 | 0 |
| 239 | 7.75 | 0 |
| 240 | 7.75 | 0 |
| 241 | 7.75 | 0 |
| 242 | 7.75 | 0 |
| 243 | 7.75 | 0 |
| 244 | 7.75 | 0 |
| 245 | 7.75 | 0 |
| 246 | 7.75 | 0 |
| 247 | 7.75 | 0 |
| 248 | 7.75 | 0 |
| 249 | 7.75 | 0 |
| 250 | 7.75 | 0 |
| 251 | 7.75 | 0 |
| 252 | 7.75 | 0 |
| 253 | 7.75 | 0 |
| 254 | 7.75 | 0 |
| 255 | 7.75 | 0 |
| 256 | 7.75 | 0 |
| 257 | 7.75 | 0 |
| 258 | 7.75 | 0 |
| 259 | 7.75 | 0 |
| 260 | 7.75 | 0 |
| 261 | 7.75 | 0 |
| 262 | 7.75 | 0 |
| 263 | 7.75 | 0 |
| 264 | 7.75 | 0 |
| 265 | 7.75 | 0 |
| 266 | 7.75 | 0 |
| 267 | 7.75 | 0 |

| | | |
|---|---|---|
| 177 | 7.25 | 0 |
| 178 | 7.25 | 0 |
| 179 | 7.25 | 0 |
| 180 | 7.25 | 0 |
| 181 | 7.25 | 0 |
| 182 | 7.25 | 0 |
| 183 | 7.75 | 0 |
| 184 | 7.75 | 0 |
| 185 | 7.75 | 0 |
| 186 | 7.75 | 0 |
| 187 | 7.75 | 0 |
| 188 | 7.75 | 0 |
| 189 | 7.75 | 0 |
| 190 | 7.75 | 0 |
| 191 | 7.75 | 0 |
| 192 | 7.75 | 0 |
| 193 | 7.75 | 0 |
| 194 | 7.75 | 0 |
| 195 | 7.75 | 0 |
| 196 | 7.75 | 0 |
| 197 | 7.75 | 0 |
| 198 | 7.75 | 0 |
| 199 | 7.75 | 0 |
| 200 | 7.75 | 0 |
| 201 | 7.75 | 0 |
| 202 | 7.75 | 0 |
| 203 | 7.75 | 0 |
| 204 | 7.75 | 0 |
| 205 | 7.75 | 0 |
| 206 | 7.75 | 0 |
| 207 | 7.75 | 0 |
| 208 | 7.75 | 0 |
| 209 | 7.75 | 0 |
| 210 | 7.75 | 0 |
| 211 | 7.75 | 0 |
| 212 | 7.75 | 0 |
| 213 | 7.75 | 0 |
| 214 | 7.75 | 0 |
| 215 | 7.75 | 0 |
| 216 | 7.75 | 0 |
| 217 | 7.75 | 0 |
| 218 | 7.75 | 0 |
| 219 | 7.75 | 0 |
| 220 | 7.75 | 0 |
| 221 | 7.75 | 0 |
| 222 | 7.75 | 0 |
| 223 | 7.75 | 0 |
| 224 | 7.75 | 0 |
| 225 | 7.75 | 0 |
| 226 | 7.75 | 0 |
| 227 | 7.75 | 0 |
| 228 | 7.75 | 0 |
| 229 | 7.75 | 0 |
| 230 | 7.75 | 0 |
| 231 | 7.75 | 0 |
| 232 | 7.75 | 0 |
| 233 | 7.75 | 0 |
| 234 | 7.75 | 0 |
| 235 | 7.75 | 0 |
| 236 | 7.75 | 0 |
| 237 | 7.75 | 0 |
| 238 | 7.75 | 0 |
| 239 | 7.75 | 0 |
| 240 | 7.75 | 0 |
| 241 | 7.75 | 0 |
| 242 | 7.75 | 0 |
| 243 | 7.75 | 0 |
| 244 | 7.75 | 0 |
| 245 | 7.75 | 0 |
| 246 | 7.75 | 0 |
| 247 | 7.75 | 0 |
| 248 | 7.75 | 0 |
| 249 | 7.75 | 0 |
| 250 | 7.75 | 0 |
| 251 | 7.75 | 0 |
| 252 | 7.75 | 0 |
| 253 | 7.75 | 0 |
| 254 | 7.75 | 0 |
| 255 | 7.75 | 0 |
| 256 | 7.75 | 0 |
| 257 | 7.75 | 0 |
| 258 | 7.75 | 0 |
| 259 | 7.75 | 0 |
| 260 | 7.75 | 0 |
| 261 | 7.75 | 0 |
| 262 | 7.75 | 0 |
| 263 | 7.75 | 0 |
| 264 | 7.75 | 0 |
| 265 | 7.75 | 0 |
| 266 | 7.75 | 0 |
| 267 | 7.75 | 0 |

| | | |
|---|---|---|
| 268 | 7.75 | 0 |
| 269 | 7.75 | 0 |
| 270 | 7.75 | 0 |
| 271 | 7.75 | 0 |
| 272 | 7.75 | 0 |
| 273 | 7.75 | 0 |
| 274 | 7.75 | 0 |
| 275 | 7.75 | 0 |
| 276 | 7.75 | 0 |
| 277 | 7.75 | 0 |
| 278 | 7.75 | 0 |
| 279 | 7.75 | 0 |
| 280 | 7.75 | 0 |
| 281 | 7.75 | 0 |
| 282 | 7.75 | 0 |
| 283 | 7.75 | 0 |
| 284 | 7.75 | 0 |
| 285 | 7.75 | 0 |
| 286 | 7.75 | 0 |
| 287 | 7.75 | 0 |
| 288 | 7.75 | 0 |
| 289 | 7.75 | 0 |
| 290 | 7.75 | 0 |
| 291 | 7.75 | 0 |
| 292 | 7.75 | 0 |
| 293 | 7.75 | 0 |
| 294 | 7.75 | 0 |
| 295 | 7.75 | 0 |
| 296 | 7.75 | 0 |
| 297 | 7.75 | 0 |
| 298 | 7.75 | 0 |
| 299 | 7.75 | 0 |
| 300 | 7.75 | 0 |
| 301 | 7.75 | 0 |
| 302 | 7.75 | 0 |
| 303 | 7.75 | 0 |
| 304 | 7.75 | 0 |
| 305 | 7.75 | 0 |
| 306 | 7.75 | 0 |
| 307 | 7.75 | 0 |
| 308 | 7.75 | 0 |
| 309 | 7.75 | 0 |
| 310 | 7.75 | 0 |
| 311 | 7.75 | 0 |
| 312 | 7.75 | 0 |
| 313 | 7.75 | 0 |
| 314 | 7.75 | 0 |
| 315 | 7.75 | 0 |
| 316 | 7.75 | 0 |
| 317 | 7.75 | 0 |
| 318 | 7.75 | 0 |
| 319 | 7.75 | 0 |
| 320 | 7.75 | 0 |
| 321 | 7.75 | 0 |
| 322 | 7.75 | 0 |
| 323 | 7.75 | 0 |
| 324 | 7.75 | 0 |
| 325 | 7.75 | 0 |
| 326 | 7.75 | 0 |
| 327 | 7.75 | 0 |
| 328 | 7.75 | 0 |
| 329 | 7.75 | 0 |
| 330 | 7.75 | 0 |
| 331 | 7.75 | 0 |
| 332 | 7.75 | 0 |
| 333 | 7.75 | 0 |
| 334 | 7.75 | 0 |
| 335 | 7.75 | 0 |
| 336 | 7.75 | 0 |
| 337 | 7.75 | 0 |
| 338 | 7.75 | 0 |
| 339 | 7.75 | 0 |
| 340 | 7.75 | 0 |
| 341 | 7.75 | 0 |
| 342 | 7.75 | 0 |
| 343 | 7.75 | 0 |
| 344 | 7.75 | 0 |
| 345 | 7.75 | 0 |
| 346 | 7.75 | 0 |
| 347 | 7.75 | 0 |

| | | |
|---|---|---|
| 268 | 7.75 | 0 |
| 269 | 7.75 | 0 |
| 270 | 7.75 | 0 |
| 271 | 7.75 | 0 |
| 272 | 7.75 | 0 |
| 273 | 7.75 | 0 |
| 274 | 7.75 | 0 |
| 275 | 7.75 | 0 |
| 276 | 7.75 | 0 |
| 277 | 7.75 | 0 |
| 278 | 7.75 | 0 |
| 279 | 7.75 | 0 |
| 280 | 7.75 | 0 |
| 281 | 7.75 | 0 |
| 282 | 7.75 | 0 |
| 283 | 7.75 | 0 |
| 284 | 7.75 | 0 |
| 285 | 7.75 | 0 |
| 286 | 7.75 | 0 |
| 287 | 7.75 | 0 |
| 288 | 7.75 | 0 |
| 289 | 7.75 | 0 |
| 290 | 7.75 | 0 |
| 291 | 7.75 | 0 |
| 292 | 7.75 | 0 |
| 293 | 7.75 | 0 |
| 294 | 7.75 | 0 |
| 295 | 7.75 | 0 |
| 296 | 7.75 | 0 |
| 297 | 7.75 | 0 |
| 298 | 7.75 | 0 |
| 299 | 7.75 | 0 |
| 300 | 7.75 | 0 |
| 301 | 7.75 | 0 |
| 302 | 7.75 | 0 |
| 303 | 7.75 | 0 |
| 304 | 7.75 | 0 |
| 305 | 7.75 | 0 |
| 306 | 7.75 | 0 |
| 307 | 7.75 | 0 |
| 308 | 7.75 | 0 |
| 309 | 7.75 | 0 |
| 310 | 7.75 | 0 |
| 311 | 7.75 | 0 |
| 312 | 7.75 | 0 |
| 313 | 7.75 | 0 |
| 314 | 7.75 | 0 |
| 315 | 7.75 | 0 |
| 316 | 7.75 | 0 |
| 317 | 7.75 | 0 |
| 318 | 7.75 | 0 |
| 319 | 7.75 | 0 |
| 320 | 7.75 | 0 |
| 321 | 7.75 | 0 |
| 322 | 7.75 | 0 |
| 323 | 7.75 | 0 |
| 324 | 7.75 | 0 |
| 325 | 7.75 | 0 |
| 326 | 7.75 | 0 |
| 327 | 7.75 | 0 |
| 328 | 7.75 | 0 |
| 329 | 7.75 | 0 |
| 330 | 7.75 | 0 |
| 331 | 7.75 | 0 |
| 332 | 7.75 | 0 |
| 333 | 7.75 | 0 |
| 334 | 7.75 | 0 |
| 335 | 7.75 | 0 |
| 336 | 7.75 | 0 |
| 337 | 7.75 | 0 |
| 338 | 7.75 | 0 |
| 339 | 7.75 | 0 |
| 340 | 7.75 | 0 |
| 341 | 7.75 | 0 |
| 342 | 7.75 | 0 |
| 343 | 7.75 | 0 |
| 344 | 7.75 | 0 |
| 345 | 7.75 | 0 |
| 346 | 7.75 | 0 |
| 347 | 7.75 | 0 |
| 348 | 7.75 | 0 |
| 349 | 7.75 | 0 |
